# *Ortho*‐Carborane Decorated Multi‐Resonance TADF Emitters: Preserving Local Excited State and High Efficiency in OLEDs

**DOI:** 10.1002/advs.202309016

**Published:** 2024-01-17

**Authors:** Taehwan Lee, Jee‐Hun Jang, Nhi Ngoc Tuyet Nguyen, Jaehoon Jung, Jeong‐Hwan Lee, Min Hyung Lee

**Affiliations:** ^1^ Department of Chemistry University of Ulsan Ulsan 44610 Republic of Korea; ^2^ Department of Materials Science and Engineering and 3D Convergence Center Inha University Incheon 22212 Republic of Korea

**Keywords:** blue OLEDs, local emission, multi‐resonance, *o*‐carborane, TADF

## Abstract

A novel class of *o*‐carboranyl luminophores, 2CB‐BuDABNA (**1**) and 3CB‐BuDABNA (**2**) is reported, in which *o*‐carborane moieties are incorporated at the periphery of the B,N‐doped multi‐resonance thermally activated delayed fluorescence (MR‐TADF) core. Both compounds maintain the inherent local emission characteristics of their MR‐emitting core, exhibiting intense MR‐TADF with high photoluminescence quantum yields in toluene and rigid states. In contrast, the presence of the dark lowest‐energy charge transfer state, induced by cage rotation in THF, is suggested to be responsible for emission quenching in a polar solvent. Despite the different arrangement of the cage on the DABNA core, both **1** and **2** show red‐shifted emissions compared to the parent compound BuDABNA (**3**). By utilizing **1** as the emitter, high‐efficiency blue organic light‐emitting diodes (OLEDs) are achieved with a remarkable maximum external quantum efficiency of 25%, representing the highest reported efficiency for OLEDs employing an *o*‐carboranyl luminophore as the emitter.

## Graphical abstract

*Ortho*‐carborane decorated multi‐resonance induced thermally activated delayed fluorescence (MR‐TADF) emitters are developed. The inherent local emission characteristics of the MR‐emitting core are preserved while being tuned through *o*‐carborane substitution. The resulting blue OLEDs achieve a remarkable maximum external quantum efficiency of 25%, marking the first demonstration of outstanding OLED performance utilizing an *o*‐carboranyl luminophore as the emitter.

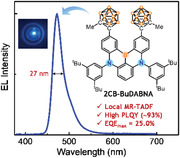

## 1 Introduction

**Figure 1:**
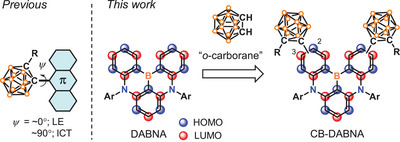
Structures of previous *o*‐carboranyl fluorophores (left) and *o*‐carborane decorated MR‐TADF emitters (right).

*Ortho*‐carborane (1,2‐*closo*‐C_2_B_10_H_12_), an electron‐deficient icosahedral boron cluster, has garnered considerable attention as a steric and electronic building block for constructing various luminophores over the past decade^[^ ^1^ ^]^ due to its steric bulkiness and 3D electron delocalization through three‐center, two‐electron bonds.^[^ ^2^ ^]^ The emissive excited state of the *o*‐carboranyl luminophores can be accessed through the locally excited (LE) or intramolecular charge transfer (ICT) states, depending on the degree of cage rotation (**Figure** **1**, left).^[^ ^1^, ^3^ ^]^ Specifically, when the C_CB_−C_CB_ bond is oriented perpendicular to the π‐system (*ψ* = ∼90°), the ICT state is favored due to the favorable σ*(C_CB_−C_CB_)−π* conjugation that results in the formation of lowest unoccupied molecular orbitals (LUMOs).^[^ ^1^, ^4^ ^]^ This electronic interaction between the carboranyl σ* orbital and the delocalized π* orbital of an aromatic luminophore significantly influences the LUMO of the luminophores, thereby altering their excited‐state properties. Consequently, *o*‐carboranyl luminophores, especially those featuring a 2‐R‐substituent on the cage, predominantly exhibit broad CT emissions in the low‐energy region.

In the context of developing novel *o*‐carboranyl luminophores for optoelectronic materials applications, such as solid‐state emitting^[^ ^5^ ^]^ and stimuli‐responsive materials,^[^ ^6^ ^]^ it remains challenging to understand the role of *o*‐carborane in controlling the photophysical properties of various luminophores. Since the electronic effects of *o*‐carborane can vary depending on the π‐systems used, the choice of the parent π‐luminophore becomes a critical factor in tuning the excited‐state properties. With this in mind, we decided to employ a multi‐resonance induced thermally activated delayed fluorescence (MR‐TADF) core as a new luminophoric π‐skeleton to create novel *o*‐carboranyl TADF emitters (Figure 1, right). Due to the alternate localization of the highest occupied molecular orbital (HOMO) and LUMO on different atoms, resulting in a short‐range CT (SRCT) transition, MR‐TADF emitters typically exhibit narrowband emissions.^[^ ^7^ ^]^ Therefore, one can anticipate that the electronic coupling between the *o*‐carborane and the B,N‐doped MR‐emitting core may induce distinct excited‐state properties compared to conventional *o*‐carboranyl luminophores. Additionally, the introduction of *o*‐carborane into either the HOMO‐ or LUMO‐dominant positions may have a varying impact on the excited state. To unveil the effects of *o*‐carborane on the photophysical and electroluminescent properties of MR‐TADF emitters, we herein report two *o*‐carborane‐appended MR‐TADF emitters, namely 2CB‐BuDABNA (**1**) and 3CB‐BuDABNA (**2**), along with a reference emitter, BuDABNA (**3**). We found that the inherent local emission characteristics of the MR‐emitting core are retained but tuned through *o*‐carborane substitution. Computational results also provide a rationalization of the observed photophysical properties of **1** and **2**, including local emission, redshift in absorption and emission, and spectral quenching in a polar medium. Furthermore, the OLED devices incorporating **1** as the emitter achieve a high maximum external quantum efficiency (EQE_max_) of 25.0%. This study represents the first example demonstrating excellent OLED performance by utilizing an *o*‐carboranyl luminophore as the emitter.

## 2 Results and Discussion

### 2.1 Synthesis and Characterization

**Scheme 1:**
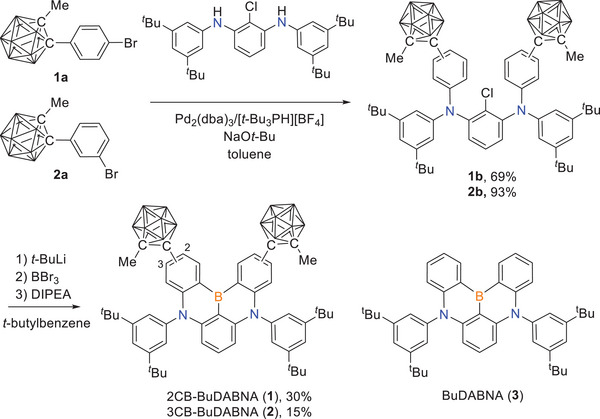
Synthesis of 2CB‐BuDABNA (**1**) and 3CB‐BuDABNA (**2**).

2CB‐BuDABNA (**1**) and 3CB‐BuDABNA (**2**) were prepared following the reactions outlined in **Scheme** **1**. Buchwald−Hartwig amination reactions of 4‐ and 3‐bromophenyl substituted methyl‐*o*‐carboranes (**1a** and **2a**, respectively) with 2‐chloro‐1,3‐diamine led to the *o*‐carborane substituted ligands **1b** and **2b**, respectively. One pot borylation^[^ ^7^, ^8^ ^]^ of the carboranyl ligands (**1b** and **2b**) produced the final B,N‐doped MR‐TADF emitters, **1** and **2**. For comparison, the reference emitter, BuDABNA (**3**), lacking carborane moieties, was prepared analogously (see the Experimental Section and Supporting Information for synthetic details).

**Figure 2:**
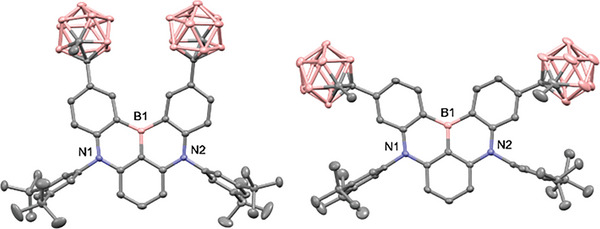
X‐ray crystal structures of 2CB‐BuDABNA (**1**) (left) and 3CB‐BuDABNA (**2**) (right). Dihedral angle (*ψ* = C_CB_−C_CB_−C_Ph_−C_Ph_): 75.2(4)° for **1**; 74.1(3)° and 72.1(3)° for **2**. C_CB_−C_CB_ bond distances: 1.698(5) Å for **1**; 1.688(3) and 1.693(3) Å for **2**.

Emitters **1** and **2** exhibited high thermal decomposition temperatures (*T*_d5_ = 382−400 °C, at 5 wt.% decomposition), significantly exceeding that of **3** (303 °C). The elevated stability can be attributed to the high chemical and thermal stability of the *o*‐carborane cage, as well as the steric protection of the MR‐core by the cage. ^11^B NMR spectroscopy confirmed the presence of both the trigonal boron atom of the MR‐core (δ 30−42 ppm) and carboranyl boron atoms (δ −4 to −10 ppm) in **1** and **2**. The crystal structure of **1** revealed that the two methyl groups on the carborane cages were oriented in the opposite direction with respect to the plane of the B,N‐core, providing large steric protection (**Figure** **2**). In contrast, the two carboranyl methyl groups in **2** were held on the same side of the plane. The carboranyl C−C bond distances (1.698(5) Å for **1** and 1.688(3)−1.693(3) Å for **2**) were in the typical range for carboranyl luminophores. The large dihedral angles (*ψ* = C_CB_−C_CB_−C_Ph_−C_Ph_) between the carborane and phenylene ring in **1** and **2** indicated a perpendicular orientation of the carboranyl C−C bond axis toward the phenylene ring.

### 2.2 Photophysical Properties

**Figure 3:**
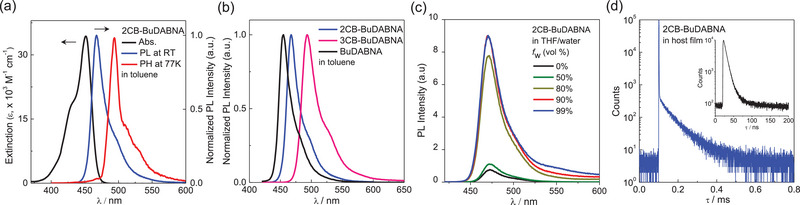
a) UV/Vis absorption, PL, and phosphorescence (PH) spectra of 2CB‐BuDABNA (**1**) in toluene (2.0 × 10^−5^ m). b) PL spectra of **1**, 3CB‐BuDABNA (**2**), and BuDABNA (**3**) in toluene at RT. c) PL spectra of **1** in THF/water mixtures (2.0 × 10^−5^ m) with various water fractions (*f*_w_). d) Transient PL decay of the SiCzCz:SiTrzCz2 host film doped with 5 wt.% of **1**. Inset: prompt PL decay.

The photophysical properties of **1**−**3** were first investigated in toluene (**Figure** **3** and **Table** **1**). The strong SRCT absorption in both **1** (451 nm) and **2** (476 nm) exhibited a redshift compared to reference **3** (442 nm). Inspection of the experimental HOMO and LUMO levels indicated that the LUMO stabilization by carborane substitution is greater than the HOMO stabilization in **1** and **2** when compared to the corresponding levels in **3** (Figure S7, Supporting Information). This finding suggests that, unlike the distinct positional electronic effects on either HOMO or LUMO in previous luminophores,^[^ ^1^, ^9^ ^]^ *o*‐carborane substitution on the MR‐core has a more pronounced impact on LUMO stabilization. The PL spectra of all compounds exhibited narrowband emissions typical for B,N‐doped MR‐emitters. The deep blue emission peak of **3** (455 nm) underwent redshifts in **1** (467 nm) and **2** (493 nm) (Figure 3b). The significant redshift in **2** indicates a substantial stabilization of the singlet excited state (S_1_) through carborane substitution at the 3‐position. Notably, no ICT band other than the local emission was detected in **1** and **2**, indicating that the carborane moiety does not alter the emissive SRCT states of the MR‐core in toluene. The narrow full width at half maximum (FWHM) of the emission in **3** (22 nm) was maintained in **1** (22 nm) while it was slightly broadened in **2** (29 nm). The PL quantum yields (PLQY, *Φ*_PL_) of **1** and **2** also remained high (76−80%).

**Table 1:** Photophysical data of **1**−**3**.

compd	medium	*λ*_abs_ [nm]	*λ*_PL_ [nm]	*Φ*_PL_ [%]^d)^	FWHM [nm]^e)^	*τ*_p_ [ns]^f)^	*τ*_d_ [µs]^f)^	*E*_S_ eV]^g)^	*E*_T_ eV]^g)^	Δ*E*_ST_ [eV]^g)^
**1**	Tol^a)^	451	467	80	22	8.35		2.64	2.51	0.13
	THF^b)^	452	472	3	28	6.45		2.64	2.51	0.13
	PMMA^c)^		468	76	27	5.97	50.2	2.63	2.48	0.15
**2**	Tol	476	493	76	29	9.24		2.51	2.36	0.15
	THF	475	499	1	48	7.67		2.49	2.34	0.15
	PMMA		495	26	32	5.59	56.4	2.48	2.31	0.17
**3**	Tol	442	455	92	22	8.26		2.73	2.56	0.17
	THF	442	455	91	22	8.95		2.73	2.56	0.17
	PMMA		452	75	29	5.68	94.9	2.70	2.53	0.17

^a)^ In oxygen‐free toluene at 298 K (2.0 × 10^−5^ m);

^b)^ In oxygen‐free THF at 298 K (2.0 × 10^−5^ m);

^c)^ 5 wt.%‐doped film in PMMA;

^d)^ Absolute PLQYs;

^e)^ Full width at half maximum of the PL spectrum;

^f)^ PL lifetimes of prompt (*τ*_p_) and delayed (*τ*_d_) decay components;

^g)^ Δ*E*_ST_ = *E*_S_ − *E*_T_. Singlet (*E*_S_) and triplet (*E*_T_) energies were estimated from the fluorescence and phosphorescence spectra at 77 K.

In a polar THF medium, however, **1** and **2** became poorly emissive, whereas emitter **3** maintained a high PLQY (Table 1). The persistence of the local emission with comparable S_1_ and triplet (T_1_) energies to those in toluene suggests that the emission quenching can be attributed to the fast nonradiative decay of the S_1_ states, triggered by C−C bond variations coupled with the dihedral rotation of the carborane moiety in THF (see computation details below).^[^ ^1^, ^10^ ^]^ However, at 77 K, a broad emission band was concurrently observed for **1** in the lower energy region, whereas **2** did not exhibit it (Figures S12b,S13b, Supporting Information). This band could be attributed to the ICT state formed between the MR‐core and the carboranyl σ* orbital. The absence of such a band in **2** suggests that the ICT state persists at low temperatures in the sterically hindered **1**. Corroborating the weak emission in THF, both **1** and **2** exhibited aggregation‐induced emission (AIE) behavior in a THF/water mixture (Figure 3c).^[^ ^11^ ^]^ With an increase in the water fraction in a THF solution, particularly over 80% water, insoluble aggregates began to form, displaying strong emissions. The nearly consistent emission peak wavelength and spectral broadening indicate the predominance of an identical emitting species in both the solution and aggregate states, ruling out the formation of low‐energy aggregates. This prompted further investigation into the emission properties of **1**−**3** in a rigid matrix, such as PMMA (Figure S16, Supporting Information). Emitters **1** and **2** exhibited slightly blue‐shifted emissions with narrowed FWHMs compared to those observed in THF. Remarkably, the PLQYs of both emitters were significantly boosted in PMMA films, with the PLQY of **1** (76%) nearly comparable to that of emitter **3**. This suggests that the restricted rotation of the carborane enhances the PLQY.^[^ ^1i^ ^]^ The observation of the delayed component in the transient PL decay further implies the retention of the TADF property of the MR‐emitting core in **1** and **2**.

### 2.3 Theoretical Studies

**Figure 4:**
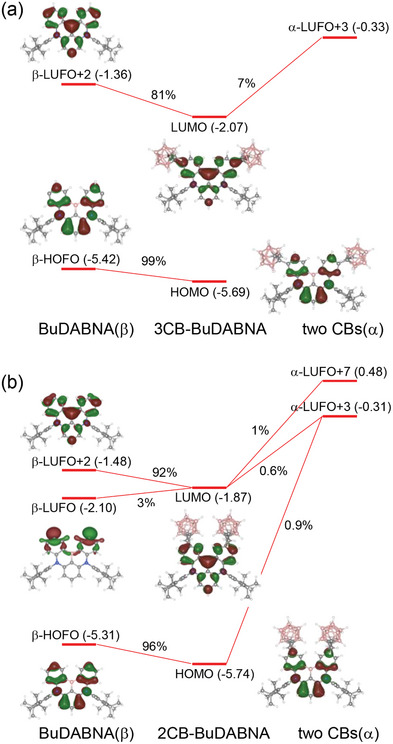
Fragment orbital (FO) diagrams for a) 3CB‐BuDABNA (**2**) and b) 2CB‐BuDABNA (**1**), which are constructed only using the significant electronic couplings between BuDABNA(β) and two CBs(α). The orbital energies (in eV) are presented in parentheses and the contributions (in %) of FOs to HOMO and LUMO are provided. The FOs of two CBs involved in the coupling with BuDABNA are omitted for clarity. (See Figures S19,S20 (Supporting Information) for a full description of FO diagrams for **2** and **1**, respectively).

To elucidate the electronic and photophysical properties of **1** and **2**, computational studies based on time‐dependent DFT (TDDFT) were conducted for all the compounds (see Supporting Information for computation details). The frontier molecular orbitals (FMOs) of all compounds in the ground state (S_0_) were mainly localized on the DABNA backbone with alternating HOMO and LUMO distributions (Figure S18 and Table S2, Supporting Information). For both **1** and **2**, the *o*‐carborane moieties contributed more significantly to the LUMO than to the HOMO, resulting in a more efficient stabilization of the LUMO compared to the HOMO. Thus, their reduced HOMO−LUMO energy gap (*E*_g_) by 0.06 and 0.31 eV compared to the *E*_g_ of **3** leads to redshifts in the absorption and emission spectra of **1** and **2** relative to those of **3**. The larger stabilization of the LUMO than the HOMO in **2** can be readily understood with *o*‐carborane substitution at the LUMO‐dominant 3‐position (see Figure 1). However, the LUMO of **1** is unexpectedly more stabilized than its HOMO, despite *o*‐carborane being introduced at the HOMO‐dominant 2‐position. Fragment orbital (FO) analysis was conducted to unveil the electronic interactions leading to the formation of FMOs (**Figure** **4**). As expected, strong electronic coupling between the MR‐core and *o*‐carborane was observed for the LUMO of **2**. For **1**, recognizable electronic couplings leading to the LUMO were found between the lowest unoccupied fragment orbitals (LUFOs) of MR‐core and *o*‐carborane, as well as the HOMO formed via strong electronic coupling. Particularly, the feasibility of a σ‐orbital contribution to the LUMO of **1**, as identified from the spatial distribution of β‐LUFO of MR‐core (Figure 4b), which was not observed in **2**, would be considered to extraordinarily stabilize the LUMO of **1** compared to its HOMO, leading to the reduction of the *E*_g_ of **1**. The natural transition orbital (NTO) analysis revealed predominant localization of the hole and particle NTOs of the S_1_ and T*_n_* (*n* = 1 and 2) states on the MR‐core (Figure S21 and Table S3, Supporting Information), confirming the preservation of SRCT character, i.e., LE at the MR‐core, in the electronic transition processes of **1** and **2**, as expected from the distribution of FMOs in the S_0_ state.

**Figure 5:**
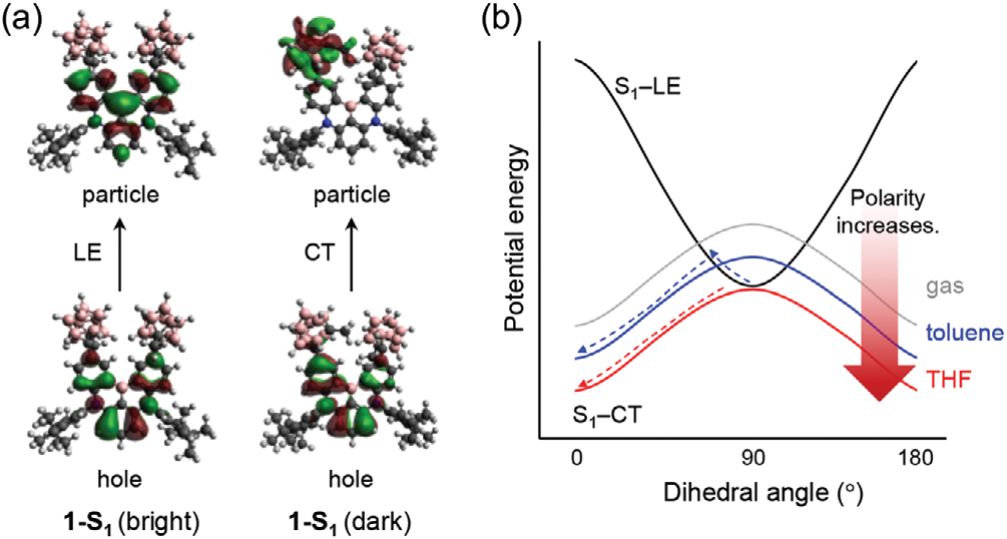
a) NTOs (isovalue = 0.02 e Å^−3^) for bright and dark S_1_ states of **1**. b) Schematic diagram for the potential energy surfaces of bright and dark S_1_ states of **1**, depending on surrounding medium.

To examine the spectral quenching in a polar solvent medium, additional TDDFT calculations were performed for **1**, which exhibited superior TADF performance compared to **2**. In addition to the bright S_1_ (S_1_‐LE) state geometrically similar to the S_0_, we identified a dark S_1_ (S_1_‐CT) state dependent on the C_CB_−C_CB_ bond length and the dihedral angle *ψ* (see Table S4 (Supporting Information) for their geometries). Their transition characters were elucidated by NTO analysis (**Figure** **5**a; Table S5, Supporting Information). The negligible oscillator strength (*f*) of S_1_‐CT indicates that the spectral quenching for **1** is closely related to a non‐emissive relaxation process from S_1_‐CT (Table S4, Supporting Information). The schematic potential energy surfaces (PESs), depending on *ψ* for one of the two carboranes, constructed using TDDFT results, reveal minima for the bright and dark S_1_ states at ≈90° and ≈0° (≈180°), respectively (Figure 5b; Figure S23, Supporting Information). The spectral quenching can thus be understood with structural changes, i.e., the dihedral rotation of *o*‐carborane accompanied with C_CB_−C_CB_ bond elongation, from S_1_‐LE to S_1_‐CT (Figure S24, Supporting Information). The extent of the relative stability of S_1_‐LE with respect to S_1_‐CT at ≈90° decreases gradually as the polarity of the surrounding medium increases. In particular, the minimum energy in the PES of S_1_‐LE is almost comparable to the maximum energy in the PES of S_1_‐CT in the most polar THF medium. Consequently, the relative distribution of PES for S_1_‐CT to that for S_1_‐LE indicates that the energy barrier from S_1_‐LE to S_1_‐CT states can be interpreted as diminishing with the increasing polarity of the surrounding medium. Because the rotational PES was constructed via a series of single‐point calculations by only changing the dihedral angle *ψ* from the optimized structure, it cannot precisely describe the reaction barrier required for the geometric change from S_1_‐LE to S_1_‐CT. Especially, the energy barrier due to C_CB_−C_CB_ bond elongation was not addressed. Nevertheless, the experimental spectral quenching can be qualitatively rationalized with the gradual change of the PES of S_1_‐CT with respect to S_1_‐LE depending on the environmental polarity at the level of theory employed in PES evaluation.

### 2.4 Electroluminescent Properties

**Figure 6:**
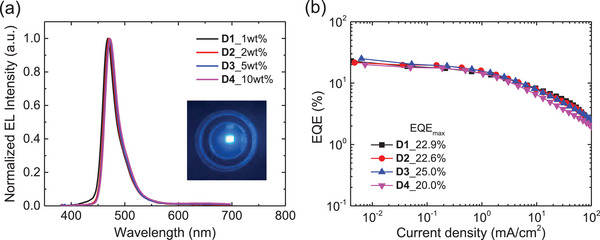
a) EL spectra of TADF‐OLEDs (**D1**−**D4**) based on emitter **1** at different doping concentrations in the emitting layer and photograph of the working device. b) External quantum efficiency−current density (EQE−*J*) characteristics of **D1**−**D4**.

Finally, the *o*‐carboranyl MR‐TADF emitters were applied to blue‐emitting OLEDs. We selected **1** as the emitter due to its excellent emissive properties. Prior to OLED fabrication, the photophysical properties of **1** and **3** were evaluated in a mixed host of 9‐(3‐(triphenylsilyl)phenyl)−9*H*‐3,9′‐bicarbazole (SiCzCz): 9,9′‐(6‐(3‐(triphenylsilyl)phenyl)−1,3,5‐triazine‐2,4‐diyl)bis(9*H*‐carbazole) (SiTrzCz2) (Figure S25 and Table S6, Supporting Information).^[^ ^12^ ^]^ The 5 wt.% doped thin films of **1** and **3** in the host matrix exhibited narrowband emissions with FWHMs of 26 and 27 nm and peak wavelengths at 472 and 459 nm, respectively. Notably, the thin film of **1** showed a high PLQY of ≈93%, comparable to that of **3** (92%). The shorter delayed fluorescence lifetime (*τ*_d_) of **1** (69.1 µs) compared to that of **3** (92.6 µs) is mainly attributable to the smaller S_1_−T_1_ gap (Δ*E*_ST_) of **1** relative to that of **3** (0.14 vs 0.16 eV), as similarly observed in a PMMA film (Figure 3d; Figure S25b, Supporting Information). These results indicate the excellent MR‐TADF properties of **1** in the host film. Next, the OLEDs with the following structure were constructed (Figure S27, Supporting Information): glass/indium‐tin‐oxide (ITO, 70 nm)/MoO_3_ (1 nm)/1,1‐bis[(di‐4‐tolylamino)phenyl]cyclohexane (TAPC, 30 nm)/tris(4‐carbazoyl‐9‐ylphenyl)amine (TCTA, 10 nm)/SiCzCz (10 nm)/SiCzCz:SiTrzCz2:emitter (70:30:x wt.%, 25 nm)/SiTrzCz2 (5 nm)/1,3,5‐tri(*m*‐pyridin‐3‐ylphenyl)benzene (TmPyPB, 40 nm)/LiF (1 nm)/Al (130 nm). As depicted in **Figure** **6**, **D1**−**D4** devices with a wide range of doping concentrations of emitter **1** (1, 2, 5, and 10 wt.%) exhibited blue electroluminescence (EL) with emission peaks at 468−472 nm. In particular, the narrow FWHM of 27−28 nm was maintained in the given doping range. These EL spectral features are consistent with the PL spectrum of the host film of **1** and can be partly attributed to the steric shielding of the MR‐core by the cage. Remarkably, all devices exhibited high EQE_max_ above 20%, owing to the high PLQY and TADF property of **1** (**Table** **2**). Among them, **D3** showed the highest EQE_max_ of 25.0%, which ranks as the highest among reported OLEDs utilizing an *o*‐carboranyl luminophore as the emitter.^[^ ^1^, ^13^ ^]^ The efficiency is comparable to that of the control device (**R3**) employing 5 wt.% emitter **3** with an EQE_max_ of 25.1%. However, the efficiency roll‐off of **D3** at high current density is smaller than that of the control device. This result demonstrates that the incorporation of *o*‐carborane into the B,N‐doped MR‐emitting core allows us to effectively tune the emission color and reduce intermolecular interactions between the MR‐cores, leading to maintaining the blue color purity with high EQE even at high doping concentrations.

**Table 2:** Device performances of the TADF‐OLEDs.

Device^a)^	Emitter [wt.%]	*λ*_EL_ [nm]	FWHM [nm]^b)^	CIE (x, y)^c)^	*V*_on_ [V]^d)^	EQE [%]^e)^	PE [lm W^−1^]^f)^	CE [cd A^−1^]^g)^
**D1**	2CB‐BuDABNA (1)	468	28	(0.133, 0.154)	3.0	22.9/13.6	25.7/12.0	22.1/14.8
**D2**	2CB‐BuDABNA (2)	470	27	(0.128, 0.178)	3.0	22.6/15.6	28.1/14.5	24.1/18.0
**D3**	2CB‐BuDABNA (5)	472	27	(0.128, 0.187)	3.0	25.0/16.9	31.1/17.6	26.8/20.1
**D4**	2CB‐BuDABNA (10)	472	28	(0.133, 0.207)	3.0	20.0/16.1	19.9/17.7	22.4/20.4
**R3**	BuDABNA (5)	460	27	(0.137, 0.093)	3.0	25.1/12.7	22.4/7.5	19.3/10.0

^a)^ ITO (70 nm)/MoO_3_ (1 nm)/TAPC (30 nm)/TCTA (10 nm)/SiCzCz (10 nm)/SiCzCz:SiTrzCz2:emitter (70:30:x wt.%, 25 nm)/SiTrzCz2 (5 nm)/TmPyPB (40 nm)/LiF (1 nm)/Al (130 nm);

^b)^ Full width at half maximum;

^c)^ Color coordinates (CIE 1931) at 100 cd m^−2^ from the normal direction;

^d)^ Applied voltage at a luminance of 1 cd m^−2^;

^e)^ External quantum efficiency: maximum, then value at 100 cd m^−2^;

^f)^ Power efficiency: maximum, then value at 100 cd m^−2^;

^g)^ Current efficiency: maximum, then value at 100 cd m^−2^_._

## 3 Conclusion

In conclusion, we have demonstrated that the local emission characteristics of the parent π‐luminophores can be preserved while being tuned through *o*‐carborane substitution. 2CB‐BuDABNA (**1**) and 3CB‐BuDABNA (**2**), wherein methyl‐*o*‐carborane moieties were introduced at the periphery of the MR‐emitting core, were prepared. Both compounds exhibited intense MR‐TADF in toluene and rigid states, with significant redshifts of their emission peaks compared to that of the parent compound BuDABNA (**3**). Utilizing **1** as the emitter, we achieved highly efficient blue OLEDs with an EQE_max_ of 25%. This efficiency stands as unprecedented for OLEDs based on *o*‐carboranyl luminophores as emitters. The findings of this study indicate that *o*‐carborane can serve as a steric and electronic auxiliary for MR‐TADF emitters, holding promise for the development of a novel class of *o*‐carboranyl TADF materials.

## 4 Experimental Section

### Synthesis of **1**

To a solution of **1b** (0.30 g, 0.30 mmol) in *t*‐butylbenzene (20 mL) was added dropwise *t*‐BuLi (1.6 m in pentane, 0.57 mL, 0.91 mmol) at −30 °C. After stirring at 60 °C for 2 h, pentane was removed in *vacuo*. Boron tribromide (0.09 mL, 0.91 mmol) was added slowly at −30 °C, and the mixture was stirred at 60 °C for 1 h. *N*,*N*‐Diisopropylethylamine (DIPEA, 0.16 mL, 0.91 mmol) was added at 0 °C, and the reaction mixture was stirred at 120 °C for 12 h. After cooling down to room temperature, an aqueous solution of NaOAc was added, and the mixture was extracted with ethyl acetate (3 × 30 mL). The combined organic layer was dried over MgSO_4_, filtered, and washed several times with ethyl acetate. The solution was concentrated under reduced pressure and filtered through a silica gel (eluent: dichloromethane/*n*‐hexane = 1:5) to give a yellow solid. The product was further purified by crystallization, affording the title compound as a bright yellow solid (Yield: 0.09 g, 30%). ^1^H NMR (400 MHz, CD_2_Cl_2_, δ): 9.11 (d, *J* = 2.4 Hz, 2H), 7.69 (dt, *J* = 5.0, 2.8 Hz, 4H), 7.35 (t, *J* = 8.3 Hz, 1H), 7.17 (d, *J* = 1.7 Hz, 4H), 6.81 (d, *J* = 9.2 Hz, 2H), 6.26 (d, *J* = 8.3 Hz, 2H), 1.80 (s, 6H), 1.37 (s, 46H); ^13^C NMR (100 MHz, CD_2_Cl_2_, δ): 154.91, 149.09, 146.89, 141.12, 138.47, 133.37, 124.02, 123.24, 122.45, 118.12, 106.85, 84.22, 78.48, 35.51, 31.55, 23.49; ^11^B NMR (128 MHz, CD_2_Cl_2_, δ): 30.0 (1B), −4.1 (4B), −10.1 (16B); Anal. calcd for C_52_H_77_B_21_N_2_: C 65.25, H 8.11, N, 2.93; found: C 65.06, H 7.92, N 2.81; *T*_d5_ = 400 °C.

### Synthesis of **2**

This compound was prepared in a manner analogous to the synthesis of **1** using **2b** (0.35 g, 0.35 mmol), affording the title compound as a bright yellow solid (Yield: 0.05 g, 15%). ^1^H NMR (400 MHz, CD_2_Cl_2_, δ): 8.87 (d, *J* = 8.2 Hz, 2H), 7.74 (t, *J* = 1.6 Hz, 2H), 7.48 (dd, *J* = 8.2, 1.7 Hz, 2H), 7.41 (t, *J* = 8.3 Hz, 1H), 7.18 (d, *J* = 1.7 Hz, 4H), 7.01 (d, *J* = 1.6 Hz, 2H), 6.39 (d, *J* = 8.3 Hz, 2H), 1.66 (s, 6H), 1.39 (s, 36H); ^13^C NMR (100 MHz, CD_2_Cl_2_, δ): 155.30, 148.09, 147.12, 141.21, 135.50, 133.43, 133.07, 124.09, 123.24, 121.91, 121.18, 106.27, 82.81, 77.94, 35.58, 31.60, 23.50; ^11^B NMR (128 MHz, CD_2_Cl_2_, δ): 41.6 (1B), −4.9 (4B), −10.2 (16B). Anal. calcd for C_52_H_77_B_21_N_2_: C 65.25, H 8.11, N 2.93; found: C 65.04, H 8.28, N 2.52; *T*_d5_ = 382 °C.

### Device Fabrication and Characterization

Pre‐cleaned ITO patterned glass substrates with acetone, and isopropyl alcohol were loaded into a thermal evaporator (located at the 3D Convergence Center of Inha University). Then, organic layers and metal electrodes were deposited on the substrates under a base pressure of <5 × 10^−7^ torr. During the process, shadow masks were used to define the active area of 2 × 2 mm^2^. The deposition rate was 1 Å s^−1^ for the organic layers, 0.1 Å s^−1^ for LiF, and 3 Å s^−1^ for Al. The thicknesses of the layers were controlled by monitoring crystal sensors. The final stage of the process was encapsulation using a UV‐resin and glass lid. It was conducted inside a glove box filled with N_2_ gas. The TADF‐OLEDs were analyzed using a Keithley 2400 source meter and spectrophotometer (Photo Research 670) for the optoelectrical characteristics.

[CCDC 2 295 543 for **1** and 2 295 544 for **2** contain the supplementary crystallographic data for this paper. These data can be obtained free of charge from The Cambridge Crystallographic Data Centre via www.ccdc.cam.ac.uk/data_request/cif.]

## Conflict of Interest

The authors declare no conflict of interest.

## Supporting information

Supporting Information

## Data Availability

The data that support the findings of this study are available from the corresponding author upon reasonable request.
