# Supplementary material for: Ortho‐Carborane Decorated Multi‐Resonance TADF Emitters: Preserving Local Excited State and High Efficiency in OLEDs
Source: Adv Sci (Weinh). 2024 Jan 17;11(11):2309016. doi: 10.1002/advs.202309016 (PMC10953543; doi:10.1002/advs.202309016)
Supplement: Supplementary file 1 — Supporting Information [file ADVS-11-2309016-s001.pdf]

ADVANCED SCIENCE
Open Access

Supporting Information

for *Adv. Sci.*, DOI 10.1002/advs.202309016

Ortho-Carborane Decorated Multi-Resonance TADF Emitters: Preserving Local Excited State and High Efficiency in OLEDs

Taehwan Lee, Jee-Hun Jang, Nhi Ngoc Tuyet Nguyen, Jaehoon Jung, Jeong-Hwan Lee* and Min Hyung Lee**

Supporting Information

Ortho-Carborane Decorated Multi-Resonance TADF Emitters: Preserving Local Excited State and High Efficiency in OLEDs

Taehwan Lee, Jee-Hun Jang, Nhi Ngoc Tuyet Nguyen, Jaehoon Jung, Jeong-Hwan Lee,* and Min Hyung Lee**

Experimental

General Considerations

All operations were performed under an inert nitrogen atmosphere using standard Schlenk and glove box techniques. Anhydrous grade solvents (Aldrich) were dried over activated molecular sieves (5Å). Spectrophotometric-grade toluene and tetrahydrofuran (THF) were used as received from Aldrich and Alfa, respectively. Commercial reagents were used without further purification after purchase. 1-(4-Bromophenyl)-2-methyl-1,2-*closo*-carborane (**1a**),[1] 1-(3-bromophenyl)-2-methly-1.2-*closo*-carborane (**2a**),[1] and 2-chloro-N^1,N^3-bis(3,5-di-*tert*-butylphenyl)benzene-1,3-diamine[2] were synthesized according to the modified literature procedures. Deuterated solvents from Eurisotop were used. NMR spectra were recorded on a Bruker AVANCE III HD 400 (400.13 MHz for ^{1}H, 100.61 MHz for ^{13}C, 128.38 MHz for ^{11}B) spectrometer at ambient temperature. Chemical shifts are given in ppm, and are referenced against external Me_4Si (^{1}H, ^{13}C) and $BF_3 \cdot OEt_2$ (^{11}B). Mass spectra were obtained using a JEOL JMS700 high-resolution EI-mass spectrometer (HR EI-MS) at the Korea Basic Science Institute, Daegu, Korea. Elemental analyses were performed on a Flash 2000 elemental analyzer (Thermo Scientific). Thermogravimetric analysis (TGA) was performed with a TA Instruments Q50

under an N_2 atmosphere at a heating rate of 10 °C/min. Cyclic voltammetry experiments were carried out using a CHI600E system.

Synthesis

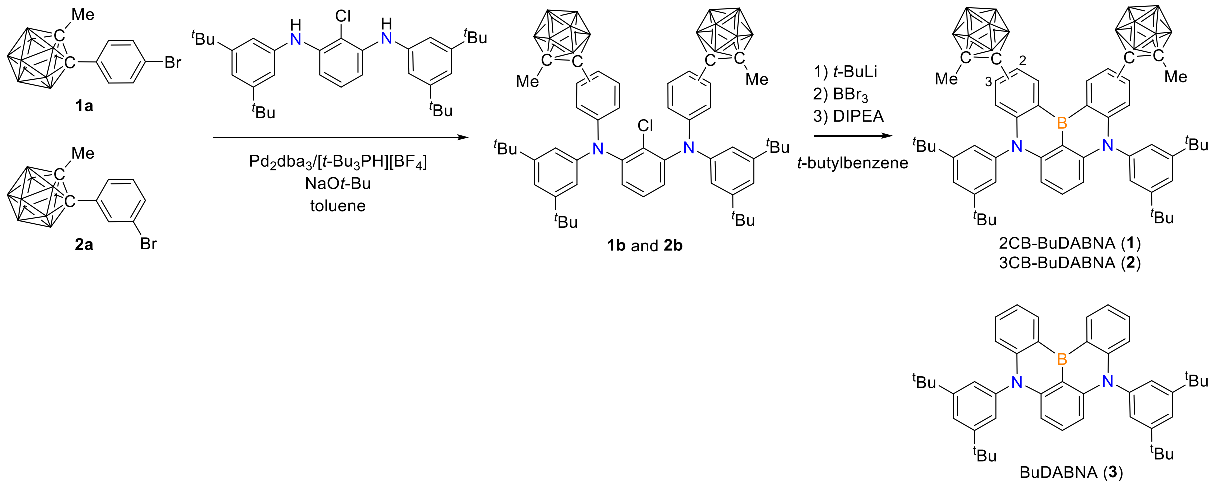

Scheme S1. Synthesis of 2CB-BuDABNA (**1**) and 3CB-BuDABNA (**2**).

Synthesis of 1b. A mixture of **1a** (0.67 g, 2.12 mmol), 2-chloro-N^1,N^3-bis(3,5-di-*tert*-butylphenyl)benzene-1,3-diamine (0.50 g, 0.96 mmol), $Pd_2(dba)_3$ (26 mg, 0.03 mmol), ${}^t Bu_3PHBF_4$ (17 mg, 0.06 mmol), and NaO*t*Bu (0.28 g, 2.89 mmol) in dry toluene (20 mL) were stirred overnight at 90 °C. After cooling down to room temperature, the mixture was filtered through a Celite pad, washed with dichloromethane and the filtrate was concentrated under reduced pressure. The crude product was purified by column chromatography on silica gel using dichloromethane/*n*-hexane (1:10, v/v) as eluent to give **1b** as a white solid (Yield: 0.65 g, 69%). ^{1}H NMR ($CDCl_3$): δ 7.41–7.32 (m, 5H), 7.28 (s, 2H), 7.19 (t, J = 1.6 Hz, 2H), 6.98 (t, J = 10.5 Hz, 4H), 6.69 (d, J = 9.0 Hz, 4H), 1.67 (s, 6H), 1.27 (s, 36H). ^{13}C NMR ($CDCl_3$): δ 152.34, 149.32, 145.28, 144.30, 133.69, 131.96, 129.90, 129.21, 121.78, 119.24, 118.97, 117.21, 83.21, 35.09, 31.52, 23.12. ^{11}B NMR ($CDCl_3$): δ −4.7 (4B), −10.0 (16B). HRMS (FAB): m/z [M]+ Calcd for $C_{52}H_{79}ClN_2B_{20}$: 986.7793; Found: 986.7800.

Synthesis of 2b. This compound was prepared in a manner analogous to the synthesis of **1b** using **2a** (0.67 g, 2.12 mmol), affording the title compound as a white solid (Yield: 1.22 g, 93%). ^{1}H NMR ($CDCl_3$): δ 7.32 (dd, J = 8.5, 7.3 Hz, 1H), 7.22 (s, 1H), 7.21–7.17 (m, 4H), 7.16 (s, 1H), 7.13 (d, J = 8.8 Hz, 4H), 6.88 (d, J = 1.6 Hz, 4H), 6.83 (d, J = 8.0 Hz, 2H), 1.62 (s, 6H), 1.25 (s, 36H). ^{13}C NMR ($CDCl_3$): δ 152.19, 147.71, 145.74, 145.53, 132.66, 131.66, 129.62,

129.30, 128.89, 123.47, 122.64, 121.58, 118.12, 117.86, 82.37, 35.06, 31.56, 23.20. ^{11}B NMR ($CDCl_3$): *δ* −4.0 (4B), −9.7 (16B). HRMS (FAB): m/z [M]+ Calcd for $C_{52}H_{79}ClN_2B_{20}$: 986.7793; Found: 986.7795.

Synthesis of 1. To a solution of **1b** (0.30 g, 0.30 mmol) in *t*-butylbenzene (20 mL) was added dropwise *t*-BuLi (1.6 M in pentane, 0.57 mL, 0.91 mmol) at −30 °C. After stirring at 60 °C for 2 h, pentane was removed in *vacuo.* Boron tribromide (0.09 mL, 0.91 mmol) was added slowly at −30 °C, and the mixture was stirred at 60 °C for 1 h. *N,N*-Diisopropylethylamine (DIPEA, 0.16 mL, 0.91 mmol) was added at 0 °C, and the reaction mixture was stirred at 120 °C for 12 h. After cooling down to room temperature, an aqueous solution of NaOAc was added, and the mixture was extracted with ethyl acetate (3 × 30 mL). The combined organic layer was dried over $MgSO_4$, filtered, and washed several times with ethyl acetate. The solution was concentrated under reduced pressure and filtered through a silica gel (eluent: dichloromethane/*n*-hexane = 1:5) to give a yellow solid. The product was further purified by crystallization, affording the title compound as a bright yellow solid (Yield: 0.09 g, 30%). ^{1}H NMR (CD_2Cl_2): *δ* 9.11 (d, *J* = 2.4 Hz, 2H), 7.69 (dt, *J* = 5.0, 2.8 Hz, 4H), 7.35 (t, *J* = 8.3 Hz, 1H), 7.17 (d, *J* = 1.7 Hz, 4H), 6.81 (d, *J* = 9.2 Hz, 2H), 6.26 (d, *J* = 8.3 Hz, 2H), 1.80 (s, 6H), 1.37 (s, 46H). ^{13}C NMR (CD_2Cl_2): *δ* δ 154.91, 149.09, 146.89, 141.12, 138.47, 133.37, 124.02, 123.24, 122.45, 118.12, 106.85, 84.22, 78.48, 35.51, 31.55, 23.49. ^{11}B NMR (CD_2Cl_2): *δ* 30.0 (1B), −4.1 (4B), −10.1 (16B). Anal. Calcd (%) for $C_{52}H_{77}B_{21}N_2$: C, 65.25; H, 8.11; N, 2.93. Found: C, 65.06; H, 7.92; N, 2.81. T_{d5} = 400 °C.

Synthesis of 2. This compound was prepared in a manner analogous to the synthesis of **1** using **2b** (0.35 g, 0.35 mmol), affording the title compound as a bright yellow solid (Yield: 0.05 g, 15%). ^{1}H NMR (CD_2Cl_2): *δ* 8.87 (d, *J* = 8.2 Hz, 2H), 7.74 (t, *J* = 1.6 Hz, 2H), 7.48 (dd, *J* = 8.2, 1.7 Hz, 2H), 7.41 (t, *J* = 8.3 Hz, 1H), 7.18 (d, *J* = 1.7 Hz, 4H), 7.01 (d, *J* = 1.6 Hz, 2H), 6.39 (d, *J* = 8.3 Hz, 2H), 1.66 (s, 6H), 1.39 (s, 36H). ^{13}C NMR (CD_2Cl_2): *δ* 155.30, 148.09, 147.12, 141.21, 135.50, 133.43, 133.07, 124.09, 123.24, 121.91, 121.18, 106.27, 82.81, 77.94, 35.58, 31.60, 23.50. ^{11}B NMR (CD_2Cl_2): *δ* 41.6 (1B), −4.9 (4B), −10.2 (16B). Anal. Calcd (%) for $C_{52}H_{77}B_{21}N_2$: C, 65.25; H, 8.11; N, 2.93. Found: C, 65.04; H, 8.28; N, 2.52. T_{d5} = 382 °C.

Synthesis of 3. This compound was prepared in a manner analogous to the synthesis of **1** using 2-chloro-N^1,N^3-bis(3,5-di-*tert*-butylphenyl)benzene-1,3-diamine (0.35 g, 0.35 mmol), affording the title compound as a bright yellow solid (Yield: 0.11 g, 32%). ^{1}H NMR (CD_2Cl_2):

δ 8.93 (dd, J = 7.7, 1.3 Hz, 2H), 7.66 (t, J = 1.7 Hz, 2H), 7.44 (ddd, J = 8.6, 7.0, 1.6 Hz, 2H), 7.26 (dt, J = 5.8, 4.5 Hz, 3H), 6.79 (d, J = 8.6 Hz, 2H), 6.15 (d, J = 8.3 Hz, 2H), 1.37 (s, 36H). ^{13}C NMR (CD_2Cl_2): δ 154.58, 148.23 , 147.37 , 141.98 , 135.23 , 132.46 , 131.32 , 124.58 , 122.62 , 120.03 , 117.67 , 105.40 , 35.50 , 31.62. ^{11}B NMR (CD_2Cl_2): δ 40.91 (1B). Anal. Calcd (%) for $C_{46}H_{53}BN_2$: C, 85.69; H, 8.29; N, 4.34. Found: C, 85.43; H, 8.35; N, 4.13. T_{d5} = 303 °C.

X-ray Crystallography. Crystal data for **1** and **2** were collected on a Bruker SMART APEX II ULTRA diffractometer equipped with graphite monochromated Mo Kα radiation (λ = 0.71073 Å) generated by a rotating anode at 123 K. Data collection, data reduction, and semi-empirical absorption correction were carried out using the software package of APEX2. The crystal structures were solved and refined using the SHELXL package. Since the lattice solvent molecules are highly disordered, the contribution of solvent electron density was removed by the SQUEEZE routine in PLATON.[3] Full details of the structure determinations have been deposited as cifs with the Cambridge Crystallographic Data Collection under CCDC deposition numbers 2295543 (**1**) and 2295544 (**2**). These data can be obtained free of charge via www.ccdc.cam.ac.uk/data_request/cif.

Cyclic Voltammetry. Cyclic voltammetry measurements were carried out in CH_2Cl_2 (1×10^{-3} M) for oxidation and in DMF/THF (9:1, v/v, 1.5×10^{-3} M) for reduction, with a three-electrode cell configuration consisting of platinum working and counter electrodes and a Ag/$AgNO_3$ (0.01 M in CH_3CN) reference electrode at room temperature. Tetra-*n*-butylammonium hexafluorophosphate (0.1 M) was used as the supporting electrolyte. The redox potentials were recorded at a scan rate of 100 mV/s and are reported with reference to the ferrocene/ferrocenium (Fc/Fc^+) redox couple. The HOMO energy levels were determined from the electrochemical oxidation ($E_{1/2}$) peaks of cyclic voltammograms while the LUMO energy levels were estimated from the optical bandgap (E_g) and the HOMO levels.

Photophysical Measurements. UV/Vis absorption and photoluminescence (PL) spectra were recorded on a UV-2600 (Shimadzu) and an Edinburgh Instruments FLS1000 spectrophotometer, respectively. Dilute sample solutions (typically 20 μM in oxygen-free solvent) were prepared in a glove box at ambient conditions. Photoluminescence quantum yields (PLQYs) all the samples were measured on an absolute PL quantum yield spectrophotometer (Quantaurus-QY C11347-11, Hamamatsu Photonics) equipped with a 3.3-inch integrating sphere. Transient PL decays were measured on an FLS1000 spectrophotometer with an excitation light source of

EPL-375 laser for prompt fluorescence and VPL-375 laser (for host films) or Xe flashlamp (for PMMA films) for delayed fluorescence. The photophysical analysis done by using FLS1000 and Quantaurus-QY C11347-11 spectrophotometers was conducted at total-period analysis center for Ulsan chemical industry of KBSI.

Computation Details. The regioselective substitution of 2-methyl-*o*-carborane (CB) into the multi-resonance induced thermally activated delayed fluorescence (MR-TADF) core was successfully demonstrated in our experiment (**Scheme 1** in the main text). Employing the CB moiety at the 2- and 3-positions of the MR-emitting core leads to the formation of 2CB-BuDABNA (**1**) and 3CB-BuDABNA (**2**), respectively. The CB moiety is an inductively electron-withdrawing and conjugation-extending substituent, and thus is expected to mainly affect the HOMO and LUMO for **1** and **2**, respectively, due to their alternative spatial distributions in the MR-emitting skeleton.[4] However, our experimental measurements indicated that both **1** and **2** exhibited red-shifted absorption and emission spectra compared with the reference compound, BuDABNA (**3**), which lacks CB moieties. In order to elucidate the detailed electronic and photophysical properties of compounds **1** and **2**, we conducted a theoretical study based on density functional theory (DFT) and time-dependent DFT (TDDFT) using the PBE0 hybrid functional[5] and the def2-SVP basis set[6] implemented in the GAUSSIAN 16 software package.[7] The Tamm-Dancoff approximation (TDA)[8] was employed for TDDFT calculations due to its improved performance in estimating ΔE_{ST} values.[9] The ground (S_0) states of compounds were optimized using DFT calculations, and their lowest singlet (S_1) and two triplet (T_1 and T_2) excited states were optimized using TDDFT calculations. The optimized S_0 geometry was used as an initial structure for optimizing the geometries of the excited states, S_1, T_1, and T_2 (**Table S2**). All the optimized structures were confirmed to be local minima through frequency calculations. The polarizable continuum model using the integral equation formalism (IEFPCM) within a self-consistent reaction field (SCRF) approximation was utilized to address the influence of the solvent medium, i.e., toluene, on the geometric and electronic structures.[10] Notably, our DFT calculations indicated that compound **2** is slightly more stable by 0.29 kcal/mol when the two methyl groups of the CB moieties are located on opposite sides with respect to the plane of the B,N-core, compared to when they are located on the same side. Thus, the X-ray crystal structure of **2** (**Figure 2** in the main text) is expected to be formed due to intermolecular packing in the molecular crystal. The spatial distribution of their frontier molecular orbitals (FMOs), i.e., HOMO and LUMO, is predominantly localized at the B,N core of the DABNA backbone in an alternating manner (**Figure S18** and **Table S2**). The CB moiety

is therefore expected to play a role in finely tuning the photophysical properties of MR-TADF compounds while preserving the inherent local transition characteristics, such as short-range charge transfer (SRCT) of the MR-emitting core.[11] The larger contributions of the CB moiety to LUMO (3.5% in **1** and 10.9% in **2**) than to HOMO (2.5% in **1** and 0.6% in **2**) may lead to more efficient stabilization of LUMO (by 0.52 eV in **1** and 0.72 eV in **2** from the LUMO energy of **3**) compared with HOMO (by 0.46 eV in **1** and 0.41 eV in **2** from the HOMO energy of **3**). Thus, their reduced energy gap between HOMO and LUMO (E_g) by 0.06 and 0.31 eV for **1** and **2**, respectively, compared to the E_g of **3**, can lead to redshifts in the absorption and emission spectra of **1** and **2**. The reduced E_g and computationally obtained absorption and emission wavelengths are in qualitative agreement with our experimental observations. The larger stabilization of LUMO in **2** can be readily understood with the CB substituted at the LUMO-dominant 3-position (see **Table S2**). However, the LUMO of **1** is unexpectedly more stabilized than its HOMO, despite CB being introduced at the HOMO-dominant 2-position. To unveil the formation of FMOs resulting from electronic coupling between the CB and MR-emitting core, we conducted fragment orbital (FO) analysis using the AOMix software package.[12] As expected for **2**, the α-LUFO (β-LUFO+2) of the MR-core is strongly coupled with the β-LUFO+5 (α-LUFO+3) of the CB moieties, forming the LUMO of **2**, while its HOMO is exclusively derived from the α-HOFO (β-HOFO) of the MR-core (**Figure S19**). For compound **1**, electronic coupling was not observed for the α-electronic states (α-LUFO and α-HOFO) of the MR-core. However, recognizable electronic couplings leading to the formation of the LUMO of **1** were obtained between the β-LUFO and β-LUFO+2 of the MR-core and the α-LUFO+3 and α-LUFO+7 of the CB moieties, in addition to strong coupling between the β-HOFO of the MR-core and the α-LUFO+3 of the CB to form the HOMO of **1** (**Figure S20**). Particularly, the feasibility of a σ-orbital contribution to the LUMO of **1**, as identified from the spatial distribution of β-LUFO of the MR-core (**Figure S20**), which was not observed in the electronic couplings in **2**, would be considered to extraordinarily stabilize the LUMO of **1** compared to its HOMO, resulting in the reduction of the E_g of **1**.

A natural transition orbital (NTO)[13] analysis was performed to clarify the preservation of SRCT characteristics, i.e., local emission (LE) in the MR-core, in the electronic transition processes of **1** and **2** as MR-TADF compounds. All NTO analyses were carried out using the Multiwfn program.[14] The NTO analysis revealed that the hole and particle NTOs of the excited S_1 and T_n (n = 1 and 2) states were predominantly localized on the DABNA core for both **1** and **2**, similar to the reference compound **3** (**Figure S21**). The contributions of the CB moiety to the NTOs at the S_1 and T_1 states (**Table S3**) were comparable to those to the FMOs at the S_0 state, as

mentioned earlier (**Table S2**). Notably, compound **2** exhibited exceptionally large contributions of the CB moiety to the particle NTOs, with values of 9.9% and 7.4% for its S_1 and T_1 states, respectively. The considerable participation of the CB moiety in the transition processes of **2** may cause not only the larger redshift in emission wavelength of **2** but also its slightly lower PLQY compared to **1**. The spin-orbit coupling matrix elements (SOCME) between the S_1 and T_n (n = 1 and 2) states were further calculated to understand their RISC processes (**Figure S21**). The SOCME values were computed using the quasi-degenerate perturbation theory,[15] implemented in the ORCA software package,[16] and the same functional and basis set as used in the DFT and TDDFT calculations. All the compounds exhibited similar numerical values of SOCME between the S_1 and T_n (n = 1 and 2) states, with negligible S_1–T_1 ($\langle S_1|\hat{H}_{SOC}|T_1\rangle$ = ~0.01 to ~0.02 cm^{-1}) or small S_1–T_2 ($\langle S_1|\hat{H}_{SOC}|T_2\rangle$ = ~0.12 to 0.16 cm^{-1}) SOCME values. The small magnitude of SOCME values suggests that the RISC rate would be primarily governed by the ΔE_{ST}. Note that the experimentally measured ΔE_{ST} values and the corrected S_1 energies along with experimental ΔE_{ST} are provided in **Figure S21**, as TDDFT calculations are known to overestimate ΔE_{ST} for MR-TADF molecules due to inaccuracies in estimating the Coulomb interaction, particularly for the S_1 state.[17]

Lastly, more detailed TDDFT calculations were performed for 2CB-BuDABNA (**1**) to examine the dependence of its transition process from the S_1 to S_0 state on the geometry and further to understand its spectral quenching in a polar solvent medium. Compound **1** displayed superior TADF performance, including a high PLQY and a narrow FWHM, compared to **2** (**Table 1** in the main text). The nature of quenching in **2** is anticipated to be similar to that in **1**. For the toluene medium, the geometry optimization for the S_0 state only resulted in a relative orientation where both methyl groups of the CB moieties are perpendicular to the plane of the B,N-core (ψ = 93.0°), as mentioned earlier. However, besides the bright S_1 local minimum (**1-S₁-LE**) similar to S_0, we identified a dark S_1 local minimum structure (**1-S₁-CT**) dependent on the C_{CB}–C_{CB} bond length, $d(C_{CB}–C_{CB})$, and the dihedral angle ψ between the CB and B,N-core for one of the two CB moieties (**Table S4**). While the C_{CB}–C_{CB} bonds in both CB moieties were maintained at 1.69 Å in **1-S₁-LE** compared to its S_0 geometry, the $d(C_{CB}–C_{CB})$ in one of the two CB moieties in **1-S₁-CT** substantially elongated to 2.45 Å. A significant geometric difference was also found in the dihedral orientation of the CB with respect to the plane of the B,N-core. The methyl group of the CB, for which the C_{CB}–C_{CB} bond is elongated, became positioned parallel to the plane of the B,N-core in **1-S₁-CT** (ψ = 4.4°), whereas its orientation in **1-S₁-LE** (ψ = 82.7°) remained perpendicular, similar to S_0. The transition characters were revealed through the NTO analysis, distinguishing between local emission (LE) typically described as SRCT in

MR-TADF compounds and MR-core-to-CB charge transfer (CT) for **1-S_1-LE** and **1-S_1-CT**, respectively (**Figure 5a** and **Table S5**).[18] Their oscillator strengths were computed to be 0.2679 and 0.0018, respectively. Therefore, the negligible oscillator strength (*f*) of **1-S_1-CT** indicates that the spectral quenching for **1** can be explained by a non-emissive relaxation process from S_1 to S_0 at the geometry of **1-S_1-CT**. In addition to the TDDFT computation in toluene (ε = 2.3741) medium, we extended our computational study to the gas phase and the more polar THF (ε = 7.4257) medium to understand the dependence of spectral activity on the polarity of the medium. Both local minimum S_1 states obtained for toluene medium were also found for the gas phase and THF medium. **1-S_1-CT** not only exhibited higher stability compared to **1-S_1-LE** in all surrounding media, but also the extent of its relative stability compared to **1-S_1-LE** increased as the polarity of the surrounding medium increased: in the gas phase, toluene, and THF media, **1-S_1-CT** was more stable than **1-S_1-LE** by 5.31, 6.01, and 6.44 kcal/mol, respectively. Thus, our TDDFT results suggest that the enhanced stability of the non-emissive state (**1-S_1-CT**) in a more polar medium is responsible for the spectral quenching in a polar solvent medium. To gain deeper insight into the dependence of emission on structural variation, the potential energy surfaces (PESs) for the S_1 state of **1** were approximately investigated using a series of single-point calculations that varied the dihedral angle (ψ) of the C_{CB}–C_{CB} bond with respect to the plane of the B,N-core. The PESs for bright **1-S_1-LE** and dark **1-S_1-CT** were constructed by increasing the dihedral angle from 0° to 180° with 10° increments (**Figure S22**). As expected, the PESs, depending on ψ, showed minima at ~90° and ~0° (~180°) for **1-S_1-LE** and **1-S_1-CT**, respectively (**Figure S23**). The oscillator strength, an indicator of the intensity of emission, of **1-S_1-CT** rapidly decreased as ψ approached 0° (180°), while that of **1-S_1-LE** did not depend on ψ (**Figure S24**). Therefore, the spectral quenching can be understood with structural changes, i.e., the dihedral rotation of C_{CB}–C_{CB} to the plane of B,N-core, accompanied by its bond elongation, from the bright **1-S_1-LE** to the dark **1-S_1-CT**. The relative distribution of the PES for **1-S_1-CT** to that for **1-S_1-LE** strongly depends on the surrounding medium, which rationalizes the spectral quenching in a polar solvent medium. While **1-S_1-LE** is more stable than **1-S_1-CT** by ~6 kcal/mol at around 90° in the gas phase, the extent of the relative stability of **1-S_1-LE** with respect to **1-S_1-CT** in that region gradually decreases as the polarity of the surrounding medium increases. Particularly, in the most polar THF medium, the minimum energy in the PES of **1-S_1-LE** is almost comparable to the maximum energy in the PES of **1-S_1-CT**. Therefore, as schematically illustrated in **Figure S23(d)**, the energy barrier from the bright **1-S_1-LE** to the dark **1-S_1-CT** can be interpreted as diminishing with the increasing polarity of the surrounding medium.

OLEDs Fabrication and Characterization

70 nm-thick-indium-tin-oxide (ITO) patterned glass substrates were cleaned using deionized (DI) water and acetone for 10 minutes, each sequentially, in an ultrasonication bath. Then, we put the substrates into a beaker with boiling isopropyl alcohol (IPA) for 10 minutes. Before the deposition, the substrates were treated with UV-ozone for 15 minutes to clean the substrates' surface and adjust the ITO surface's workfunction. The substrates were loaded in an evaporator to form thin films on the substrate under vacuum with a based pressure of 5×10^{-7} torr. For pixelation, we utilized shadow masks for organic and metal layers separately. We controlled the deposition rate of around 1 Å/s for the organic layers. After transferring the substrate within a vacuum, the metal electrodes were finally formed in a metal chamber. The deposition rate was 0.1 Å/s for LiF and 3 Å/s for Al. The devices were encapsulated by a glass-can lid with a UV-resin in an N_2-filled glove box. A Keithely 2400 source meter and a spectrophotometer (PR670) were used for the optoelectrical measurement of the devices.

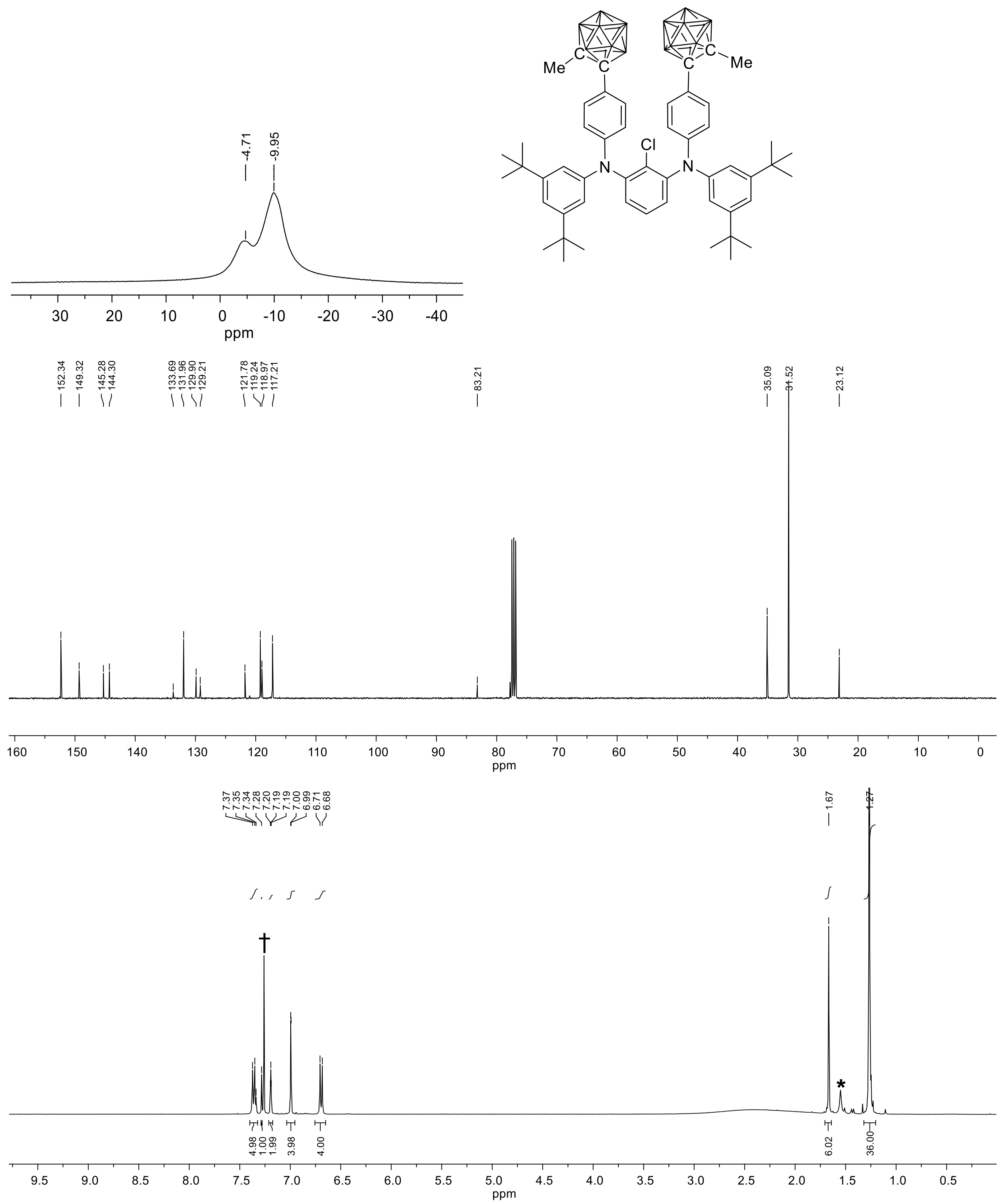

Figure S1. ^{11}B (top), ^{13}C (middle), and ^{1}H (bottom) NMR spectra of **1b** (∗ and † from residual solvents).

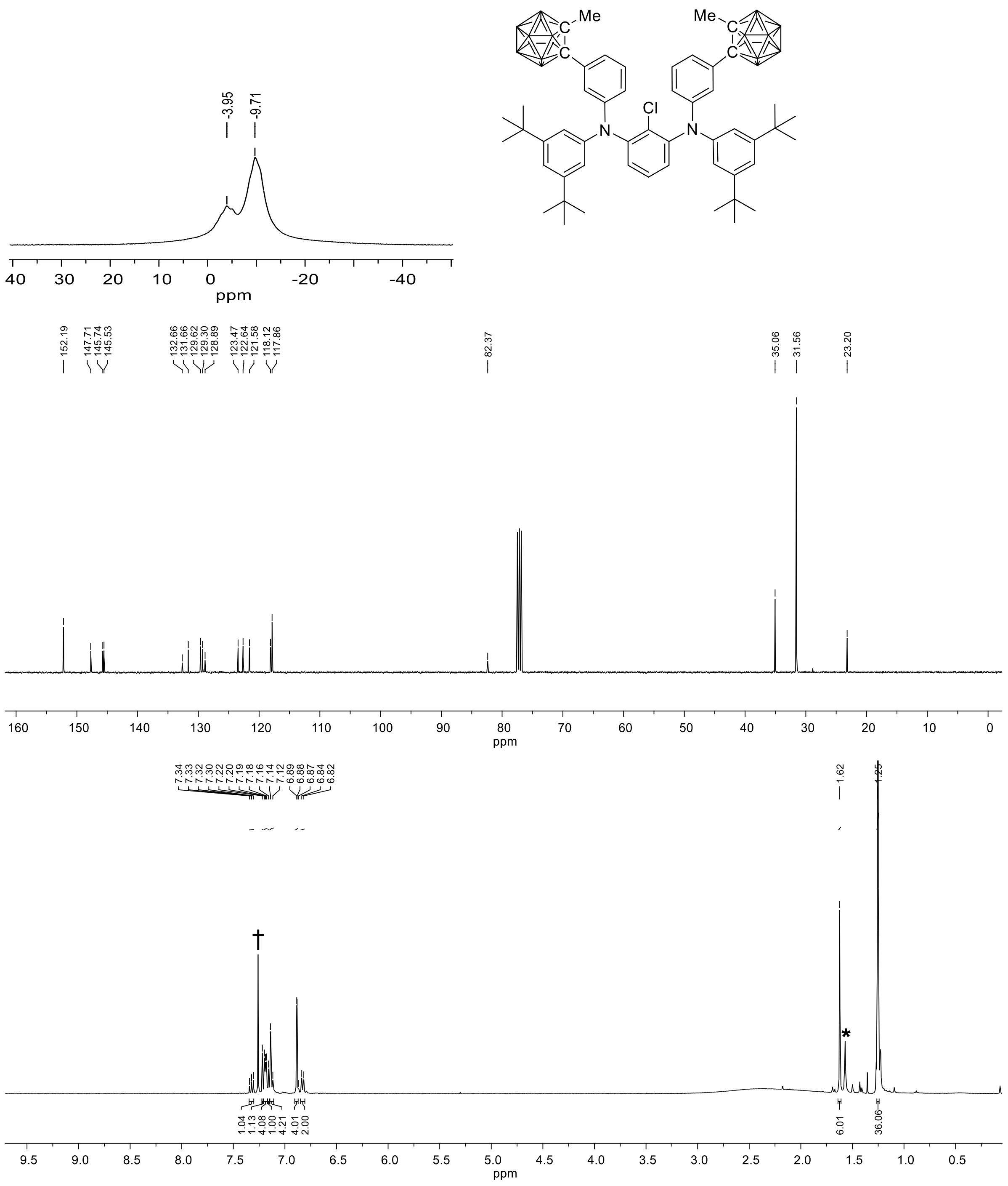

Figure S2. ^{11}B (top), ^{13}C (middle), and ^{1}H (bottom) NMR spectra of **2b** (∗ and † from residual solvents).

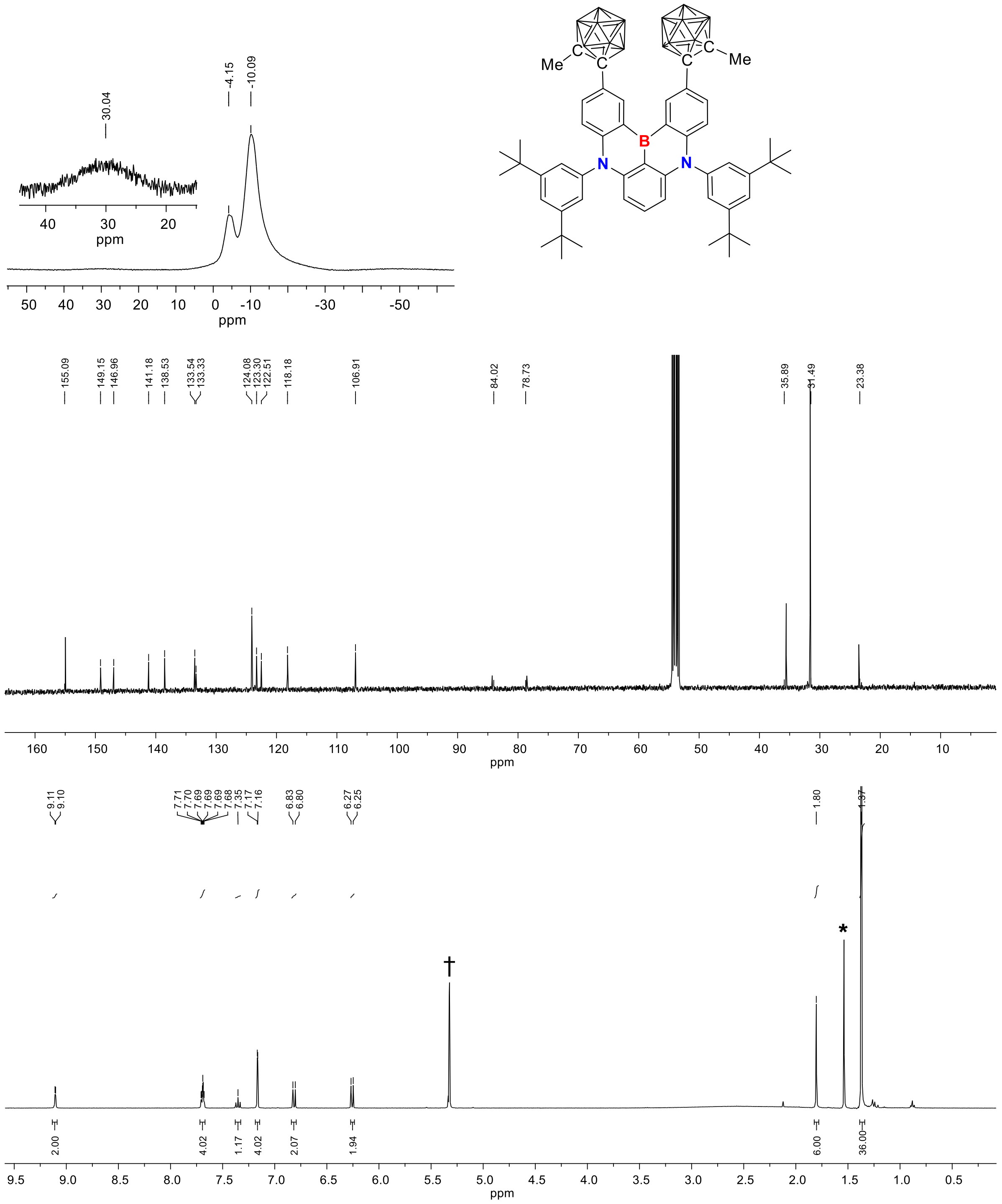

Figure S3. ^{11}B (top), ^{13}C (middle), and ^{1}H (bottom) NMR spectra of **1** (* and † from residual solvents).

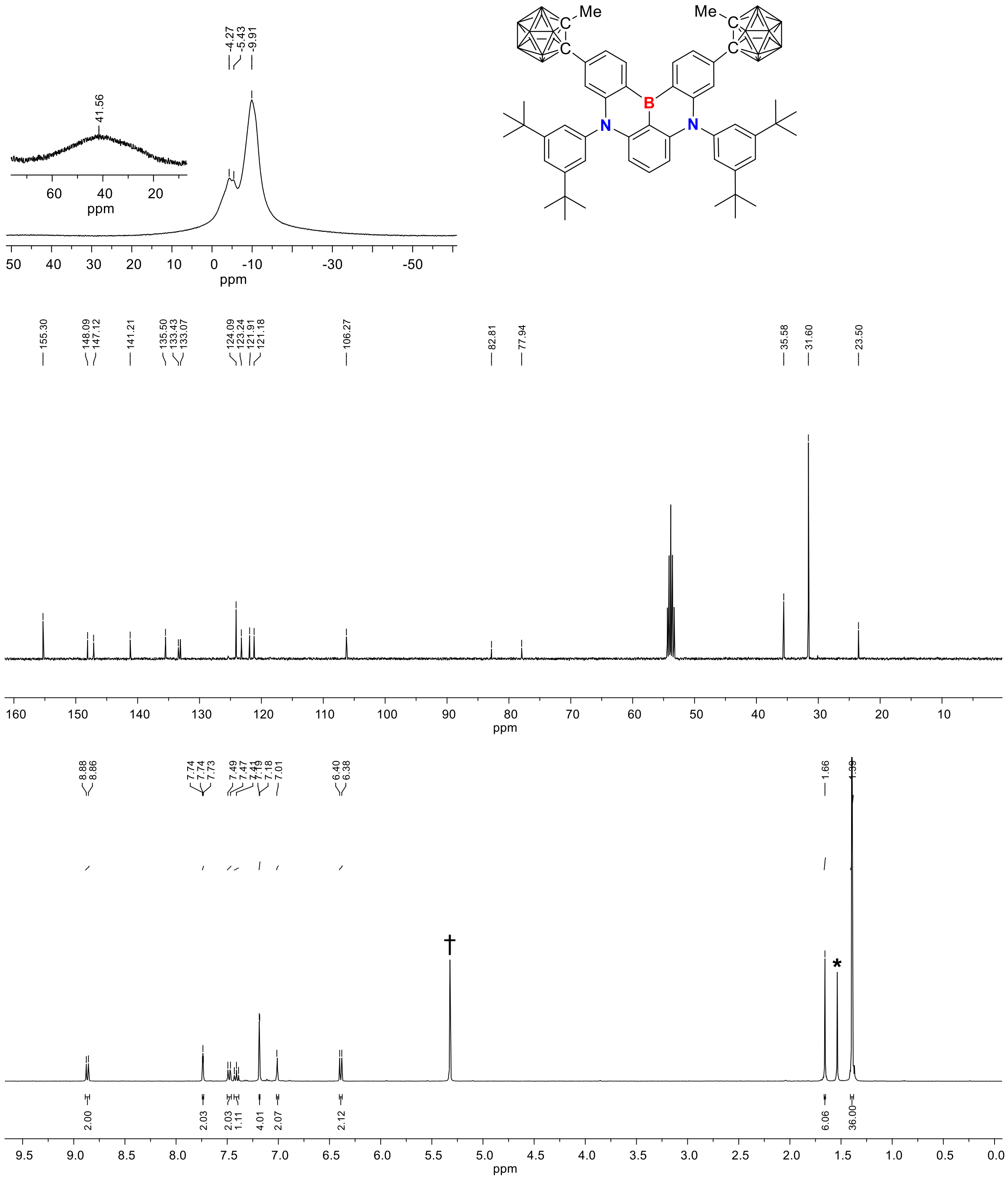

Figure S4. ^{11}B (top), ^{13}C (middle), and ^{1}H (bottom) NMR spectra of **2** (* and † from residual solvents).

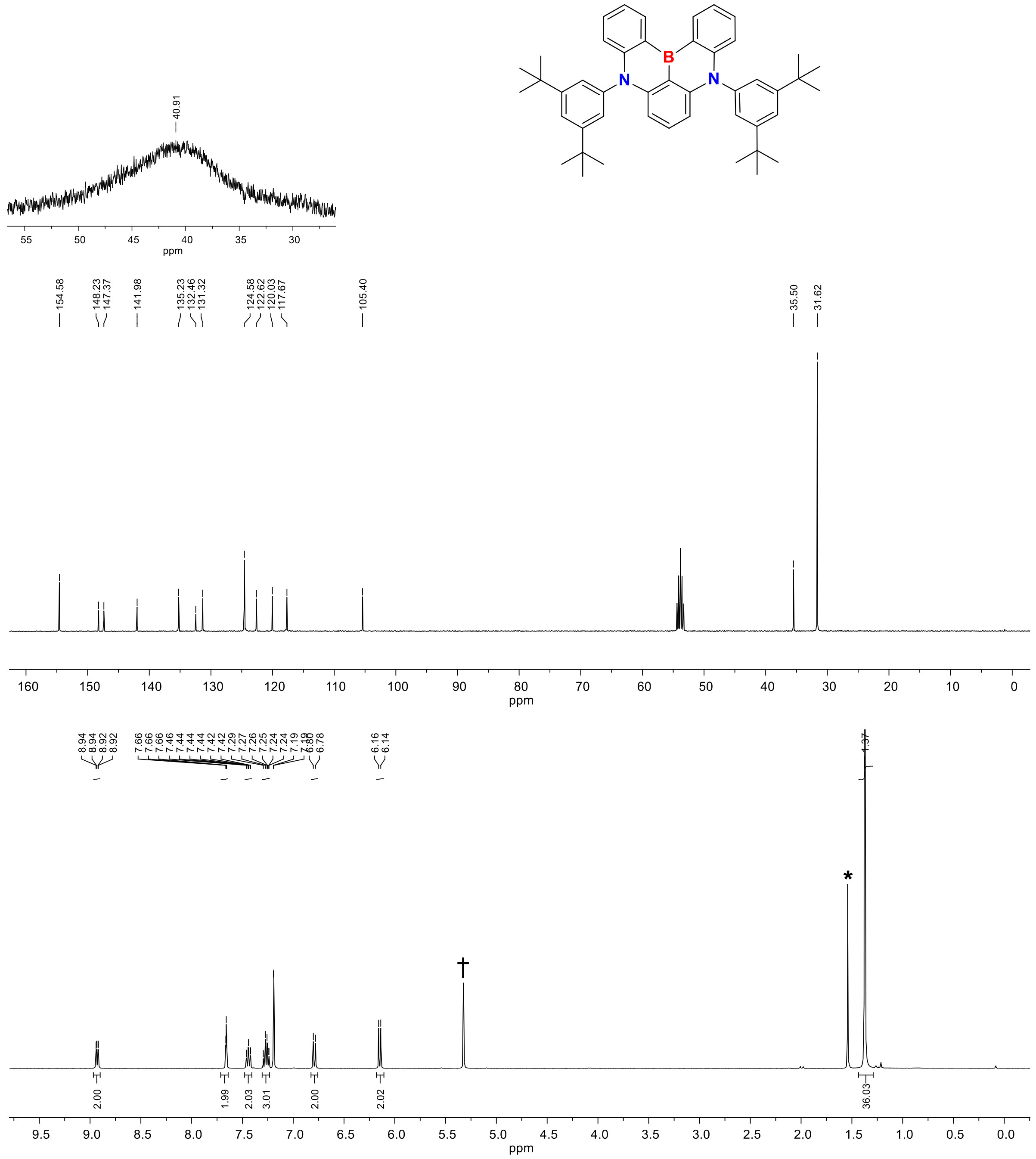

Figure S5. ^{11}B (top), ^{13}C (middle), and ^{1}H (bottom) NMR spectra of **3** (∗ and † from residual solvents).

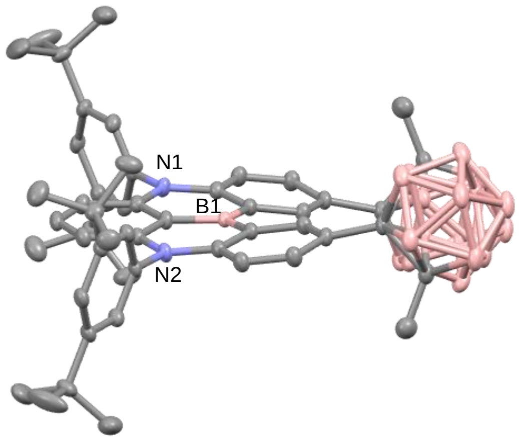

Figure S6. Side views of the crystal structures of 2CB-BuDABNA (**1**) (left) and 3CB-BuDABNA (**2**) (right) (40% thermal ellipsoids). The H atoms and solvent molecules are omitted for clarity.

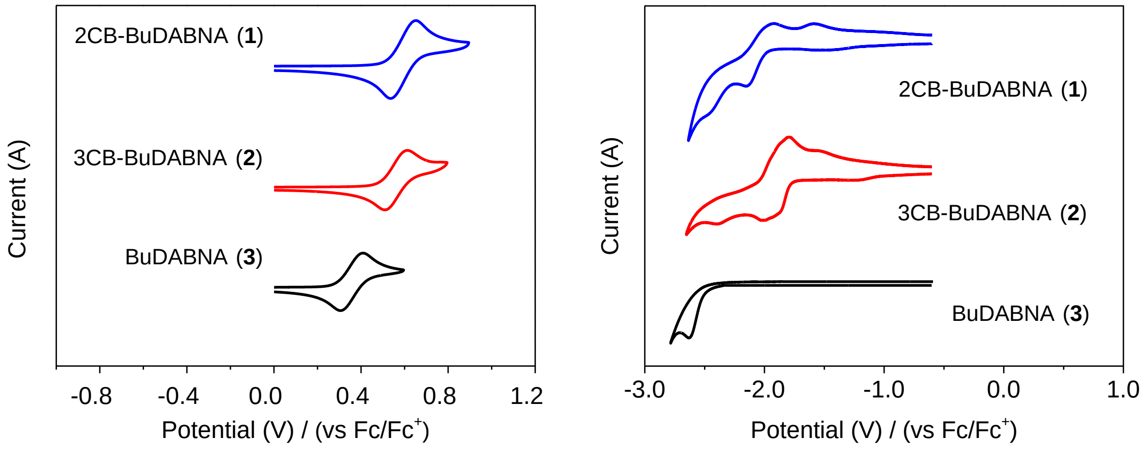

	E_g (eV)[a)]	E_{ox} (V)[b)]	E_{HOMO} (eV)[c)]	E_{LUMO} (eV)[d)]
**1**	2.65	0.59	−5.39	−2.75
**2**	2.50	0.56	−5.36	−2.91
**3**	2.71	0.36	−5.16	−2.44

[a)]Optical bandgap from the absorption onset wavelength; [b)]Half-wave potential ($E_{1/2}$); [c)]From the E_{ox}; [d)]From the E_g and E_{HOMO}.

Figure S7. Cyclic voltammograms of **1–3** showing (left) oxidation in CH_2Cl_2 and (right) reduction in DMF/THF (9:1, v/v). Scan rate = 100 mV/s.

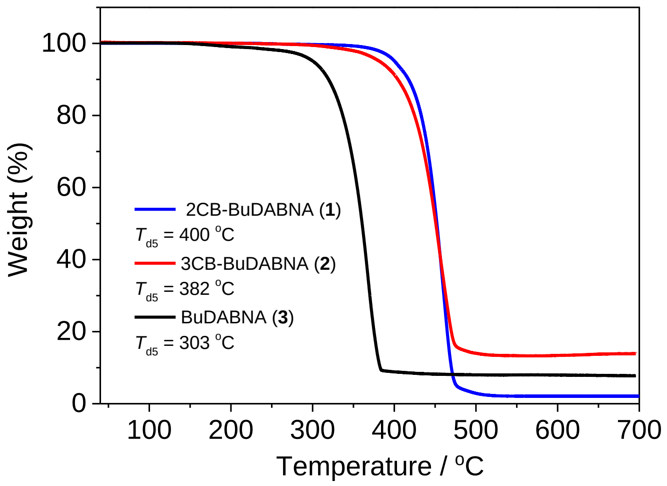

Figure S8. TGA curves of **1–3**.

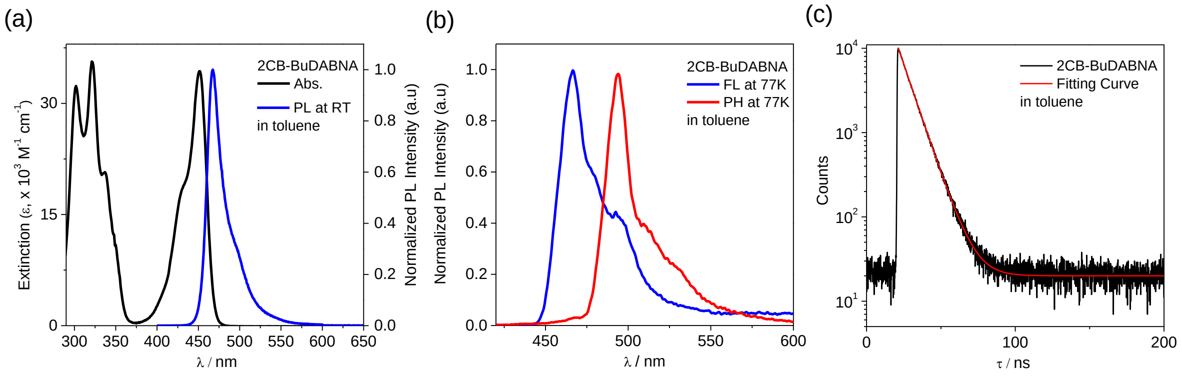

Figure S9. (a) UV/Vis absorption and PL spectra at RT, (b) fluorescence and phosphorescence spectra at 77 K, and (c) transient PL decay of 2CB-BuDABNA (**1**) in toluene (2.0×10^{-5} M).

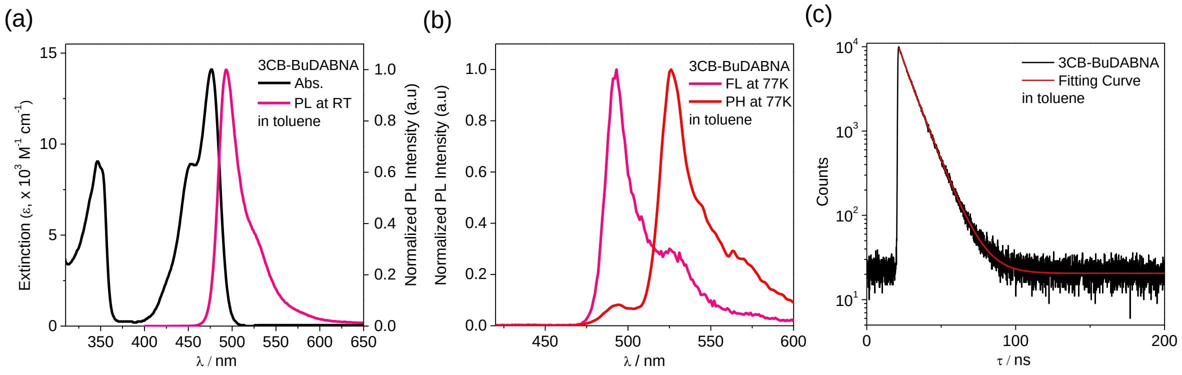

Figure S10. (a) UV/Vis absorption and PL spectra at RT, (b) fluorescence and phosphorescence spectra at 77 K, and (c) transient PL decay of 3CB-BuDABNA (**2**) in toluene (2.0×10^{-5} M).

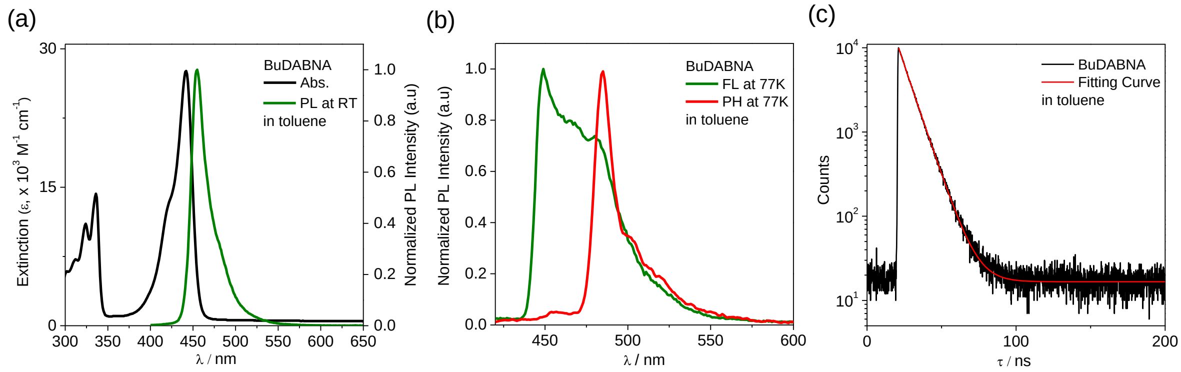

Figure S11. (a) UV/Vis absorption and PL spectra at RT, (b) fluorescence and phosphorescence spectra at 77 K, and (c) transient PL decay of BuDABNA (**3**) in toluene (2.0×10^{-5} M).

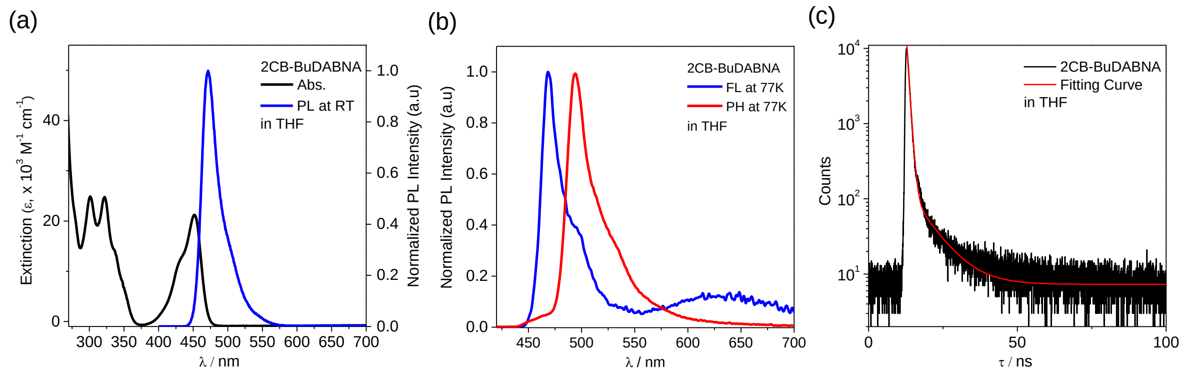

Figure S12. (a) UV/Vis absorption and PL spectra at RT, (b) fluorescence and phosphorescence spectra at 77 K, and (c) transient PL decay of 2CB-BuDABNA (**1**) in THF (2.0×10^{-5} M).

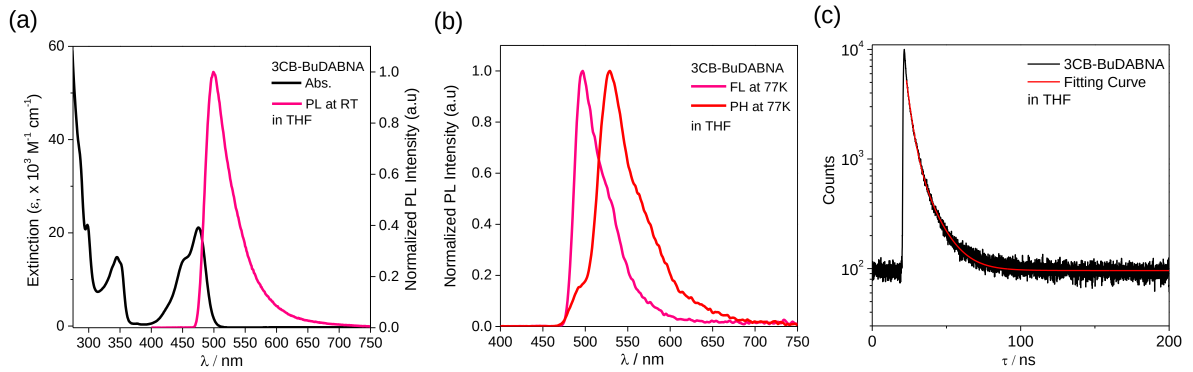

Figure S13. (a) UV/Vis absorption and PL spectra at RT, (b) fluorescence and phosphorescence spectra at 77 K, and (c) transient PL decay of 3CB-BuDABNA (**2**) in THF (2.0 × 10^{-5} M).

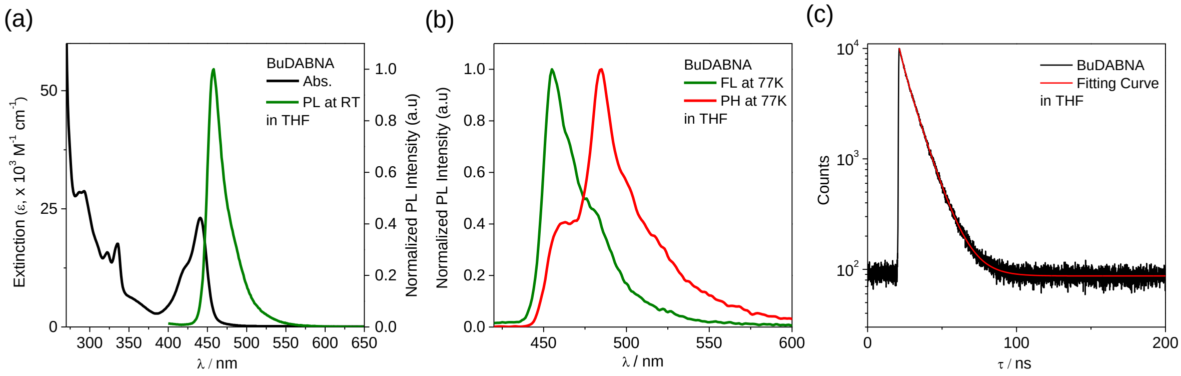

Figure S14. (a) UV/Vis absorption and PL spectra at RT, (b) fluorescence and phosphorescence spectra at 77 K, and (c) transient PL decay of BuDABNA (**3**) in THF (2.0 × 10^{-5} M).

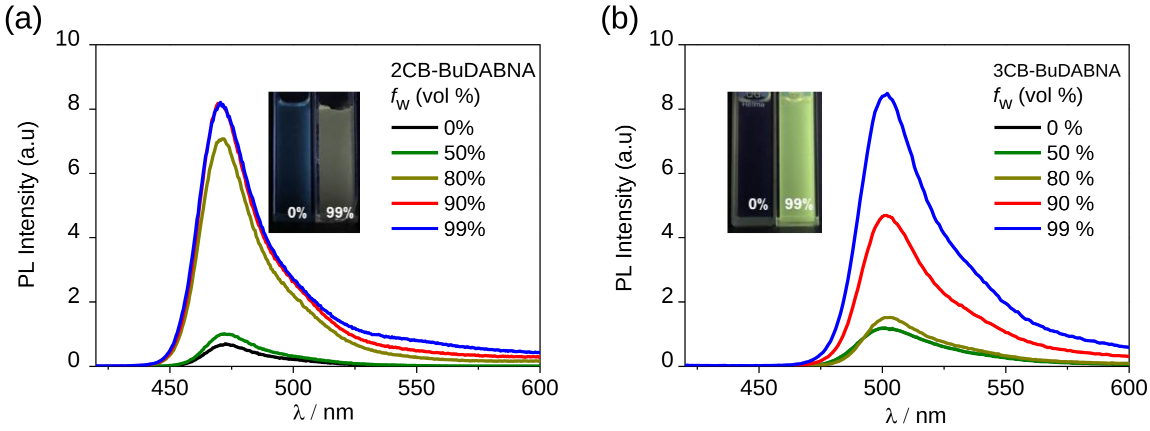

Figure S15. PL spectra of (a) 2CB-BuDABNA (**1**) and (b) 3CB-BuDABNA (**2**) in THF/water mixtures (2.0 × 10^{-5} M) with various water fractions (f_w). Inset: Photos of **1** (left) and **2** (right) in THF/water mixtures (f_w = 0% and 99%) taken under 365 nm excitation.

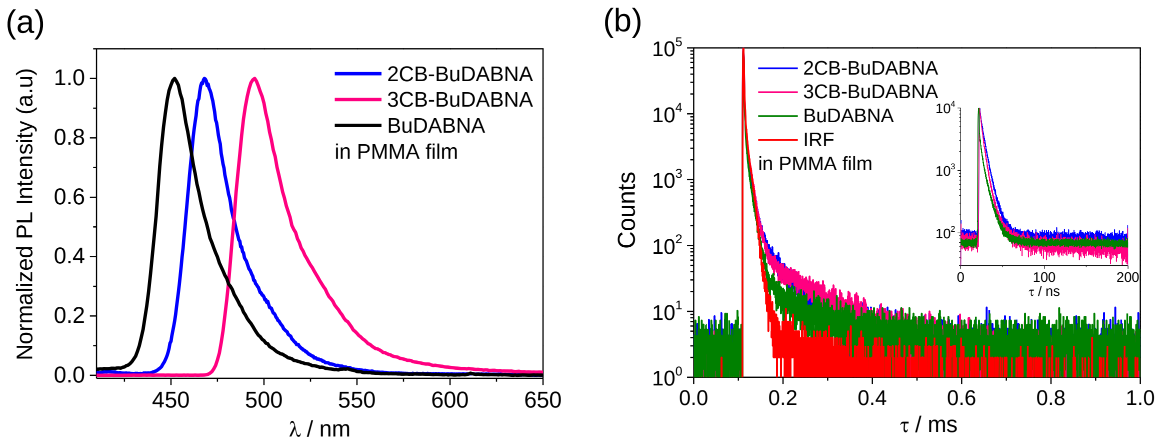

Figure S16. (a) PL spectra and (b) transient PL decay curves of the PMMA films doped with 5 wt% of 2CB-BuDABNA (**1**), 3CB-BuDABNA (**2**), and BuDABNA (**3**). Inset: prompt PL decay.

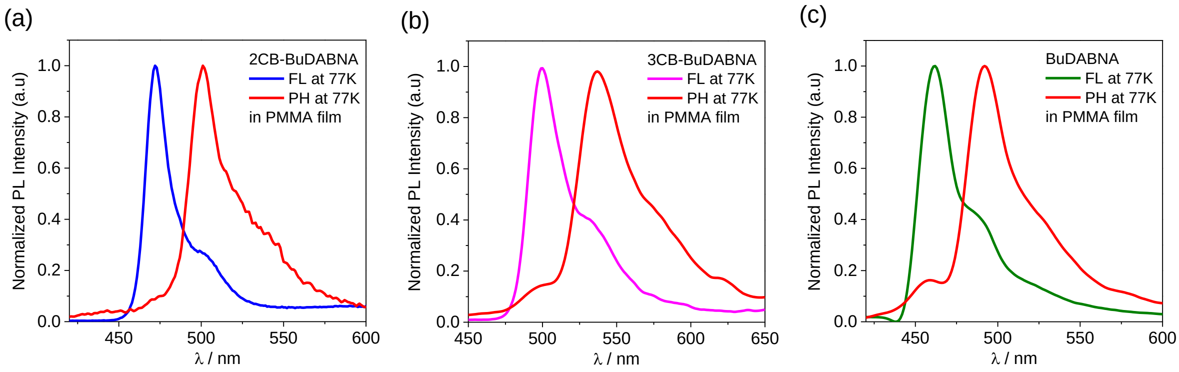

Figure S17. Fluorescence and phosphorescence spectra of the PMMA films doped with 5 wt% of (a) 2CB-BuDABNA (**1**), (b) 3CB-BuDABNA (**2**), and (c) BuDABNA (**3**) at 77 K.

Table S1. The bond length of C_{CB}–C_{CB}, d(C_{CB}–C_{CB}), and its dihedral angle (ψ) with respect to the molecular plane of B,N-core for the S_0, S_1, T_1, and T_2 states of 2CB-BuDABNA (**1**) and 3CB-BuDABNA (**2**), which were optimized in the PBE0/def2-SVP level of theory with SCRF approximation (solvent = toluene).

	2CB-BuDABNA (**1**)		3CB-BuDABNA (**2**)	
	d(C_{CB}–C_{CB}) (Å)	ψ (°)	d(C_{CB}–C_{CB}) (Å)	ψ (°)
S_0	1.69, 1.69	93.0, 93.0	1.68, 1.68	92.9, 92.9
S_1	1.69, 1.69	82.7, 82.7	1.73, 1.73	92.7, 92,7
T_1	1.67, 1.67	83.0, 83.0	1.71, 1.71	92.5, 92.5
T_2	1.67, 1.67	81.1, 81.1	1.72, 1.72	92.3, 92.3

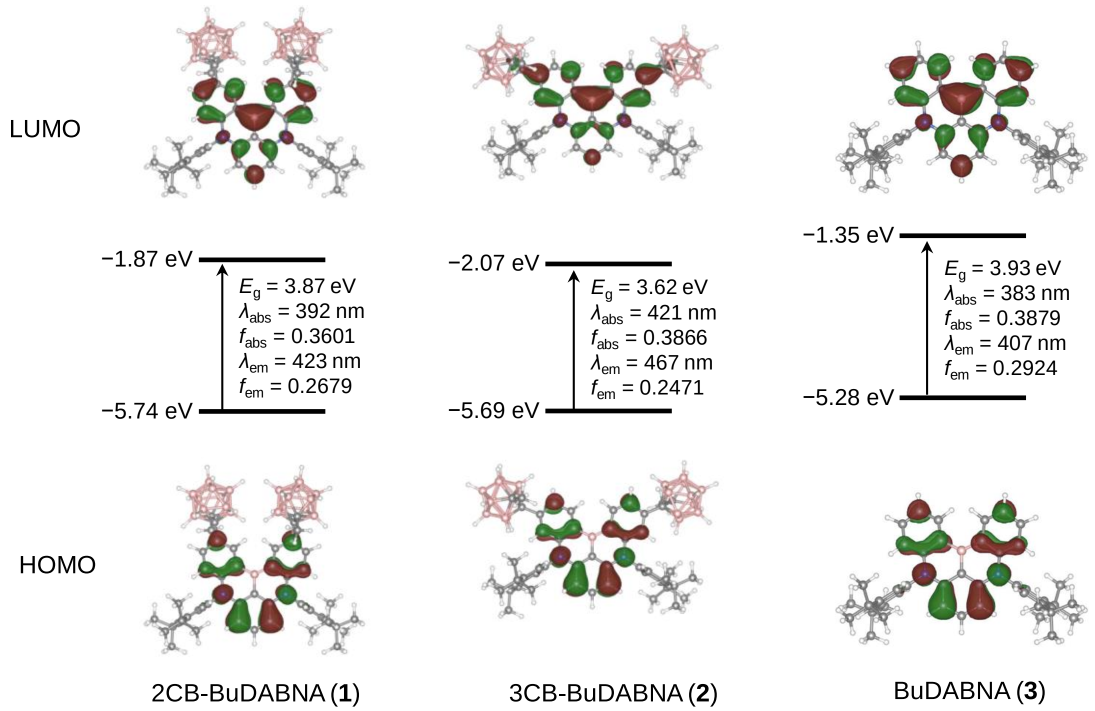

Figure S18. Frontier molecular orbitals of 2CB-BuDABNA (**1**), 3CB-BuDABNA (**2**), and BuDABNA (**3**) (isovalue = 0.03 e/Å^3) at their ground state (S_0) geometries obtained using PBE0/def2-SVP calculations. The numerical values for MO energies, HOMO–LUMO gaps (E_g), maximum absorption (λ_{abs}) and emission (λ_{em}) wavelengths, and oscillator strengths for absorption (f_{abs}) and emission (f_{em}) are provided.

Table S2. Molecular orbital distributions (in %) of 2CB-BuDABNA (**1**), 3CB-BuDABNA (**2**), and BuDABNA (**3**) at their ground state (S_0) optimized geometries.

Compound	MO	Blue region	Red region	CB	other
**1**	HOMO	84.5	9.91	2.54	8.19
	LUMO	21.3	73.2	3.50	2.07
**2**	HOMO	85.8	10.4	0.59	3.17
	LUMO	24.4	63.5	10.9	1.30
**3**	HOMO	86.2	10.7	-	3.06
	LUMO	21.5	77.0	-	1.51

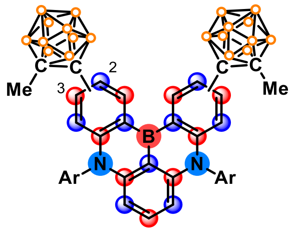

Blue: HOMO part
Red: LUMO part
CB: 2-Me-carborane

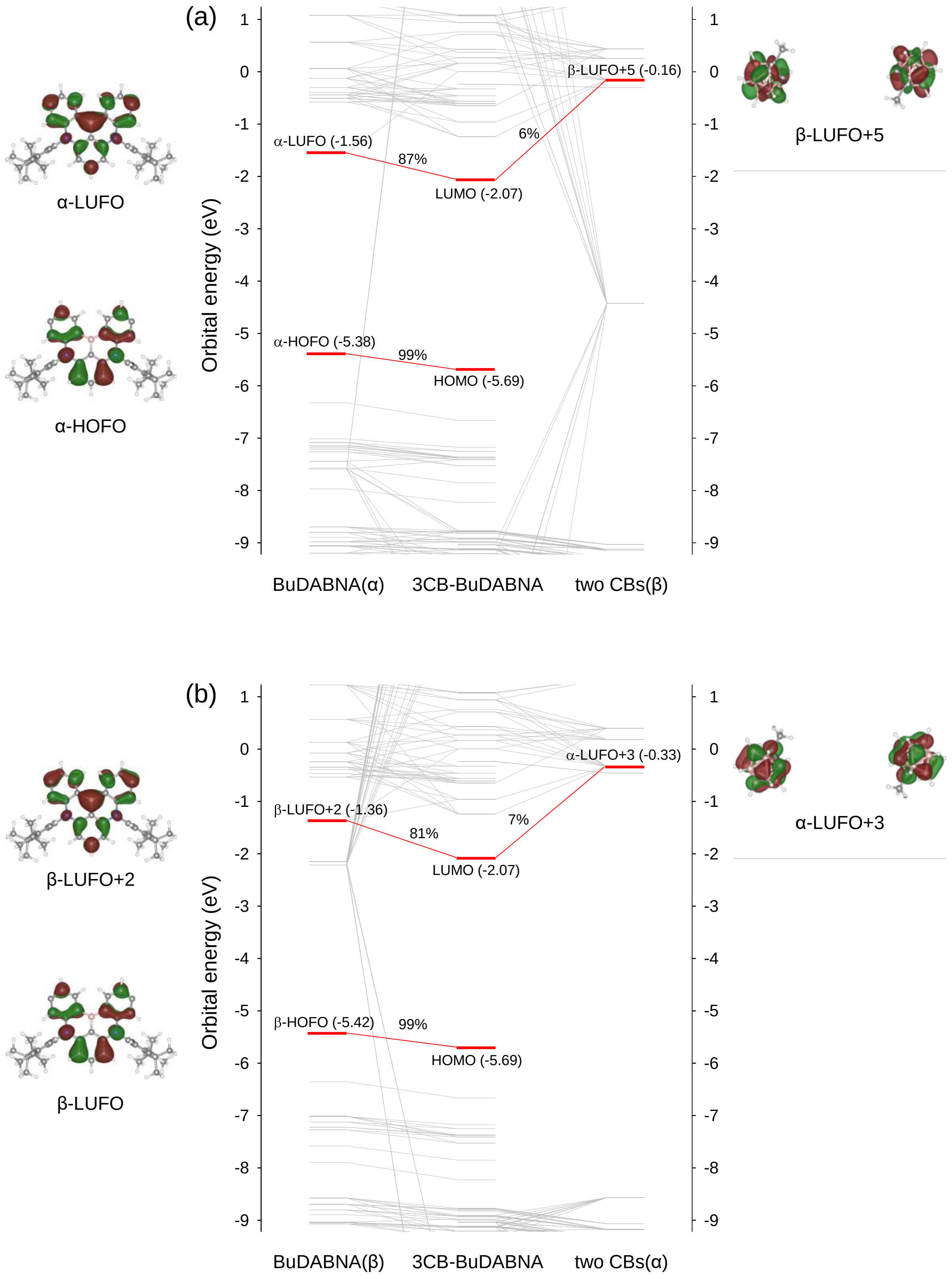

Figure S19. Fragment orbital (FO) diagrams for 3CB-BuDABNA (**2**) between (a) BuDABNA(α) and two CBs(β) and (b) BuDABNA(β) and two CBs(α). The orbital energies (in eV) are presented in parentheses and the contributions (in %) of FOs to HOMO and LUMO are provided.

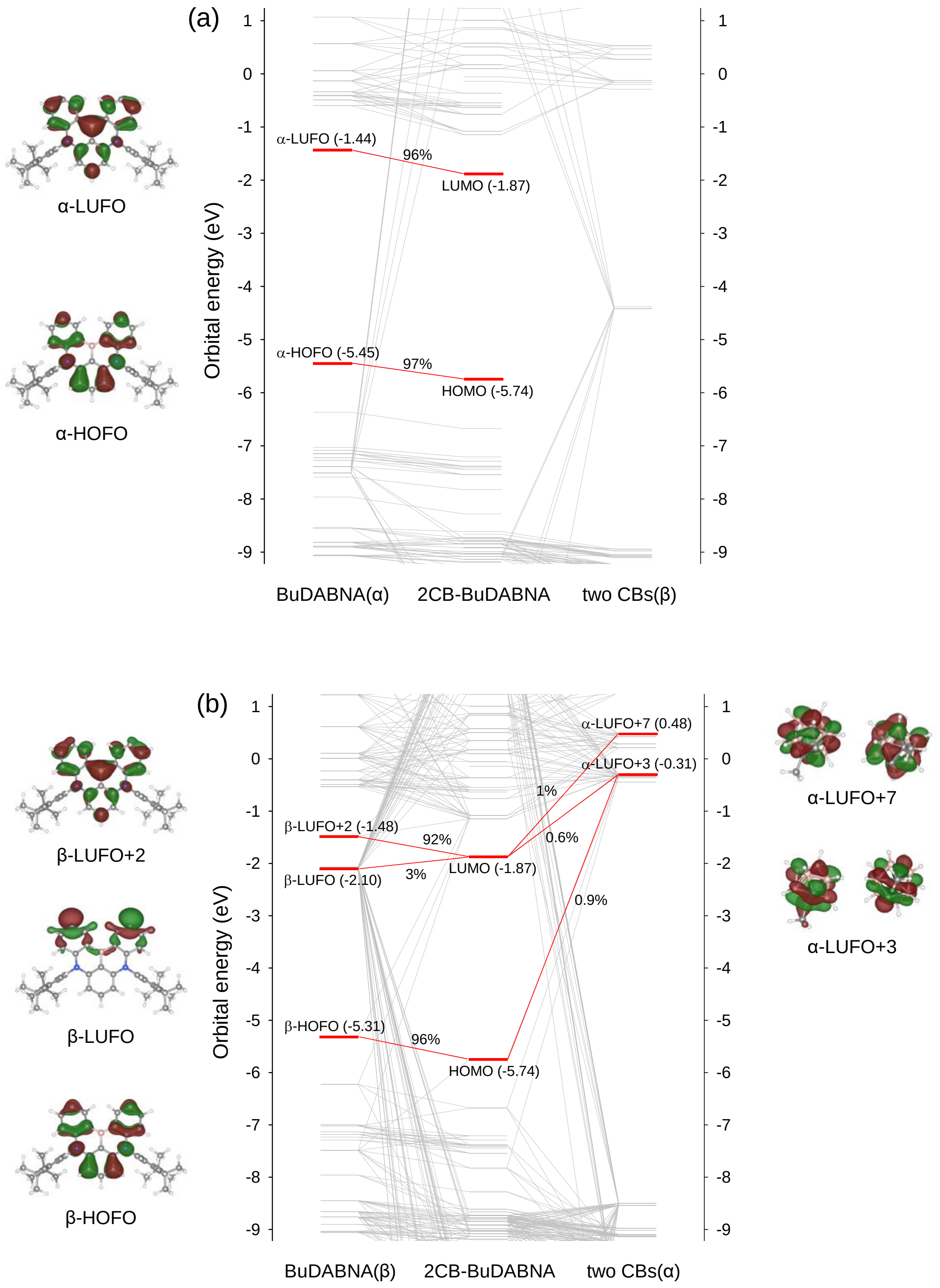

Figure S20. Fragment orbital (FO) diagrams for 2CB-BuDABNA (**1**) between (a) BuDABNA(α) and two CBs(β) and (b) BuDABNA(β) and two CBs(α). The orbital energies (in eV) are presented in parentheses and the contributions (in %) of FOs to HOMO and LUMO are provided.

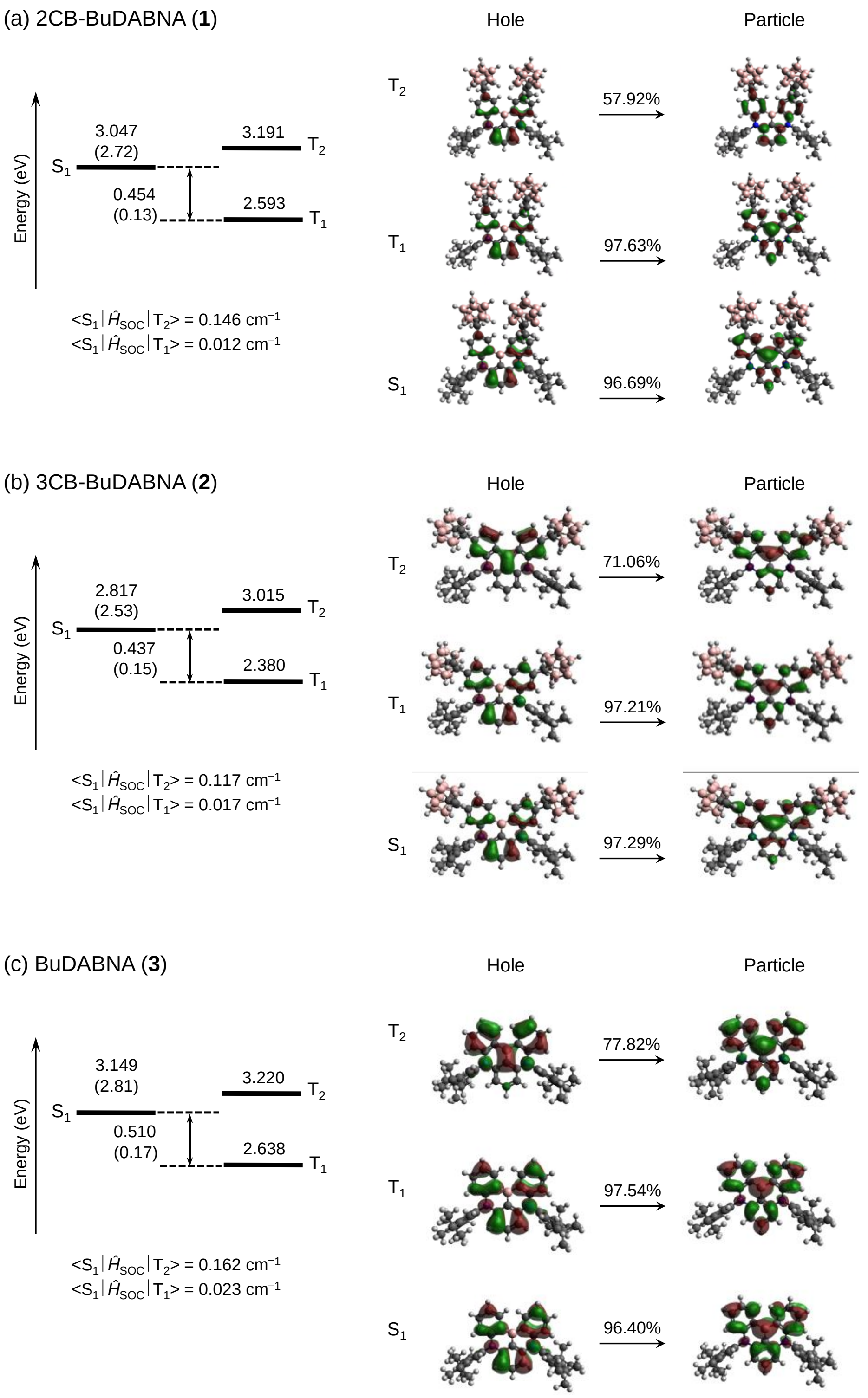

Figure S21. Natural transition orbitals (NTOs, isovalue = 0.03 e/Å^3) of 2CB-BuDABNA (**1**), 3CB-BuDABNA (**2**), and BuDABNA (**3**) for the transitions from S_0 to S_1, T_1, and T_2 states. The experimental ΔE_{ST} values and the corrected S_1 energies which were corrected along with the experimentally measured ΔE_{ST} are presented in parentheses. Computationally obtained

spin-orbit coupling matrix elements for the RISC processes between the S_1 and T_n (n = 1 and 2) states at 298 K are provided.

Table S3. NTO contributions (in %) in the S_1 and T_1 excited states of 2CB-BuDABNA (**1**) and 3CB-BuDABNA (**2**).

State		Blue region	Red region	CB	other
**S_1**					
**1**	Hole	79.0	10.4	2.43	2.92
	Particle	21.5	72.6	3.49	0.72
**2**	Hole	80.8	10.8	0.62	2.80
	Particle	24.5	63.9	9.92	1.13
**T_1**					
**1**	Hole	79.3	10.0	2.59	2.65
	Particle	20.6	75.2	2.30	0.72
**2**	Hole	80.1	10.6	0.68	2.68
	Particle	22.7	68.5	7.42	0.77

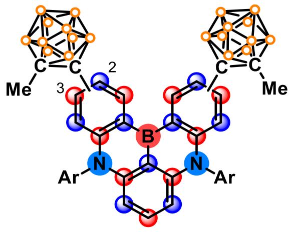

Blue: Hole part
Red: Particle part
CB: 2-Me-carborane

Table S4. The bond lengths of C_{CB}–C_{CB}, $d(C_{CB}–C_{CB})$, and its dihedral angles (ψ) with respect to the molecular plane of B,N-core for the local minimum S_1 states of 2CB-BuDABNA (**1**) depending on the surrounding medium (toluene, gas phase, and THF). The corresponding geometric parameters for S_0 states of **1** are presented for the comparison. The oscillator strengths (f) of local minimum S_1 states and the relative energies (in kcal/mol) of **1-S₁-CT** with respect to **1-S₁-LE** are also provided. Note that **1-S1-LE** (in toluene medium) is identical to the S_1 of **1** in **Table S2**.

		**1-S_0**	**1-S_1-LE**	**1-S_1-CT**
toluene	$d(C_{CB}\text{-}C_{CB})$ (Å)	1.69, 1.69	1.69, 1.69	2.45, 1.67
	ψ (°)	93.0, 93.0	82.7, 82.7	4.4, 67.0
	Oscillator strength (f)		0.2679	0.0018
	Relative energy (kcal/mol)		0.00	−6.01
gas phase	$d(C_{CB}\text{-}C_{CB})$ (Å)	1.69, 1.69	1.68,1.68	2.46, 1.67
	ψ (°)	80.2, 80.2	80.8, 80.8	4.43, 66.1
	Oscillator strength (f)		0.1964	0.0012
	Relative energy (kcal/mol)		0.00	−5.31
THF	$d(C_{CB}\text{-}C_{CB})$ (Å)	1.69, 1.69	1.69, 1.69	2.44, 1.67
	ψ (°)	93.5, 93.5	82.9, 82.9	5.0, 67.8
	Oscillator strength (f)		0.3329	0.0032
	Relative energy (kcal/mol)		0.00	−6.44

Table S5. NTO contributions (in %) in two local minimum S_1 states of 2CB-BuDABNA (**1**) in toluene medium.

		MR-core	CB	other
**1-S$_1$-LE**	Hole	89.4	2.43	2.92
	Particle	94.1	3.49	0.72
**1-S$_1$-CT**	Hole	93.1	3.61	3.33
	Particle	2.66	97.3	0.03

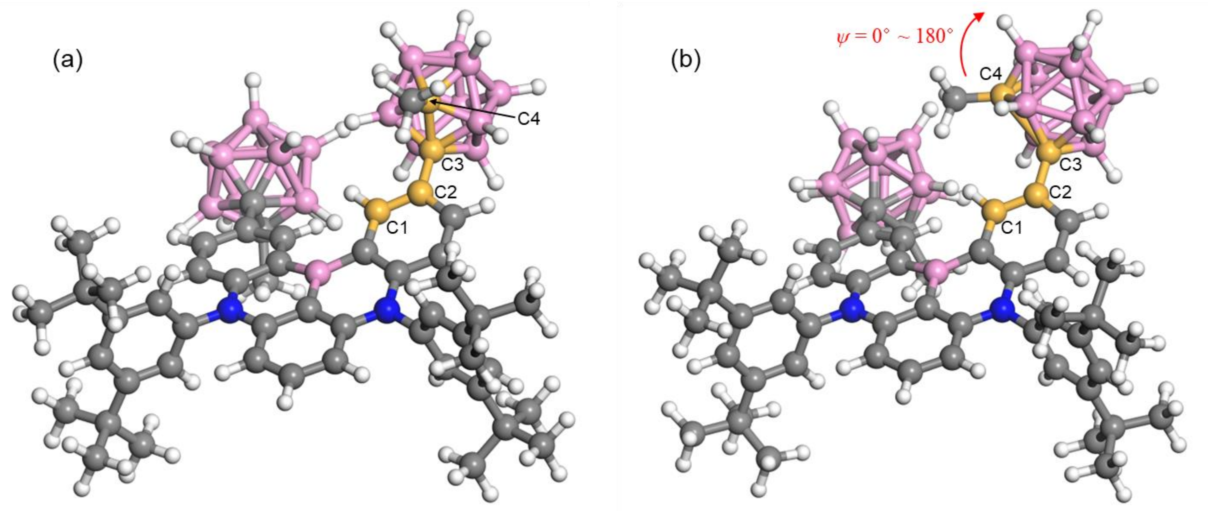

Figure S22. Optimzed geometries of (a) **1-S$_1$-LE** and (b) **1-S$_1$-CT** in toluene medium. The dihedral angle (ψ) is defined with four atoms from C1 to C4 (orange-colored) and the bond length between C3 and C4 corresponds to $d(C_{CB}–C_{CB})$. The direction of dihedral rotation is indicated with the red arrow in (b), and the rotational PES is constructed by increasing the dihedral angle (ψ) from 0° to 180° with 10° increments.

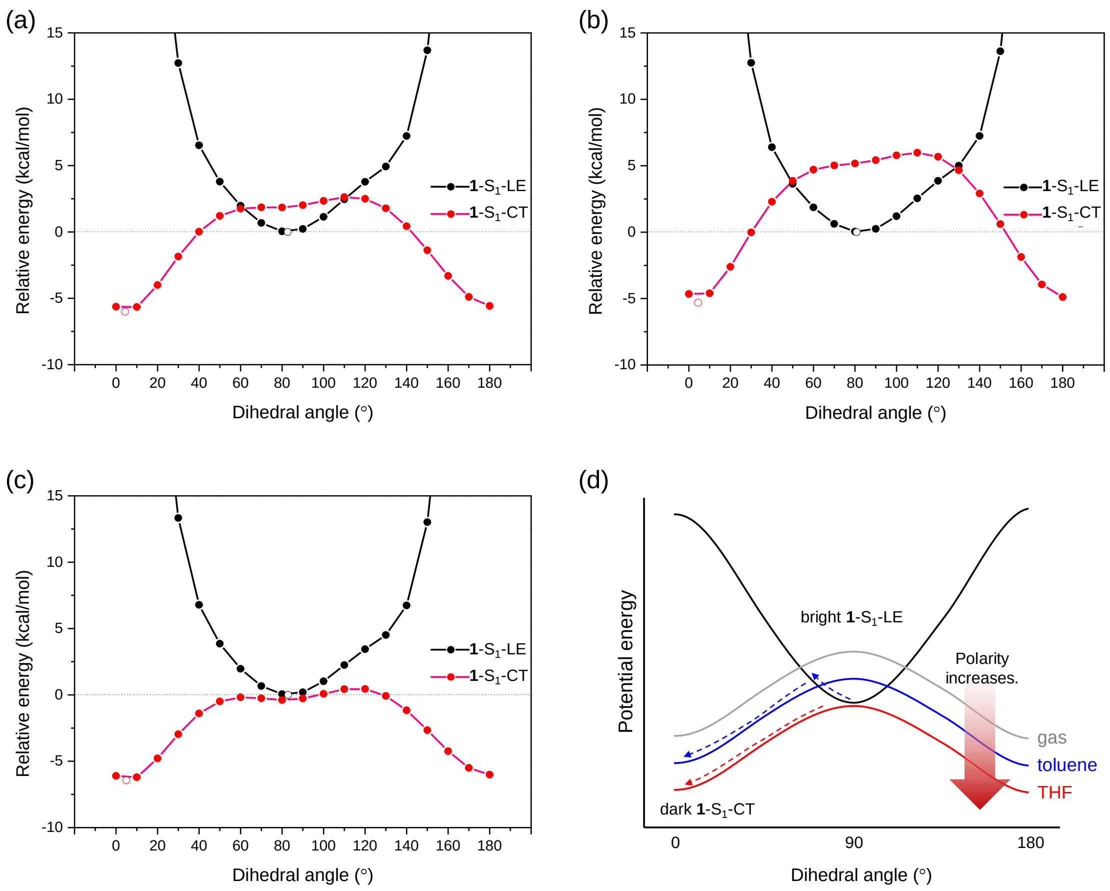

Figure S23. Potential energy surfaces (PESs) for **1-S₁-LE** and **1-S₁-CT** states in (a) toluene, (b) gas phase, and (c) THF medium, which are constructed by increasing the dihedral angle (ψ) from 0° to 180° with 10° increments (see **Figure S21**). The numerical values of relative energies were evaluated with respect to that of optimized **1-S₁-LE**. The relative energies corresponding to optimized **1-S₁-LE** and **1-S₁-CT** are indicated with open circles. (d) Schematic diagram for the PES of bright (emissive) S_1 state, i.e., **1-S₁-LE**, and dark (non-emissive) S_1 state, i.e., **1-S1-CT**, depending on the surrounding medium.

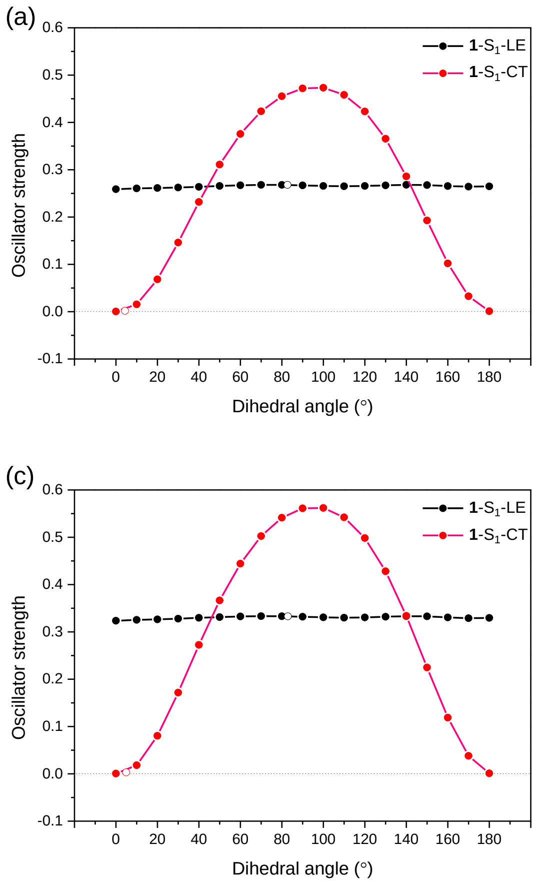

Figure S24. The variation of oscillator strengths (*f*) of **1-S$_1$-LE** and **1-S$_1$-CT** at the dihedral angle (ψ) from 0° to 180° with 10° increments in (a) toluene, (b) gas phase, and (c) THF medium (see **Figure S21** and **S22**). The oscillator strengths corresponding to optimized **1-S$_1$-LE** and **1-S$_1$-CT** are indicated with open circles.

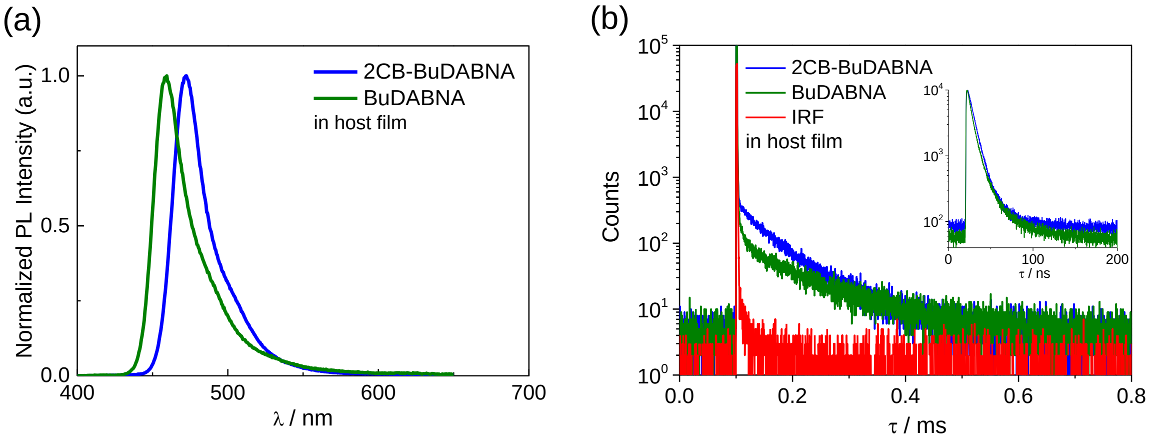

Figure S25. (a) PL spectra and (b) transient PL decay curves of the SiCzCz:SiTrzCz2 host films doped with 5 wt% of 2CB-BuDABNA (**1**) and BuDABNA (**3**). Inset: prompt PL decay.

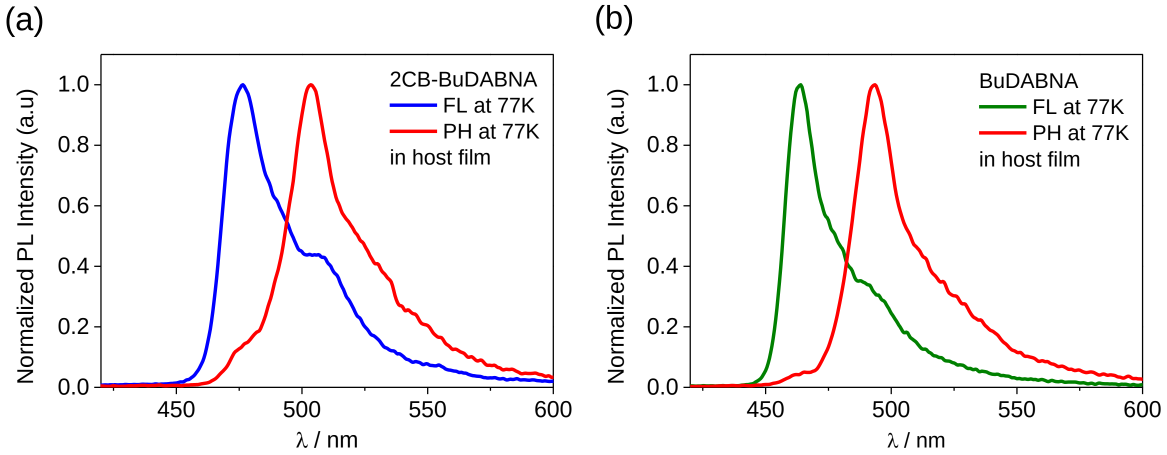

Figure S26. Fluorescence and phosphorescence spectra of the SiCzCz:SiTrzCz2 host films doped with 5 wt% of (a) 2CB-BuDABNA (**1**) and (b) BuDABNA (**3**) at 77 K.

Table S6. Photophysical data and rate constants of 5 wt% 2CB-BuDABNA (**1**) and BuDABNA (**3**) in SiCzCz:SiTrzCz2 (70:30 wt%) host films.

compd	λ_{PL} (nm)	FWHM [nm][a)]	Φ_{PL} [%][b)]	Φ_p/Φ_d [%][c)]	τ_p (ns)/ τ_d [μs][d)]	ΔE_{ST} [eV][e)]	k_r [10^7 s^{-1}][f)]	k_{ISC} [10^7 s^{-1}][f)]	k_{RISC} [10^4 s^{-1}][f)]
**1**	472	26	93	68/25	9.30/69.1	0.140	7.28	3.48	1.66
**3**	459	27	92	84/8	9.19/92.6	0.157	9.09	1.79	0.66

[a)]Full width at half maximum of the PL spectrum; [b)]Absolute PLQYs; [c)]PLQYs of prompt (Φ_p) and delayed (Φ_d) fluorescence; [d)]Lifetimes of prompt (τ_p) and delayed (τ_d) PL decay components; [e)]$\Delta E_{ST} = E_S - E_T$; Singlet ($E_S$) and triplet ($E_T$) energies estimated from the peak wavelengths of the fluorescence and phosphorescence spectra at 77 K; [f)]Rate constants of fluorescence radiative decay, $k_r = \Phi_p/\tau_p$, intersystem crossing, $k_{ISC} = (1 - \Phi_p)/\tau_p$, and reverse intersystem crossing, $k_{RISC} = (k_p k_d \Phi_d)/(k_{ISC}\Phi_p) = \Phi_d/(k_{ISC}\tau_p\tau_d\Phi_p)$, where Φ_p and Φ_d are the quantum yields for prompt and delayed fluorescence ($\Phi_{PL} = \Phi_p + \Phi_d$), respectively.[19]

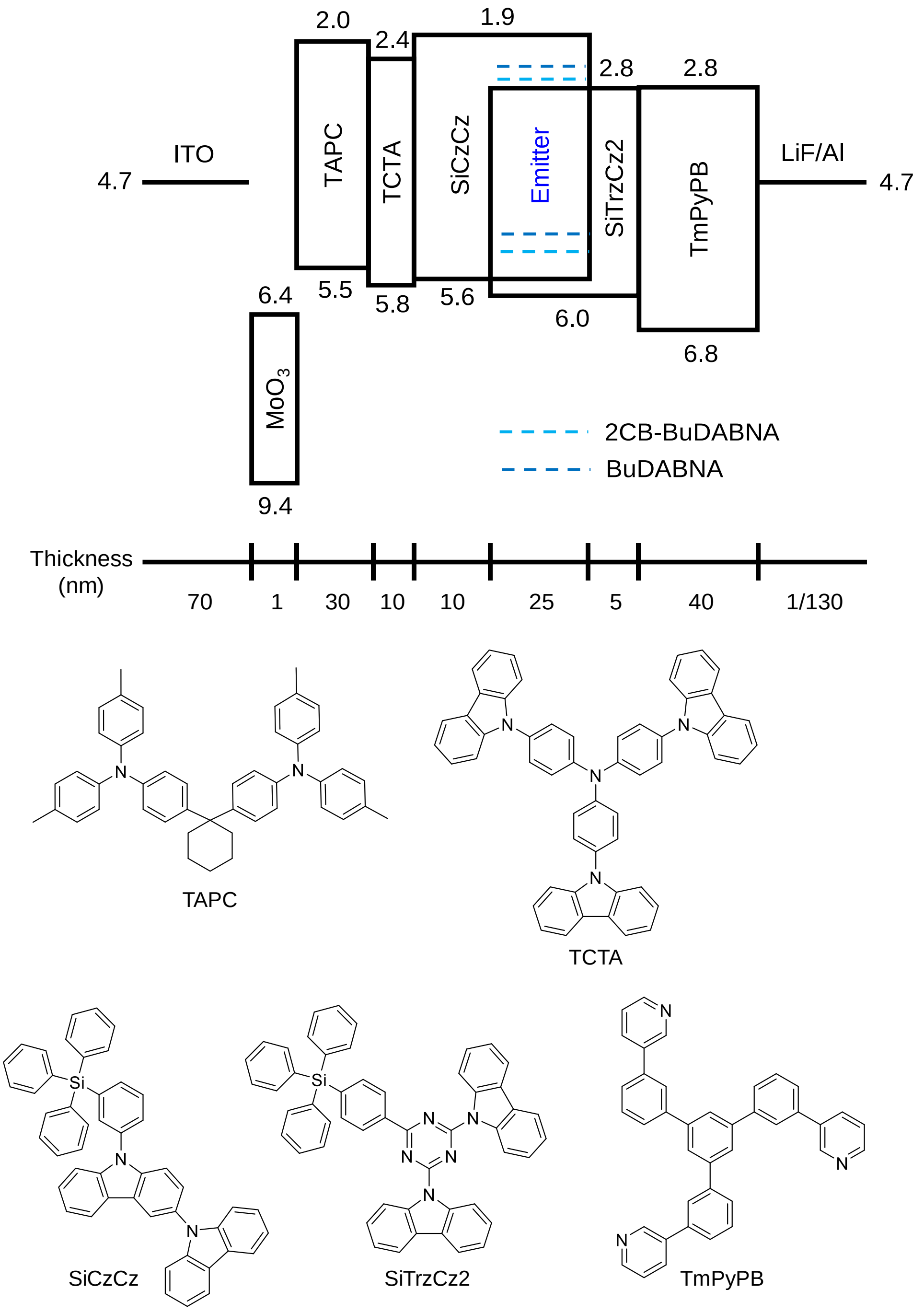

Figure S27. (Top) Device structure and energy level diagram (in eV) of the TADF-OLEDs relative to the vacuum level and (bottom) chemical structures of the consisting layers.

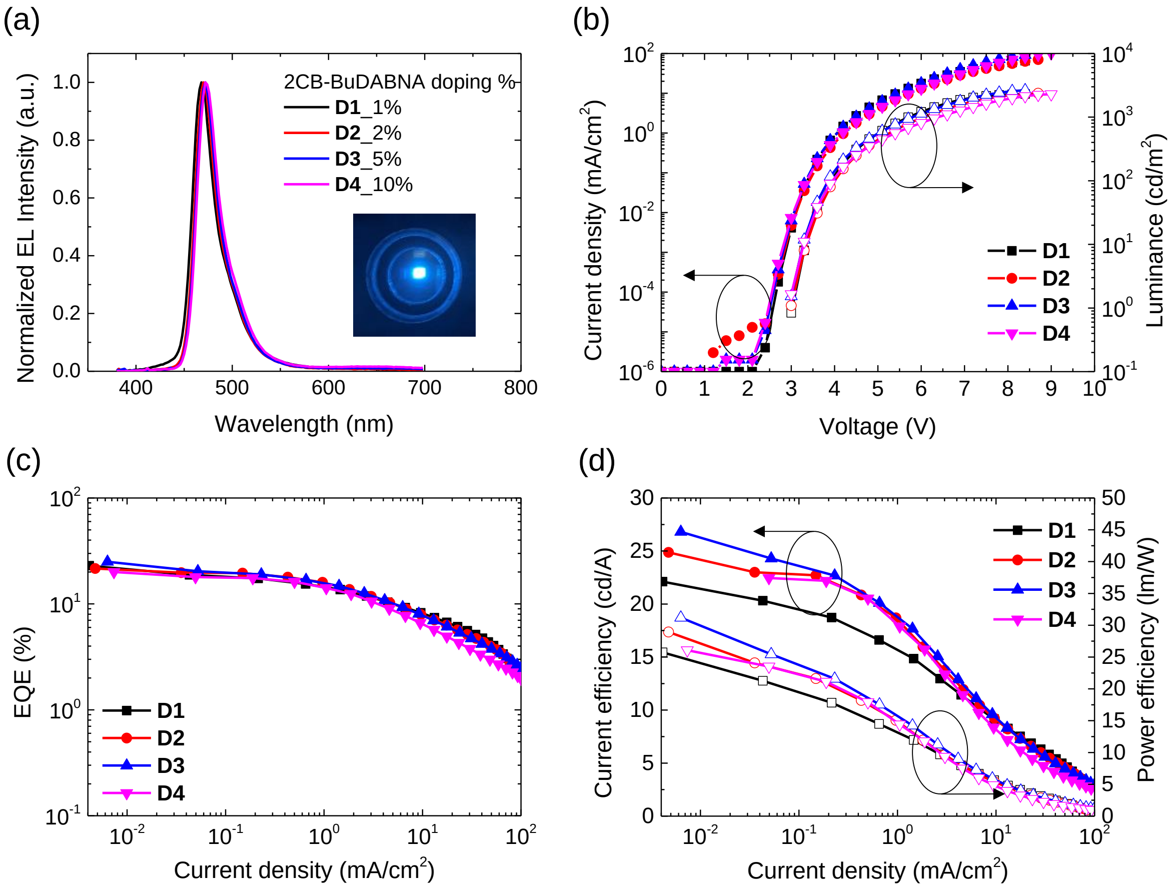

Figure S28. (a) EL spectra of TADF-OLEDs (**D1**–**D4**) based on 2CB-BuDABNA (**1**) emitter and photograph of the working **D3** device. (b) Current density–voltage–luminance (*J*–*V*–*L*) characteristics of **D1**–**D4**. (c) External quantum efficiency–current density (EQE–*J*) characteristics of **D1**–**D4**. (d) Current efficiency–current density (CE–*J*) and power efficiency–current density (PE–*J*) characteristics of **D1**–**D4**.

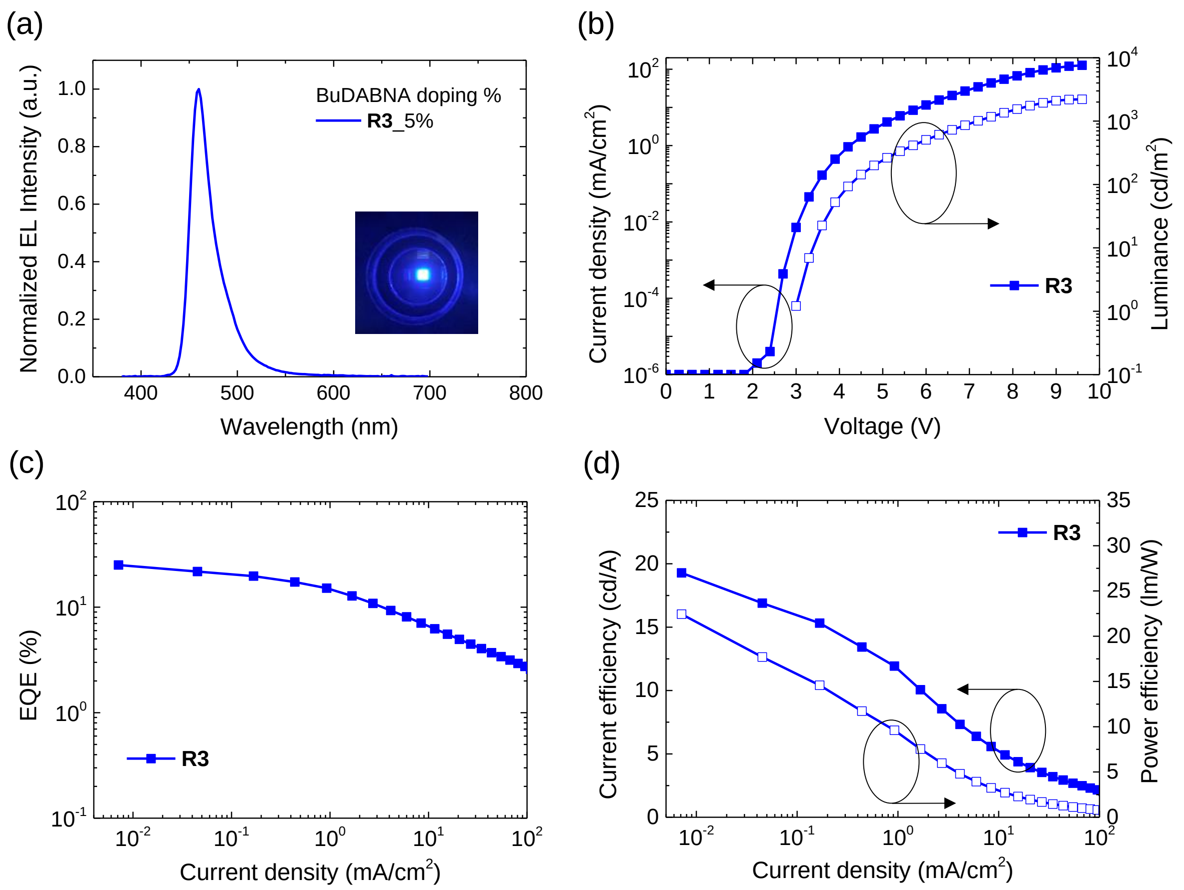

Figure S29. (a) EL spectrum of TADF-OLED (**R3**) based on BuDABNA (**3**) emitter and photograph of the working **R3** device. (b) Current density–voltage–luminance (*J*–*V*–*L*) characteristics of **R3**. (c) External quantum efficiency–current density (EQE–*J*) characteristics of **R3**. (d) Current efficiency–current density (CE–*J*) and power efficiency–current density (PE–*J*) characteristics of **R3**.

References

1. Kim, T.; Kim, H.; Lee, K. M.; Lee, Y. S.; Lee, M. H. *Inorg. Chem.* **2013**, *52*, 160.

2. Nakatsuka, S.; Gotoh, H.; Kinoshita, K.; Yasuda, N.; Hatakeyama, T. *Angew. Chem. Int. Ed.* **2017**, *56*, 5087.

3. Spek, A. L. PLATON, A Multipurpose Crystallographic Tool, University of Ultrecht, Ultrecht, The Netherlands, 2003.

4. (a) Kim, H. J.; Yasuda, T. *Adv. Opt. Mater.* **2022**, *10*, 2201714. (b) Kondo, Y.; Yoshiura, K.; Kitera, S.; Nishi, H.; Oda, S.; Gotoh, H.; Sasada, Y.; Yanai, M.; Hatakeyama, T. *Nat. Photonics* **2019**, *13*, 678. (c) Hatakeyama, T.; Shiren, K.; Nakajima, K.; Nomura, S.; Nakatsuka, S.; Kinoshita, K.; Ni, J.; Ono, Y.; Ikuta, T. *Adv. Mater.* **2016**, *28*, 2777.

5. Adamo, C.; Barone, V. *J. Chem. Phys.* **1999**, *110*, 6158.

6. Weigend, F.; Ahlrichs, R. *Phys. Chem. Chem. Phys.* **2005**, *7*, 3297.

7. Frisch, M. J.; Trucks, G. W.; Schlegel, H. B.; Scuseria, G. E.; Robb, M. A.; Cheeseman, J. R.; Scalmani, G.; Barone, V.; Petersson, G. A.; Nakatsuji, H.; Li, X.; Caricato, M.; Marenich, A. V.; Bloino, J.; Janesko, B. G.; Gomperts, R.; Mennucci, B.; Hratchian, H. P.; Ortiz, J. V.; Izmaylov, A. F.; Sonnenberg, J. L.; Williams-Young, D.; Ding, F.; Lipparini, F.; Egidi, F.; Goings, J.; Peng, B.; Petrone, A.; Henderson, T.; Ranasinghe, D.; Zakrzewski, V. G.; Gao, J.; Rega, N.; Zheng, G.; Liang, W.; Hada, M.; Ehara, M.; Toyota, K.; Fukuda, R.; Hasegawa, J.; Ishida, M.; Nakajima, T.; Honda, Y.; Kitao, O.; Nakai, H.; Vreven, T.; Throssell, K.; Montgomery, J. A., Jr.; Peralta, J. E.; Ogliaro, F.; Bearpark, M. J.; Heyd, J. J.; Brothers, E. N.; Kudin, K. N.; Staroverov, V. N.; Keith, T. A.; Kobayashi, R.; Normand, J.; Raghavachari, K.; Rendell, A. P.; Burant, J. C.; Iyengar, S. S.; Tomasi, J.; Cossi, M.; Millam, J. M.; Klene, M.; Adamo, C.; Cammi, R.; Ochterski, J. W.; Martin, R. L.; Morokuma, K.; Farkas, O.; Foresman, J. B.; Fox, D. J. Gaussian 16, Revision C.01, Gaussian, Inc., Wallingford CT, **2019**.

8. Hirata, S.; Head-Gordon, M. *Chem. Phys. Lett.* **1999**, *314*, 291-299.

9. Moral, M.; Muccioli, L.; Son, W.-J.; Olivier, Y.; Sancho-García, J. C. *J. Chem. Theory Comput.* **2015**, *11*, 168.

10. Tomasi, J.; Mennucci, B.; Cammi, R. *Chem. Rev.* **2005**, *105*, 2999-3093.

11. Mubarok, H.; Amin, A.; Lee, T.; Jung, J.; Lee, J.-H.; Lee, M. H. *Angew. Chem. Int. Ed.* **2023**, *62*, e202306879.

12. (a) Gorelsky, S. I.; Lever, A. B. P. *J. Organomet. Chem.* **2001**, *635*, 187. (b) Gorelsky, S. I. AOMix: Program for Molecular Orbital Analysis, http://www.sg-chem.net, version 6.94, 2019.

13. Martin, R. L. *J. Chem. Phys.* **2003**, *118*, 4775.

14. Lu, T.; Chen, F. *J. Comput. Chem.* **2012**, *33*, 580.

15. de Souza, B.; Farias, G.; Neese, F.; Izsak, R. *J. Chem. Theory Comput.* **2019**, *15*, 1896.

16. (a) Neese, F. *WIREs Comput. Mol. Sci.* **2011**, *2*, 73-78. (b) Neese, F. *WIREs Comput. Mol. Sci.* **2018**, *8*, e1327.

17. (a) Shizu, K.; Kaji, H. *Commun. Chem.* **2022**, *5*, 53. (b) Pershin, A.; Hall, D.; Lemaur, V.; Sancho-Garcia, J. C.; Muccioli, L.; Zysman-Colman, E.; Beljonne, D.; Olivier, Y. *Nat. Commun.* **2019**, *10*, 597.

18. Tahaoğlu, D.; Usta, H.; Alkan, F. *J. Phys. Chem. A* **2022**, *126*, 4199.

19. Pan, K.-C.; Li, S.-W.; Ho, Y.-Y.; Shiu, Y.-J.; Tsai, W.-L.; Jiao, M.; Lee, W.-K.; Wu, C.-C.; Chung, C.-L.; Chatterjee, T.; Li, Y.-S.; Wong, K.-T.; Hu, H.-C.; Chen, C.-C.; Lee, M.-T. *Adv. Funct. Mater.* **2016**, *26*, 7560.

Cartesian Coordinates of Optimized Compounds

- 2CB-BuDABNA **(1)**: S_0 (toluene) [electronic energy (au): -2654.6876]

H	0.000000	0.000000	5.517305
C	-0.140818	-1.209875	3.754799
H	-0.248060	-2.134700	4.318936
C	-0.124648	-1.216864	2.351325
N	-0.237139	-2.420735	1.657922
C	0.000000	0.000000	1.628979
B	0.000000	0.000000	0.112194
C	0.124648	1.216864	2.351325
N	0.237139	2.420735	1.657922
C	0.140818	1.209875	3.754799
H	0.248060	2.134700	4.318936
C	0.000000	0.000000	4.424172
C	-0.646590	4.338934	2.895685
H	-1.645740	3.968995	2.664944
C	-0.453241	5.502201	3.640988
C	0.869928	5.905859	3.893944
H	1.028906	6.813970	4.476049
C	1.983069	5.198536	3.434826
C	1.749073	4.033373	2.687471
H	2.576453	3.435516	2.299464
C	0.452201	3.616221	2.424600
C	0.073148	3.825629	-0.300517
H	0.274046	4.705249	0.309567
C	-0.197867	3.991810	-1.643129
H	-0.188483	5.000887	-2.056052
C	-0.496901	2.892922	-2.468812
C	-0.418596	1.624249	-1.899473
H	-0.627042	0.760654	-2.529450
C	-0.109409	1.398774	-0.544384
C	0.070834	2.539516	0.286164
C	-0.073148	-3.825629	-0.300517
H	-0.274046	-4.705249	0.309567
C	0.197867	-3.991810	-1.643129
H	0.188483	-5.000887	-2.056052
C	0.496901	-2.892922	-2.468812
C	0.418596	-1.624249	-1.899473
H	0.627042	-0.760654	-2.529450
C	0.109409	-1.398774	-0.544384
C	-0.070834	-2.539516	0.286164
C	-1.749073	-4.033373	2.687471
H	-2.576453	-3.435516	2.299464
C	-1.983069	-5.198536	3.434826
C	-0.869928	-5.905859	3.893944
H	-1.028906	-6.813970	4.476049
C	0.453241	-5.502201	3.640988
C	0.646590	-4.338934	2.895685
H	1.645740	-3.968995	2.664944
C	-0.452201	-3.616221	2.424600
C	-1.618429	6.338966	4.180787
C	-1.538330	7.756783	3.592569
C	-1.523457	6.412724	5.713069
C	-2.976222	5.738494	3.810119
H	-2.367731	8.374728	3.971912
H	-1.606370	7.732997	2.493820
H	-0.597840	8.260777	3.860995
H	-1.578389	5.408305	6.160887
H	-2.354130	7.012312	6.118142
H	-0.583906	6.879203	6.045071
H	-3.117825	5.684597	2.719698
H	-3.782795	6.367309	4.216757
H	-3.104851	4.726806	4.224703
C	3.423851	5.641237	3.712031
C	4.140967	5.890831	2.375539
C	4.152244	4.530922	4.486243
C	3.485122	6.926709	4.539757
H	5.181776	6.204652	2.553897
H	3.639725	6.685068	1.800774
H	4.168954	4.987528	1.747963
H	3.658999	4.333224	5.450649
H	5.193243	4.828099	4.690242
H	4.180300	3.586388	3.922660
H	3.002107	7.771246	4.024557
H	4.535872	7.204252	4.713539
H	3.008917	6.805067	5.524869
C	-3.423851	-5.641237	3.712031
C	-4.152244	-4.530922	4.486243
C	-3.485122	-6.926709	4.539757
C	-4.140967	-5.890831	2.375539
H	-5.193243	-4.828099	4.690242
H	-4.180300	-3.586388	3.922660
H	-3.658999	-4.333224	5.450649
H	-3.002107	-7.771246	4.024557
H	-4.535872	-7.204252	4.713539
H	-3.008917	-6.805067	5.524869
H	-4.168954	-4.987528	1.747963
H	-5.181776	-6.204652	2.553897
H	-3.639725	-6.685068	1.800774
C	1.618429	-6.338966	4.180787
C	1.523457	-6.412724	5.713069
C	2.976222	-5.738494	3.810119
C	1.538330	-7.756783	3.592569
H	2.354130	-7.012312	6.118142
H	0.583906	-6.879203	6.045071
H	1.578389	-5.408305	6.160887
H	3.117825	-5.684597	2.719698
H	3.782795	-6.367309	4.216757
H	3.104851	-4.726806	4.224703
H	0.597840	-8.260777	3.860995
H	2.367731	-8.374728	3.971912
H	1.606370	-7.732997	2.493820
B	-2.515890	4.366590	-5.733030
C	-2.436526	3.326248	-4.389969
B	0.211848	3.959847	-4.942567
C	-0.829828	3.082415	-3.919260
C	-3.497190	3.340579	-3.315732
B	-0.001847	3.255657	-6.560281
H	0.913614	3.225818	-7.337648
B	-1.344804	4.634762	-4.439692
B	-0.832461	4.771515	-6.125077
B	-1.683610	1.833574	-4.728048
B	0.000000	2.209465	-5.124941
B	-2.725797	2.625390	-5.913576
B	-1.690969	3.510489	-7.049814
B	-1.177054	1.922121	-6.419993
H	-2.029872	3.670349	-8.191316
H	0.855944	1.457182	-4.757956
H	-2.047059	0.886737	-4.098822
H	-3.813598	2.155339	-6.093473
H	-1.136145	0.923123	-7.083339
H	1.210209	4.402023	-4.449144
H	-1.490068	5.498720	-3.628482
H	-3.456593	5.107755	-5.787868
H	-0.540600	5.846526	-6.574115
H	-3.984573	4.322776	-3.273867
H	-3.054333	3.127609	-2.332829
H	-4.256777	2.577349	-3.526522
B	2.515890	-4.366590	-5.733030
C	2.436526	-3.326248	-4.389969
B	-0.211848	-3.959847	-4.942567
C	0.829828	-3.082415	-3.919260
C	3.497190	-3.340579	-3.315732
B	0.001847	-3.255657	-6.560281
H	-0.913614	-3.225818	-7.337648
B	1.344804	-4.634762	-4.439692
B	0.832461	-4.771515	-6.125077
B	1.683610	-1.833574	-4.728048
B	0.000000	-2.209465	-5.124941
B	2.725797	-2.625390	-5.913576
B	1.690969	-3.510489	-7.049814
B	1.177054	-1.922121	-6.419993
H	2.029872	-3.670349	-8.191316
H	-0.855944	-1.457182	-4.757956
H	2.047059	-0.886737	-4.098822
H	3.813598	-2.155339	-6.093473
H	1.136145	-0.923123	-7.083339
H	-1.210209	-4.402023	-4.449144
H	1.490068	-5.498720	-3.628482
H	3.456593	-5.107755	-5.787868
H	0.540600	-5.846526	-6.574115
H	3.984573	-4.322776	-3.273867
H	3.054333	-3.127609	-2.332829
H	4.256777	-2.577349	-3.526522

- 2CB-BuDABNA **(1)**: S_1 [= S_1-LE] (toluene) [electronic energy (au): -2654.5757]

H	0.000000	0.000000	5.564370
C	-0.109118	-1.199426	3.782845
H	-0.197304	-2.135425	4.331852
C	-0.099558	-1.199972	2.355402
N	-0.188283	-2.404077	1.689118
C	0.000000	0.000000	1.629234
B	0.000000	0.000000	0.089812
C	0.099558	1.199972	2.355402
N	0.188283	2.404077	1.689118
C	0.109118	1.199426	3.782845
H	0.197304	2.135425	4.331852
C	0.000000	0.000000	4.473086
C	-0.801573	4.286147	2.901022
H	-1.779056	3.893427	2.620795
C	-0.671530	5.445462	3.666282
C	0.628142	5.883295	3.977653
H	0.736976	6.789161	4.574440
C	1.779534	5.213971	3.557555
C	1.611161	4.050688	2.789734
H	2.470644	3.483424	2.426591
C	0.337514	3.600649	2.473538
C	0.136065	3.841517	-0.244170
H	0.269992	4.704719	0.405216
C	0.000000	4.051210	-1.615645
H	0.047492	5.068947	-2.001712
C	-0.189628	2.967383	-2.477495
C	-0.197607	1.674707	-1.936225
H	-0.355503	0.839864	-2.612523
C	-0.033092	1.401575	-0.554878
C	0.097313	2.546922	0.294194
C	-0.136065	-3.841517	-0.244170
H	-0.269992	-4.704719	0.405216
C	0.000000	-4.051210	-1.615645
H	-0.047492	-5.068947	-2.001712
C	0.189628	-2.967383	-2.477495
C	0.197607	-1.674707	-1.936225
H	0.355503	-0.839864	-2.612523
C	0.033092	-1.401575	-0.554878
C	-0.097313	-2.546922	0.294194
C	-1.611161	-4.050688	2.789734
H	-2.470644	-3.483424	2.426591
C	-1.779534	-5.213971	3.557555
C	-0.628142	-5.883295	3.977653
H	-0.736976	-6.789161	4.574440
C	0.671530	-5.445462	3.666282
C	0.801573	-4.286147	2.901022
H	1.779056	-3.893427	2.620795
C	-0.337514	-3.600649	2.473538
C	-1.881730	6.241335	4.166888
C	-1.814349	7.670183	3.604251
C	-1.855389	6.291769	5.702852
C	-3.204732	5.611115	3.726550
H	-2.677078	8.258410	3.955128
H	-1.832477	7.662473	2.503439
H	-0.901705	8.195580	3.922870
H	-1.905379	5.279100	6.132452
H	-2.717722	6.864197	6.079872
H	-0.943264	6.775287	6.083432
H	-3.295528	5.570161	2.630237
H	-4.045171	6.212447	4.104931
H	-3.325566	4.590410	4.121083
C	3.194318	5.696169	3.895387
C	3.952433	5.983190	2.589334
C	3.927017	4.599220	4.684479
C	3.183646	6.972718	4.738911
H	4.975652	6.326095	2.810289
H	3.448773	6.768609	2.004840
H	4.031311	5.088985	1.953127
H	3.403809	4.373702	5.626838
H	4.949022	4.926863	4.932580
H	4.008894	3.663769	4.110904
H	2.695411	7.808317	4.214277
H	4.217848	7.279815	4.956195
H	2.673817	6.825000	5.703476
C	-3.194318	-5.696169	3.895387
C	-3.927017	-4.599220	4.684479
C	-3.183646	-6.972718	4.738911
C	-3.952433	-5.983190	2.589334
H	-4.949022	-4.926863	4.932580
H	-4.008894	-3.663769	4.110904
H	-3.403809	-4.373702	5.626838
H	-2.695411	-7.808317	4.214277
H	-4.217848	-7.279815	4.956195
H	-2.673817	-6.825000	5.703476
H	-4.031311	-5.088985	1.953127
H	-4.975652	-6.326095	2.810289
H	-3.448773	-6.768609	2.004840
C	1.881730	-6.241335	4.166888
C	1.855389	-6.291769	5.702852
C	3.204732	-5.611115	3.726550
C	1.814349	-7.670183	3.604251
H	2.717722	-6.864197	6.079872
H	0.943264	-6.775287	6.083432
H	1.905379	-5.279100	6.132452
H	3.295528	-5.570161	2.630237
H	4.045171	-6.212447	4.104931
H	3.325566	-4.590410	4.121083
H	0.901705	-8.195580	3.922870
H	2.677078	-8.258410	3.955128
H	1.832477	-7.662473	2.503439

B	-1.911735	4.366944	-5.935418
C	-1.855529	3.229063	-4.668511
B	0.688713	4.337689	-4.729377
C	-0.325358	3.207868	-3.953866
C	-3.053982	2.975117	-3.785884
B	0.854239	3.766455	-6.401990
H	1.868939	3.962743	-7.015284
B	-1.021520	4.699345	-4.447810
B	-0.288966	5.076839	-6.011896
B	-0.805195	1.918562	-4.976689
B	0.823477	2.601102	-5.059229
B	-1.781630	2.638436	-6.260167
B	-0.758020	3.784620	-7.149194
B	-0.072037	2.251836	-6.550517
H	-0.941007	3.992393	-8.318539
H	1.731965	1.966632	-4.604224
H	-1.089633	0.864360	-4.498333
H	-2.727037	2.014254	-6.651532
H	0.246711	1.342096	-7.264853
H	1.512478	4.890700	-4.058586
H	-1.435642	5.440494	-3.607468
H	-2.950334	4.943485	-6.097631
H	-0.125045	6.222882	-6.332659
H	-3.802916	3.762069	-3.940679
H	-2.760129	2.975816	-2.727035
H	-3.506958	2.003814	-4.021093
B	1.911735	-4.366944	-5.935418
C	1.855529	-3.229063	-4.668511
B	-0.688713	-4.337689	-4.729377
C	0.325358	-3.207868	-3.953866
C	3.053982	-2.975117	-3.785884
B	-0.854239	-3.766455	-6.401990
H	-1.868939	-3.962743	-7.015284
B	1.021520	-4.699345	-4.447810
B	0.288966	-5.076839	-6.011896
B	0.805195	-1.918562	-4.976689
B	-0.823477	-2.601102	-5.059229
B	1.781630	-2.638436	-6.260167
B	0.758020	-3.784620	-7.149194
B	0.072037	-2.251836	-6.550517
H	0.941007	-3.992393	-8.318539
H	-1.731965	-1.966632	-4.604224
H	1.089633	-0.864360	-4.498333
H	2.727037	-2.014254	-6.651532
H	-0.246711	-1.342096	-7.264853
H	-1.512478	-4.890700	-4.058586
H	1.435642	-5.440494	-3.607468
H	2.950334	-4.943485	-6.097631
H	0.125045	-6.222882	-6.332659
H	3.802916	-3.762069	-3.940679
H	2.760129	-2.975816	-2.727035
H	3.506958	-2.003814	-4.021093

- 2CB-BuDABNA **(1)**: T_1 (toluene) [electronic energy (au): -2654.5924]

H	0.000000	0.000000	5.563937
C	0.104800	1.198706	3.773276
H	0.190234	2.136608	4.320632
C	0.096968	1.201625	2.356569
N	0.188769	2.412158	1.684523
C	0.000000	0.000000	1.614820
B	0.000000	0.000000	0.095713
C	-0.096968	-1.201625	2.356569
N	-0.188769	-2.412158	1.684523
C	-0.104800	-1.198706	3.773276
H	-0.190234	-2.136608	4.320632
C	0.000000	0.000000	4.473401
C	0.796986	-4.291671	2.903757
H	1.775450	-3.898675	2.627487
C	0.664275	-5.450631	3.668984
C	-0.636353	-5.888128	3.976362
H	-0.747281	-6.793706	4.573188
C	-1.786226	-5.218552	3.552447
C	-1.615168	-4.055723	2.784818
H	-2.473358	-3.487724	2.419869
C	-0.340557	-3.607220	2.471052
C	-0.136013	-3.847270	-0.246455
H	-0.267059	-4.710480	0.403627
C	0.000000	-4.056280	-1.615086
H	-0.043805	-5.073866	-2.001695
C	0.187146	-2.970181	-2.475011
C	0.190447	-1.678210	-1.932174
H	0.341199	-0.843758	-2.610555
C	0.027594	-1.404584	-0.554334
C	-0.099895	-2.550991	0.295810
C	0.136013	3.847270	-0.246455
H	0.267059	4.710480	0.403627
C	0.000000	4.056280	-1.615086
H	0.043805	5.073866	-2.001695
C	-0.187146	2.970181	-2.475011
C	-0.190447	1.678210	-1.932174
H	-0.341199	0.843758	-2.610555
C	-0.027594	1.404584	-0.554334
C	0.099895	2.550991	0.295810
C	1.615168	4.055723	2.784818
H	2.473358	3.487724	2.419869
C	1.786226	5.218552	3.552447
C	0.636353	5.888128	3.976362
H	0.747281	6.793706	4.573188
C	-0.664275	5.450631	3.668984
C	-0.796986	4.291671	2.903757
H	-1.775450	3.898675	2.627487
C	0.340557	3.607220	2.471052
C	1.872755	-6.246509	4.173798
C	1.806533	-7.676092	3.612943
C	1.841939	-6.294804	5.709753
C	3.197286	-5.617299	3.736589
H	2.667944	-8.264210	3.967225
H	1.828053	-7.669957	2.512156
H	0.892713	-8.200716	3.929484
H	1.890603	-5.281535	6.138020
H	2.703149	-6.866749	6.090066
H	0.928657	-6.777745	6.088274
H	3.291509	-5.578028	2.640480
H	4.036375	-6.218334	4.118444
H	3.317134	-4.596048	4.129939
C	-3.202182	-5.699881	3.886720
C	-3.958282	-5.984759	2.579044
C	-3.935106	-4.602986	4.675675
C	-3.194465	-6.977441	4.728754
H	-4.982096	-6.327255	2.797881
H	-3.454256	-6.769854	1.994378
H	-4.035707	-5.089649	1.943932
H	-3.413372	-4.379126	5.619209
H	-4.957998	-4.929687	4.921388
H	-4.014467	-3.666681	4.103176
H	-2.705725	-7.812861	4.204270
H	-4.229401	-7.283890	4.943465
H	-2.686670	-6.831236	5.694599
C	3.202182	5.699881	3.886720
C	3.935106	4.602986	4.675675
C	3.194465	6.977441	4.728754
C	3.958282	5.984759	2.579044
H	4.957998	4.929687	4.921388
H	4.014467	3.666681	4.103176
H	3.413372	4.379126	5.619209
H	2.705725	7.812861	4.204270
H	4.229401	7.283890	4.943465
H	2.686670	6.831236	5.694599
H	4.035707	5.089649	1.943932
H	4.982096	6.327255	2.797881
H	3.454256	6.769854	1.994378
C	-1.872755	6.246509	4.173798
C	-1.841939	6.294804	5.709753
C	-3.197286	5.617299	3.736589
C	-1.806533	7.676092	3.612943
H	-2.703149	6.866749	6.090066
H	-0.928657	6.777745	6.088274
H	-1.890603	5.281535	6.138020
H	-3.291509	5.578028	2.640480
H	-4.036375	6.218334	4.118444
H	-3.317134	4.596048	4.129939
H	-0.892713	8.200716	3.929484
H	-2.667944	8.264210	3.967225
H	-1.828053	7.669957	2.512156
B	1.910054	-4.365085	-5.933897
C	1.853539	-3.229660	-4.664203
B	-0.691036	-4.334739	-4.730037
C	0.325293	-3.207668	-3.952909
C	3.052606	-2.977881	-3.781296
B	-0.855161	-3.759769	-6.401350
H	-1.869813	-3.953247	-7.015383
B	1.018019	-4.700312	-4.448612
B	0.286072	-5.072783	-6.013464
B	0.806390	-1.916222	-4.972611
B	-0.823143	-2.597168	-5.056293
B	1.782100	-2.635898	-6.255930
B	0.757742	-3.779192	-7.147544
B	0.073605	-2.246345	-6.546803
H	0.941329	-3.984942	-8.317042
H	-1.730840	-1.962878	-4.599603
H	1.092339	-0.864013	-4.490906
H	2.729072	-2.012838	-6.644985
H	-0.243491	-1.334964	-7.259601
H	-1.515031	-4.886857	-4.058888
H	1.430261	-5.443087	-3.608957
H	2.948302	-4.942074	-6.096055
H	0.120644	-6.217931	-6.336218
H	3.786258	-3.783597	-3.910309
H	2.754221	-2.942908	-2.724411
H	3.526470	-2.022861	-4.040734
B	-1.910054	4.365085	-5.933897
C	-1.853539	3.229660	-4.664203
B	0.691036	4.334739	-4.730037
C	-0.325293	3.207668	-3.952909
C	-3.052606	2.977881	-3.781296
B	0.855161	3.759769	-6.401350
H	1.869813	3.953247	-7.015383
B	-1.018019	4.700312	-4.448612
B	-0.286072	5.072783	-6.013464
B	-0.806390	1.916222	-4.972611
B	0.823143	2.597168	-5.056293
B	-1.782100	2.635898	-6.255930
B	-0.757742	3.779192	-7.147544
B	-0.073605	2.246345	-6.546803
H	-0.941329	3.984942	-8.317042
H	1.730840	1.962878	-4.599603
H	-1.092339	0.864013	-4.490906
H	-2.729072	2.012838	-6.644985
H	0.243491	1.334964	-7.259601
H	1.515031	4.886857	-4.058888
H	-1.430261	5.443087	-3.608957
H	-2.948302	4.942074	-6.096055
H	-0.120644	6.217931	-6.336218
H	-3.786258	3.783597	-3.910309
H	-2.754221	2.942908	-2.724411
H	-3.526470	2.022861	-4.040734

- 2CB-BuDABNA **(1)**: T_2 (toluene) [electronic energy (au): -2654.5704]

H	0.000000	0.000000	5.557805
C	0.137099	1.207866	3.787797
H	0.242099	2.135629	4.347369
C	0.128153	1.210649	2.383166
N	0.246793	2.411077	1.676053
C	0.000000	0.000000	1.648817
B	0.000000	0.000000	0.110918
C	-0.128153	-1.210649	2.383166
N	-0.246793	-2.411077	1.676053
C	-0.137099	-1.207866	3.787797
H	-0.242099	-2.135629	4.347369
C	0.000000	0.000000	4.465381
C	0.680203	-4.319663	2.894279
H	1.670488	-3.950090	2.628161
C	0.512055	-5.476587	3.655651
C	-0.801563	-5.879785	3.954512
H	-0.940322	-6.783288	4.548750
C	-1.930342	-5.178571	3.525131
C	-1.723503	-4.019100	2.761066
H	-2.563797	-3.425653	2.395116
C	-0.435776	-3.605581	2.453315
C	-0.187205	-3.836112	-0.261272
H	-0.375220	-4.699539	0.374135
C	0.000000	-4.038604	-1.634437
H	-0.054101	-5.055072	-2.024192
C	0.246687	-2.966520	-2.479040
C	0.256409	-1.659648	-1.914251
H	0.467738	-0.821692	-2.573275
C	0.043192	-1.401131	-0.542131
C	-0.131058	-2.534489	0.301103
C	0.187205	3.836112	-0.261272
H	0.375220	4.699539	0.374135
C	0.000000	4.038604	-1.634437
H	0.054101	5.055072	-2.024192
C	-0.246687	2.966520	-2.479040
C	-0.256409	1.659648	-1.914251
H	-0.467738	0.821692	-2.573275
C	-0.043192	1.401131	-0.542131
C	0.131058	2.534489	0.301103
C	1.723503	4.019100	2.761066
H	2.563797	3.425653	2.395116
C	1.930342	5.178571	3.525131
C	0.801563	5.879785	3.954512
H	0.940322	6.783288	4.548750
C	-0.512055	5.476587	3.655651

C	-0.680203	4.319663	2.894279
H	-1.670488	3.950090	2.628161
C	0.435776	3.605581	2.453315
C	1.695811	-6.306264	4.164726
C	1.595056	-7.732273	3.600121
C	1.654613	-6.357307	5.700376
C	3.039353	-5.711572	3.737244
H	2.438216	-8.344216	3.958083
H	1.623454	-7.724279	2.499448
H	0.665545	-8.232906	3.909797
H	1.726496	-5.346570	6.131211
H	2.498435	-6.952266	6.084446
H	0.726778	-6.817327	6.071955
H	3.141378	-5.672206	2.641779
H	3.859758	-6.335740	4.122671
H	3.183635	-4.694805	4.133834
C	-3.360752	-5.622511	3.849473
C	-4.117279	-5.883617	2.537095
C	-4.067837	-4.508023	4.637376
C	-3.392738	-6.902007	4.687984
H	-5.151087	-6.199188	2.749476
H	-3.631370	-6.680502	1.952929
H	-4.167505	-4.984932	1.904257
H	-3.545643	-4.301521	5.584530
H	-5.101042	-4.806641	4.875769
H	-4.116603	-3.567923	4.067780
H	-2.923724	-7.749128	4.164178
H	-4.436840	-7.180798	4.896147
H	-2.886706	-6.771913	5.657011
C	3.360752	5.622511	3.849473
C	4.067837	4.508023	4.637376
C	3.392738	6.902007	4.687984
C	4.117279	5.883617	2.537095
H	5.101042	4.806641	4.875769
H	4.116603	3.567923	4.067780
H	3.545643	4.301521	5.584530
H	2.923724	7.749128	4.164178
H	4.436840	7.180798	4.896147
H	2.886706	6.771913	5.657011
H	4.167505	4.984932	1.904257
H	5.151087	6.199188	2.749476
H	3.631370	6.680502	1.952929
C	-1.695811	6.306264	4.164726
C	-1.654613	6.357307	5.700376
C	-3.039353	5.711572	3.737244
C	-1.595056	7.732273	3.600121
H	-2.498435	6.952266	6.084446
H	-0.726778	6.817327	6.071955
H	-1.726496	5.346570	6.131211
H	-3.141378	5.672206	2.641779
H	-3.859758	6.335740	4.122671
H	-3.183635	4.694805	4.133834
H	-0.665545	8.232906	3.909797
H	-2.438216	8.344216	3.958083
H	-1.623454	7.724279	2.499448
B	2.110962	-4.338450	-5.868317
C	1.995135	-3.202829	-4.602511
B	-0.534053	-4.337671	-4.763478
C	0.438780	-3.198752	-3.947401
C	3.156350	-2.940268	-3.673410
B	-0.641321	-3.764925	-6.439427
H	-1.629957	-3.970163	-7.090698
B	1.168143	-4.682968	-4.416355
B	0.499796	-5.064539	-6.007339
B	0.942178	-1.902108	-4.949112
B	-0.674624	-2.601793	-5.094695
B	1.976036	-2.610054	-6.193717
B	0.998807	-3.764899	-7.123843
B	0.274400	-2.240456	-6.550042
H	1.228518	-3.967882	-8.285543
H	-1.605735	-1.977672	-4.671898
H	1.194509	-0.845218	-4.458318
H	2.929177	-1.975647	-6.547887
H	-0.024989	-1.333002	-7.275093
H	-1.377669	-4.898675	-4.124996
H	1.554756	-5.419016	-3.558334
H	3.161402	-4.902783	-5.991254
H	0.359957	-6.211420	-6.335611
H	3.943603	-3.684580	-3.847622
H	2.837459	-3.011440	-2.624232
H	3.571864	-1.939402	-3.845181
B	-2.110962	4.338450	-5.868317
C	-1.995135	3.202829	-4.602511
B	0.534053	4.337671	-4.763478
C	-0.438780	3.198752	-3.947401
C	-3.156350	2.940268	-3.673410
B	0.641321	3.764925	-6.439427
H	1.629957	3.970163	-7.090698
B	-1.168143	4.682968	-4.416355
B	-0.499796	5.064539	-6.007339
B	-0.942178	1.902108	-4.949112
B	0.674624	2.601793	-5.094695
B	-1.976036	2.610054	-6.193717
B	-0.998807	3.764899	-7.123843
B	-0.274400	2.240456	-6.550042
H	-1.228518	3.967882	-8.285543
H	1.605735	1.977672	-4.671898
H	-1.194509	0.845218	-4.458318
H	-2.929177	1.975647	-6.547887
H	0.024989	1.333002	-7.275093
H	1.377669	4.898675	-4.124996
H	-1.554756	5.419016	-3.558334
H	-3.161402	4.902783	-5.991254
H	-0.359957	6.211420	-6.335611
H	-3.943603	3.684580	-3.847622
H	-2.837459	3.011440	-2.624232
H	-3.571864	1.939402	-3.845181

- 3CB-BuDABNA **(2)**: S_0 (toluene) [electronic energy (au): -2654.6862]

H	0.000000	0.000000	4.771994
C	-0.086075	-1.215254	3.010129
H	-0.150309	-2.143908	3.574499
C	-0.073784	-1.222328	1.606404
N	-0.140684	-2.429160	0.916247
C	0.000000	0.000000	0.882736
B	0.000000	0.000000	-0.631967
C	0.073784	1.222328	1.606404
N	0.140684	2.429160	0.916247
C	0.086075	1.215254	3.010129
H	0.150309	2.143908	3.574499
C	0.000000	0.000000	3.678810
C	-0.836837	4.323730	2.119643
H	-1.817275	3.917600	1.870347
C	-0.700454	5.506780	2.846447
C	0.601900	5.959938	3.122174
H	0.716111	6.884309	3.688863
C	1.748861	5.283896	2.700646
C	1.572080	4.099821	1.967556
H	2.428176	3.525982	1.606406
C	0.296404	3.633445	1.683017
C	-0.034093	3.832632	-1.034733
H	0.081900	4.700629	-0.391187
C	-0.245377	4.018240	-2.394756
C	-0.454908	2.895834	-3.213677
H	-0.680101	3.014544	-4.272928
C	-0.385261	1.630303	-2.658310
H	-0.570322	0.777928	-3.312626
C	-0.121887	1.395700	-1.293693
C	0.000000	2.541907	-0.462861
C	0.034093	-3.832632	-1.034733
H	-0.081900	-4.700629	-0.391187
C	0.245377	-4.018240	-2.394756
C	0.454908	-2.895834	-3.213677
H	0.680101	-3.014544	-4.272928
C	0.385261	-1.630303	-2.658310
H	0.570322	-0.777928	-3.312626
C	0.121887	-1.395700	-1.293693
C	0.000000	-2.541907	-0.462861
C	-1.572080	-4.099821	1.967556
H	-2.428176	-3.525982	1.606406
C	-1.748861	-5.283896	2.700646
C	-0.601900	-5.959938	3.122174
H	-0.716111	-6.884309	3.688863
C	0.700454	-5.506780	2.846447
C	0.836837	-4.323730	2.119643
H	1.817275	-3.917600	1.870347
C	-0.296404	-3.633445	1.683017
C	-1.905722	6.317681	3.334593
C	-1.853569	7.722147	2.711597
C	-1.855327	6.431560	4.866475
C	-3.232955	5.664999	2.942201
H	-2.714698	8.320817	3.048729
H	-1.885643	7.668606	1.612140
H	-0.939357	8.264530	2.995688
H	-1.893134	5.437198	5.338028
H	-2.714307	7.015548	5.233460
H	-0.939687	6.934324	5.212124
H	-3.343201	5.583511	1.849899
H	-4.069747	6.276192	3.312985
H	-3.340457	4.658907	3.376121
C	3.166876	5.782987	2.998711
C	3.901695	6.034608	1.672024
C	3.918784	4.714028	3.807755
C	3.164918	7.084850	3.802751
H	4.925237	6.393662	1.864613
H	3.380710	6.796539	1.071242
H	3.979092	5.119977	1.065025
H	3.413845	4.517184	4.766234
H	4.944447	5.051814	4.025883
H	3.990949	3.760430	3.263584
H	2.663658	7.901916	3.261462
H	4.201169	7.402707	3.993715
H	2.671645	6.964074	4.779486
C	-3.166876	-5.782987	2.998711
C	-3.918784	-4.714028	3.807755
C	-3.164918	-7.084850	3.802751
C	-3.901695	-6.034608	1.672024
H	-4.944447	-5.051814	4.025883
H	-3.990949	-3.760430	3.263584
H	-3.413845	-4.517184	4.766234
H	-2.663658	-7.901916	3.261462
H	-4.201169	-7.402707	3.993715
H	-2.671645	-6.964074	4.779486
H	-3.979092	-5.119977	1.065025
H	-4.925237	-6.393662	1.864613
H	-3.380710	-6.796539	1.071242
C	1.905722	-6.317681	3.334593
C	1.855327	-6.431560	4.866475
C	3.232955	-5.664999	2.942201
C	1.853569	-7.722147	2.711597
H	2.714307	-7.015548	5.233460
H	0.939687	-6.934324	5.212124
H	1.893134	-5.437198	5.338028
H	3.343201	-5.583511	1.849899
H	4.069747	-6.276192	3.312985
H	3.340457	-4.658907	3.376121
H	0.939357	-8.264530	2.995688
H	2.714698	-8.320817	3.048729
H	1.885643	-7.668606	1.612140
B	-0.716743	-7.173729	-4.847852
C	-1.070945	-6.172891	-3.515281
B	1.661552	-5.875864	-3.907864
C	0.323140	-5.411255	-2.961150
C	-2.380136	-5.425994	-3.417712
B	1.848859	-7.622289	-3.641299
H	2.936804	-8.130684	-3.675280
B	0.075578	-5.609704	-4.647199
B	1.032359	-7.021811	-5.107932
B	-0.355018	-6.703957	-2.058961
B	1.392926	-6.558731	-2.293233
B	-0.984172	-7.851809	-3.242281
B	0.375561	-8.426399	-4.227038
B	0.594834	-8.132109	-2.481824
H	0.369087	-9.532587	-4.695521
H	2.064387	-6.202695	-1.367339
H	-0.936272	-6.462370	-1.044856
H	-2.011312	-8.409774	-2.977102
H	0.748804	-9.005090	-1.671643
H	2.516668	-5.055719	-4.082438
H	-0.226447	-4.657000	-5.299804
H	-1.558266	-7.261073	-5.696726
H	1.505490	-7.086591	-6.209584
H	-2.825697	-5.312920	-4.413962
H	-2.225736	-4.425976	-2.989625
H	-3.082266	-5.973719	-2.776630
B	0.716743	7.173729	-4.847852
C	1.070945	6.172891	-3.515281
B	-1.661552	5.875864	-3.907864
C	-0.323140	5.411255	-2.961150
C	2.380136	5.425994	-3.417712
B	-1.848859	7.622289	-3.641299
H	-2.936804	8.130684	-3.675280
B	-0.075578	5.609704	-4.647199
B	-1.032359	7.021811	-5.107932
B	0.355018	6.703957	-2.058961
B	-1.392926	6.558731	-2.293233
B	0.984172	7.851809	-3.242281
B	-0.375561	8.426399	-4.227038
B	-0.594834	8.132109	-2.481824
H	-0.369087	9.532587	-4.695521
H	-2.064387	6.202695	-1.367339
H	0.936272	6.462370	-1.044856
H	2.011312	8.409774	-2.977102
H	-0.748804	9.005090	-1.671643
H	-2.516668	5.055719	-4.082438
H	0.226447	4.657000	-5.299804
H	1.558266	7.261073	-5.696726

H	-1.505490	7.086591	-6.209584
H	2.825697	5.312920	-4.413962
H	2.225736	4.425976	-2.989625
H	3.082266	5.973719	-2.776630

- 3CB-BuDABNA **(2)**: S_1 (toluene) [electronic energy (au): -2654.5827]

H	0.000000	0.000000	4.770092
C	-0.040735	-1.205411	2.992996
H	-0.073315	-2.144131	3.543515
C	-0.032693	-1.207414	1.561858
N	-0.054907	-2.410610	0.899219
C	0.000000	0.000000	0.844120
B	0.000000	0.000000	-0.696820
C	0.032693	1.207414	1.561858
N	0.054907	2.410610	0.899219
C	0.040735	1.205411	2.992996
H	0.073315	2.144131	3.543515
C	0.000000	0.000000	3.678664
C	-1.027055	4.262154	2.079520
H	-1.984309	3.824293	1.796523
C	-0.953781	5.444692	2.816480
C	0.323091	5.944744	3.128296
H	0.387237	6.870133	3.700881
C	1.506071	5.316201	2.734060
C	1.394953	4.129638	1.991732
H	2.281338	3.593586	1.646036
C	0.144395	3.618215	1.675943
C	-0.086814	3.837583	-1.038470
H	-0.014245	4.695436	-0.375701
C	-0.242697	4.055746	-2.418729
C	-0.396568	2.930979	-3.247523
H	-0.576363	3.058840	-4.315649
C	-0.334652	1.653710	-2.718150
H	-0.485722	0.815802	-3.398138
C	-0.121261	1.387999	-1.340472
C	-0.049187	2.545644	-0.503126
C	0.086814	-3.837583	-1.038470
H	0.014245	-4.695436	-0.375701
C	0.242697	-4.055746	-2.418729
C	0.396568	-2.930979	-3.247523
H	0.576363	-3.058840	-4.315649
C	0.334652	-1.653710	-2.718150
H	0.485722	-0.815802	-3.398138
C	0.121261	-1.387999	-1.340472
C	0.049187	-2.545644	-0.503126
C	-1.394953	-4.129638	1.991732
H	-2.281338	-3.593586	1.646036
C	-1.506071	-5.316201	2.734060
C	-0.323091	-5.944744	3.128296
H	-0.387237	-6.870133	3.700881
C	0.953781	-5.444692	2.816480
C	1.027055	-4.262154	2.079520
H	1.984309	-3.824293	1.796523
C	-0.144395	-3.618215	1.675943
C	-2.201339	6.206379	3.276032
C	-2.184248	7.615095	2.660463
C	-2.196214	6.313225	4.809249
C	-3.491816	5.506690	2.843992
H	-3.077525	8.177864	2.974625
H	-2.181603	7.566296	1.560458
H	-1.300811	8.191020	2.974262
H	-2.211532	5.315639	5.275443
H	-3.085442	6.863779	5.154917
H	-1.309434	6.846974	5.182628
H	-3.569191	5.427362	1.748765
H	-4.360533	6.084387	3.194177
H	-3.574421	4.495125	3.270902
C	2.895571	5.869747	3.067486
C	3.647638	6.158954	1.758139
C	3.672291	4.827049	3.887209
C	2.822130	7.165132	3.878491
H	4.650734	6.559049	1.975226
H	3.108585	6.901600	1.149442
H	3.776380	5.251973	1.148412
H	3.155250	4.603630	4.833455
H	4.677771	5.205611	4.130234
H	3.797560	3.881290	3.338817
H	2.301184	7.964779	3.330012
H	3.840094	7.523093	4.094675
H	2.312173	7.018439	4.843088
C	-2.895571	-5.869747	3.067486
C	-3.672291	-4.827049	3.887209
C	-2.822130	-7.165132	3.878491
C	-3.647638	-6.158954	1.758139
H	-4.677771	-5.205611	4.130234
H	-3.797560	-3.881290	3.338817
H	-3.155250	-4.603630	4.833455
H	-2.301184	-7.964779	3.330012
H	-3.840094	-7.523093	4.094675
H	-2.312173	-7.018439	4.843088
H	-3.776380	-5.251973	1.148412
H	-4.650734	-6.559049	1.975226
H	-3.108585	-6.901600	1.149442
C	2.201339	-6.206379	3.276032
C	2.196214	-6.313225	4.809249
C	3.491816	-5.506690	2.843992
C	2.184248	-7.615095	2.660463
H	3.085442	-6.863779	5.154917
H	1.309434	-6.846974	5.182628
H	2.211532	-5.315639	5.275443
H	3.569191	-5.427362	1.748765
H	4.360533	-6.084387	3.194177
H	3.574421	-4.495125	3.270902
H	1.300811	-8.191020	2.974262
H	3.077525	-8.177864	2.974625
H	2.181603	-7.566296	1.560458
B	-0.772076	-7.268370	-4.791701
C	-1.108383	-6.261485	-3.470973
B	1.622430	-5.918590	-3.951937
C	0.325567	-5.439745	-2.961995
C	-2.414119	-5.519566	-3.338634
B	1.838653	-7.658804	-3.664415
H	2.931474	-8.155195	-3.727009
B	0.000000	-5.684047	-4.627907
B	0.964964	-7.089257	-5.109584
B	-0.325395	-6.729356	-2.034122
B	1.416882	-6.577382	-2.319483
B	-0.976999	-7.922107	-3.167833
B	0.359669	-8.492628	-4.185715
B	0.631209	-8.159613	-2.452910
H	0.356824	-9.607254	-4.634996
H	2.114116	-6.207142	-1.417602
H	-0.873825	-6.482841	-1.001963
H	-1.980898	-8.496746	-2.851496
H	0.823940	-9.016410	-1.632911
H	2.463442	-5.095822	-4.178368
H	-0.336080	-4.750734	-5.293182
H	-1.632670	-7.381519	-5.618851
H	1.400869	-7.166621	-6.226187
H	-2.908300	-5.443318	-4.315478
H	-2.237096	-4.503014	-2.958258
H	-3.082342	-6.041361	-2.641804
B	0.772076	7.268370	-4.791701
C	1.108383	6.261485	-3.470973
B	-1.622430	5.918590	-3.951937
C	-0.325567	5.439745	-2.961995
C	2.414119	5.519566	-3.338634
B	-1.838653	7.658804	-3.664415
H	-2.931474	8.155195	-3.727009
B	0.000000	5.684047	-4.627907
B	-0.964964	7.089257	-5.109584
B	0.325395	6.729356	-2.034122
B	-1.416882	6.577382	-2.319483
B	0.976999	7.922107	-3.167833
B	-0.359669	8.492628	-4.185715
B	-0.631209	8.159613	-2.452910
H	-0.356824	9.607254	-4.634996
H	-2.114116	6.207142	-1.417602
H	0.873825	6.482841	-1.001963
H	1.980898	8.496746	-2.851496
H	-0.823940	9.016410	-1.632911
H	-2.463442	5.095822	-4.178368
H	0.336080	4.750734	-5.293182
H	1.632670	7.381519	-5.618851
H	-1.400869	7.166621	-6.226187
H	2.908300	5.443318	-4.315478
H	2.237096	4.503014	-2.958258
H	3.082342	6.041361	-2.641804

- 3CB-BuDABNA **(2)**: T_1 (toluene) [electronic energy (au): -2654.5988]

H	0.000000	0.000000	4.768759
C	-0.043577	-1.204898	2.985811
H	-0.079202	-2.143996	3.536465
C	-0.036838	-1.207408	1.567404
N	-0.067886	-2.419350	0.894988
C	0.000000	0.000000	0.838303
B	0.000000	0.000000	-0.688276
C	0.036838	1.207408	1.567404
N	0.067886	2.419350	0.894988
C	0.043577	1.204898	2.985811
H	0.079202	2.143996	3.536465
C	0.000000	0.000000	3.677685
C	-0.995755	4.274578	2.086394
H	-1.957457	3.843104	1.808855
C	-0.910587	5.455494	2.824498
C	0.371113	5.946260	3.130739
H	0.444496	6.870218	3.704533
C	1.547672	5.309985	2.729633
C	1.424646	4.125706	1.985910
H	2.305611	3.583462	1.636169
C	0.169121	3.624370	1.674303
C	-0.072122	3.843764	-1.039376
H	0.002557	4.701456	-0.376378
C	-0.228228	4.060043	-2.417047
C	-0.386825	2.932568	-3.241813
H	-0.568156	3.058071	-4.310069
C	-0.326937	1.655119	-2.711307
H	-0.479626	0.818185	-3.392037
C	-0.112608	1.388699	-1.335992
C	-0.036711	2.550495	-0.498274
C	0.072122	-3.843764	-1.039376
H	-0.002557	-4.701456	-0.376378
C	0.228228	-4.060043	-2.417047
C	0.386825	-2.932568	-3.241813
H	0.568156	-3.058071	-4.310069
C	0.326937	-1.655119	-2.711307
H	0.479626	-0.818185	-3.392037
C	0.112608	-1.388699	-1.335992
C	0.036711	-2.550495	-0.498274
C	-1.424646	-4.125706	1.985910
H	-2.305611	-3.583462	1.636169
C	-1.547672	-5.309985	2.729633
C	-0.371113	-5.946260	3.130739
H	-0.444496	-6.870218	3.704533
C	0.910587	-5.455494	2.824498
C	0.995755	-4.274578	2.086394
H	1.957457	-3.843104	1.808855
C	-0.169121	-3.624370	1.674303
C	-2.150587	6.225174	3.291238
C	-2.126326	7.635213	2.679033
C	-2.137606	6.328258	4.824664
C	-3.447998	5.535705	2.863598
H	-3.013885	8.203746	2.998979
H	-2.129583	7.589119	1.578888
H	-1.237132	8.203895	2.989776
H	-2.157609	5.329667	5.288455
H	-3.021359	6.884161	5.175789
H	-1.245339	6.854874	5.195072
H	-3.531001	5.459583	1.768540
H	-4.310951	6.118716	3.219198
H	-3.535731	4.523717	3.288404
C	2.942647	5.852771	3.057919
C	3.692448	6.136734	1.746161
C	3.714007	4.803597	3.874453
C	2.882208	7.148400	3.869618
H	4.699336	6.528974	1.960010
H	3.157151	6.883871	1.139602
H	3.812241	5.229018	1.135700
H	3.198447	4.583722	4.822298
H	4.723302	5.174112	4.114103
H	3.829621	3.857052	3.325330
H	2.365106	7.952165	3.323485
H	3.903681	7.498572	4.081981
H	2.374964	7.005207	4.836153
C	-2.942647	-5.852771	3.057919
C	-3.714007	-4.803597	3.874453
C	-2.882208	-7.148400	3.869618
C	-3.692448	-6.136734	1.746161
H	-4.723302	-5.174112	4.114103
H	-3.829621	-3.857052	3.325330
H	-3.198447	-4.583722	4.822298
H	-2.365106	-7.952165	3.323485
H	-3.903681	-7.498572	4.081981
H	-2.374964	-7.005207	4.836153
H	-3.812241	-5.229018	1.135700
H	-4.699336	-6.528974	1.960010
H	-3.157151	-6.883871	1.139602
C	2.150587	-6.225174	3.291238
C	2.137606	-6.328258	4.824664
C	3.447998	-5.535705	2.863598
C	2.126326	-7.635213	2.679033
H	3.021359	-6.884161	5.175789
H	1.245339	-6.854874	5.195072

H	2.157609	-5.329667	5.288455
H	3.531001	-5.459583	1.768540
H	4.310951	-6.118716	3.219198
H	3.535731	-4.523717	3.288404
H	1.237132	-8.203895	2.989776
H	3.013885	-8.203746	2.998979
H	2.129583	-7.589119	1.578888
B	-0.777644	-7.262091	-4.801505
C	-1.114778	-6.253864	-3.479273
B	1.615046	-5.923212	-3.945254
C	0.309647	-5.445266	-2.963510
C	-2.421210	-5.510904	-3.353375
B	1.824942	-7.664586	-3.659945
H	2.916538	-8.164052	-3.716953
B	0.000000	-5.682165	-4.633611
B	0.961942	-7.089924	-5.109561
B	-0.345131	-6.734091	-2.038146
B	1.397866	-6.584417	-2.315602
B	-0.993909	-7.918658	-3.180127
B	0.345945	-8.492074	-4.191970
B	0.608808	-8.164814	-2.457032
H	0.341095	-9.605586	-4.643731
H	2.091396	-6.215857	-1.410351
H	-0.899273	-6.485992	-1.009546
H	-2.003108	-8.488748	-2.873090
H	0.793951	-9.023994	-1.637946
H	2.458985	-5.100993	-4.162330
H	-0.329219	-4.745478	-5.297368
H	-1.635656	-7.370928	-5.631731
H	1.404384	-7.166435	-6.223548
H	-2.902049	-5.418737	-4.335420
H	-2.248511	-4.501105	-2.954067
H	-3.099483	-6.043174	-2.674476
B	0.777644	7.262091	-4.801505
C	1.114778	6.253864	-3.479273
B	-1.615046	5.923212	-3.945254
C	-0.309647	5.445266	-2.963510
C	2.421210	5.510904	-3.353375
B	-1.824942	7.664586	-3.659945
H	-2.916538	8.164052	-3.716953
B	0.000000	5.682165	-4.633611
B	-0.961942	7.089924	-5.109561
B	0.345131	6.734091	-2.038146
B	-1.397866	6.584417	-2.315602
B	0.993909	7.918658	-3.180127
B	-0.345945	8.492074	-4.191970
B	-0.608808	8.164814	-2.457032
H	-0.341095	9.605586	-4.643731
H	-2.091396	6.215857	-1.410351
H	0.899273	6.485992	-1.009546
H	2.003108	8.488748	-2.873090
H	-0.793951	9.023994	-1.637946
H	-2.458985	5.100993	-4.162330
H	0.329219	4.745478	-5.297368
H	1.635656	7.370928	-5.631731
H	-1.404384	7.166435	-6.223548
H	2.902049	5.418737	-4.335420
H	2.248511	4.501105	-2.954067
H	3.099483	6.043174	-2.674476

- 3CB-BuDABNA **(2)**: T_2 (toluene) [electronic energy (au): -2654.5754]

H	0.000000	0.000000	4.775499
C	-0.069766	-1.213479	3.007418
H	-0.121712	-2.145598	3.567483
C	-0.062683	-1.214554	1.604245
N	-0.119386	-2.425146	0.898575
C	0.000000	0.000000	0.880242
B	0.000000	0.000000	-0.664989
C	0.062683	1.214554	1.604245
N	0.119386	2.425146	0.898575
C	0.069766	1.213479	3.007418
H	0.121712	2.145598	3.567483
C	0.000000	0.000000	3.683058
C	-0.892391	4.301781	2.099801
H	-1.865902	3.891152	1.832091
C	-0.774194	5.478655	2.839975
C	0.520716	5.938868	3.137723
H	0.619785	6.859124	3.713683
C	1.679033	5.277080	2.725055
C	1.522961	4.098367	1.978554
H	2.388401	3.535609	1.622730
C	0.253953	3.628074	1.673410
C	-0.022791	3.841024	-1.034943
H	0.085594	4.700632	-0.379133
C	-0.204752	4.054735	-2.423503
C	-0.405559	2.932582	-3.229559
H	-0.609631	3.048871	-4.294621
C	-0.360622	1.647564	-2.680990
H	-0.554888	0.808384	-3.349671
C	-0.116345	1.388826	-1.312437
C	-0.004043	2.543773	-0.480611
C	0.022791	-3.841024	-1.034943
H	-0.085594	-4.700632	-0.379133
C	0.204752	-4.054735	-2.423503
C	0.405559	-2.932582	-3.229559
H	0.609631	-3.048871	-4.294621
C	0.360622	-1.647564	-2.680990
H	0.554888	-0.808384	-3.349671
C	0.116345	-1.388826	-1.312437
C	0.004043	-2.543773	-0.480611
C	-1.522961	-4.098367	1.978554
H	-2.388401	-3.535609	1.622730
C	-1.679033	-5.277080	2.725055
C	-0.520716	-5.938868	3.137723
H	-0.619785	-6.859124	3.713683
C	0.774194	-5.478655	2.839975
C	0.892391	-4.301781	2.099801
H	1.865902	-3.891152	1.832091
C	-0.253953	-3.628074	1.673410
C	-1.992446	6.276477	3.316741
C	-1.940317	7.686623	2.706689
C	-1.965368	6.376576	4.850212
C	-3.308697	5.617850	2.898277
H	-2.811334	8.275610	3.035107
H	-1.954121	7.642619	1.606497
H	-1.035120	8.233355	3.010533
H	-2.004089	5.377861	5.312418
H	-2.833599	6.951760	5.209033
H	-1.058519	6.882253	5.214283
H	-3.401935	5.545766	1.803745
H	-4.155233	6.219871	3.261636
H	-3.415976	4.607309	3.321912
C	3.088721	5.786832	3.043192
C	3.835345	6.056282	1.726609
C	3.841359	4.718076	3.851840
C	3.064659	7.081848	3.857782
H	4.852802	6.424141	1.933804
H	3.313416	6.817424	1.125786
H	3.929233	5.147424	1.113288
H	3.327705	4.508228	4.802915
H	4.860965	5.064166	4.084476
H	3.929860	3.769994	3.300404
H	2.562171	7.898563	3.317190
H	4.095568	7.407752	4.063154
H	2.561521	6.948390	4.827825
C	-3.088721	-5.786832	3.043192
C	-3.841359	-4.718076	3.851840
C	-3.064659	-7.081848	3.857782
C	-3.835345	-6.056282	1.726609
H	-4.860965	-5.064166	4.084476
H	-3.929860	-3.769994	3.300404
H	-3.327705	-4.508228	4.802915
H	-2.562171	-7.898563	3.317190
H	-4.095568	-7.407752	4.063154
H	-2.561521	-6.948390	4.827825
H	-3.929233	-5.147424	1.113288
H	-4.852802	-6.424141	1.933804
H	-3.313416	-6.817424	1.125786
C	1.992446	-6.276477	3.316741
C	1.965368	-6.376576	4.850212
C	3.308697	-5.617850	2.898277
C	1.940317	-7.686623	2.706689
H	2.833599	-6.951760	5.209033
H	1.058519	-6.882253	5.214283
H	2.004089	-5.377861	5.312418
H	3.401935	-5.545766	1.803745
H	4.155233	-6.219871	3.261636
H	3.415976	-4.607309	3.321912
H	1.035120	-8.233355	3.010533
H	2.811334	-8.275610	3.035107
H	1.954121	-7.642619	1.606497
B	-0.806443	-7.227638	-4.845626
C	-1.161122	-6.216546	-3.531458
B	1.590767	-5.943844	-3.918039
C	0.270028	-5.437841	-2.971801
C	-2.453004	-5.444035	-3.441268
B	1.753024	-7.689612	-3.631187
H	2.834323	-8.213282	-3.657858
B	0.000000	-5.664771	-4.649337
B	0.944551	-7.092425	-5.103203
B	-0.442035	-6.713618	-2.070523
B	1.311845	-6.602872	-2.296632
B	-1.084016	-7.882533	-3.232840
B	0.272013	-8.483107	-4.207027
B	0.492023	-8.164687	-2.464262
H	0.255954	-9.595239	-4.661700
H	1.985997	-6.251881	-1.369730
H	-1.019152	-6.454571	-1.057141
H	-2.113198	-8.431552	-2.955354
H	0.634718	-9.029047	-1.642327
H	2.458348	-5.139819	-4.108784
H	-0.288946	-4.719080	-5.319449
H	-1.641621	-7.315378	-5.701214
H	1.416215	-7.176130	-6.204559
H	-2.912108	-5.355348	-4.433999
H	-2.266250	-4.432256	-3.053240
H	-3.156382	-5.951175	-2.768748
B	0.806443	7.227638	-4.845626
C	1.161122	6.216546	-3.531458
B	-1.590767	5.943844	-3.918039
C	-0.270028	5.437841	-2.971801
C	2.453004	5.444035	-3.441268
B	-1.753024	7.689612	-3.631187
H	-2.834323	8.213282	-3.657858
B	0.000000	5.664771	-4.649337
B	-0.944551	7.092425	-5.103203
B	0.442035	6.713618	-2.070523
B	-1.311845	6.602872	-2.296632
B	1.084016	7.882533	-3.232840
B	-0.272013	8.483107	-4.207027
B	-0.492023	8.164687	-2.464262
H	-0.255954	9.595239	-4.661700
H	-1.985997	6.251881	-1.369730
H	1.019152	6.454571	-1.057141
H	2.113198	8.431552	-2.955354
H	-0.634718	9.029047	-1.642327
H	-2.458348	5.139819	-4.108784
H	0.288946	4.719080	-5.319449
H	1.641621	7.315378	-5.701214
H	-1.416215	7.176130	-6.204559
H	2.912108	5.355348	-4.433999
H	2.266250	4.432256	-3.053240
H	3.156382	5.951175	-2.768748

- BuDABNA **(3)**: S_0 (toluene) [electronic energy (au): -1915.6560]

H	0.000000	0.000000	3.105325
C	0.579524	-1.071324	1.343083
H	1.022538	-1.889529	1.908127
C	0.596291	-1.069132	-0.062106
N	1.191724	-2.119658	-0.753293
C	0.000000	0.000000	-0.784649
B	0.000000	0.000000	-2.302638
C	-0.596291	1.069132	-0.062106
N	-1.191724	2.119658	-0.753293
C	-0.579524	1.071324	1.343083
H	-1.022538	1.889529	1.908127
C	0.000000	0.000000	2.011905
C	-3.018818	3.174774	0.488139
H	-3.622598	2.298505	0.251791
C	-3.530026	4.233059	1.239545
C	-2.681037	5.324159	1.497411
H	-3.073393	6.155468	2.083883
C	-1.364092	5.388156	1.037160
C	-0.885439	4.304000	0.284907
H	0.135088	4.292077	-0.103624
C	-1.704359	3.216374	0.016029
C	-2.118926	3.196978	-2.710116
H	-2.486439	4.008570	-2.083069
C	-2.397154	3.208302	-4.066008
H	-2.974811	4.037218	-4.483706
C	-1.966420	2.162737	-4.889800
H	-2.216805	2.152646	-5.952973
C	-1.216019	1.140855	-4.331513
H	-0.899349	0.318950	-4.974758
C	-0.868564	1.100155	-2.963482
C	-1.384081	2.135018	-2.134527
C	2.118926	-3.196978	-2.710116
H	2.486439	-4.008570	-2.083069
C	2.397154	-3.208302	-4.066008
H	2.974811	-4.037218	-4.483706
C	1.966420	-2.162737	-4.889800
H	2.216805	-2.152646	-5.952973
C	1.216019	-1.140855	-4.331513

H	0.899349	-0.318950	-4.974758
C	0.868564	-1.100155	-2.963482
C	1.384081	-2.135018	-2.134527
C	0.885439	-4.304000	0.284907
H	-0.135088	-4.292077	-0.103624
C	1.364092	-5.388156	1.037160
C	2.681037	-5.324159	1.497411
H	3.073393	-6.155468	2.083883
C	3.530026	-4.233059	1.239545
C	3.018818	-3.174774	0.488139
H	3.622598	-2.298505	0.251791
C	1.704359	-3.216374	0.016029
C	-4.963554	4.242107	1.782602
C	-5.716306	5.452838	1.208110
C	-4.924970	4.341646	3.315921
C	-5.729667	2.973419	1.401439
H	-6.749633	5.476471	1.589698
H	-5.760191	5.404448	0.108947
H	-5.237451	6.404173	1.484468
H	-4.391730	3.483318	3.753371
H	-5.948364	4.351031	3.723989
H	-4.422368	5.259393	3.656282
H	-5.814905	2.857384	0.310077
H	-6.750778	3.020390	1.809782
H	-5.252434	2.068147	1.807066
C	-0.440254	6.578377	1.318591
C	0.000000	7.201555	-0.015770
C	0.796131	6.086825	2.088235
C	-1.128074	7.661281	2.152637
H	0.671685	8.056090	0.164572
H	-0.868494	7.565075	-0.586797
H	0.539952	6.480876	-0.647838
H	0.508123	5.636860	3.051076
H	1.476989	6.928077	2.294726
H	1.361109	5.332496	1.520584
H	-2.010387	8.076000	1.641193
H	-0.428040	8.492218	2.328931
H	-1.445707	7.282842	3.136483
C	0.440254	-6.578377	1.318591
C	-0.796131	-6.086825	2.088235
C	1.128074	-7.661281	2.152637
C	0.000000	-7.201555	-0.015770
H	-1.476989	-6.928077	2.294726
H	-1.361109	-5.332496	1.520584
H	-0.508123	-5.636860	3.051076
H	2.010387	-8.076000	1.641193
H	0.428040	-8.492218	2.328931
H	1.445707	-7.282842	3.136483
H	-0.539952	-6.480876	-0.647838
H	-0.671685	-8.056090	0.164572
H	0.868494	-7.565075	-0.586797
C	4.963554	-4.242107	1.782602
C	4.924970	-4.341646	3.315921
C	5.729667	-2.973419	1.401439
C	5.716306	-5.452838	1.208110
H	5.948364	-4.351031	3.723989
H	4.422368	-5.259393	3.656282
H	4.391730	-3.483318	3.753371
H	5.814905	-2.857384	0.310077
H	6.750778	-3.020390	1.809782
H	5.252434	-2.068147	1.807066
H	5.237451	-6.404173	1.484468
H	6.749633	-5.476471	1.589698
H	5.760191	-5.404448	0.108947

- BuDABNA **(3)**: S_1 (toluene) [electronic energy (au): -1915.5403]

H	0.000000	0.000000	3.106637
C	0.019255	-1.205479	1.325696
H	0.031105	-2.144585	1.876556
C	0.028630	-1.205882	-0.101639
N	0.068139	-2.412266	-0.770920
C	0.000000	0.000000	-0.824178
B	0.000000	0.000000	-2.364135
C	-0.028630	1.205882	-0.101639
N	-0.068139	2.412266	-0.770920
C	-0.019255	1.205479	1.325696
H	-0.031105	2.144585	1.876556
C	0.000000	0.000000	2.015131
C	-1.251488	4.179041	0.439962
H	-2.181046	3.684474	0.158029
C	-1.245645	5.343754	1.207938
C	0.000000	5.916978	1.520678
H	0.012116	6.827533	2.120305
C	1.215439	5.374031	1.098646
C	1.170897	4.201264	0.328084
H	2.084906	3.727490	-0.035990
C	-0.046977	3.616573	0.011030
C	-0.291101	3.837845	-2.710336
H	-0.252600	4.708815	-2.057422
C	-0.458044	4.025626	-4.087007
H	-0.538094	5.038136	-4.488719
C	-0.535585	2.914062	-4.923423
H	-0.692978	3.043028	-5.997807
C	-0.410258	1.632902	-4.391291
H	-0.502872	0.783528	-5.067626
C	-0.196084	1.384406	-3.011647
C	-0.183448	2.546741	-2.171655
C	0.291101	-3.837845	-2.710336
H	0.252600	-4.708815	-2.057422
C	0.458044	-4.025626	-4.087007
H	0.538094	-5.038136	-4.488719
C	0.535585	-2.914062	-4.923423
H	0.692978	-3.043028	-5.997807
C	0.410258	-1.632902	-4.391291
H	0.502872	-0.783528	-5.067626
C	0.196084	-1.384406	-3.011647
C	0.183448	-2.546741	-2.171655
C	-1.170897	-4.201264	0.328084
H	-2.084906	-3.727490	-0.035990
C	-1.215439	-5.374031	1.098646
C	0.000000	-5.916978	1.520678
H	-0.012116	-6.827533	2.120305
C	1.245645	-5.343754	1.207938
C	1.251488	-4.179041	0.439962
H	2.181046	-3.684474	0.158029
C	0.046977	-3.616573	0.011030
C	-2.533798	6.004930	1.710957
C	-2.622042	7.432931	1.149042
C	-2.511780	6.057595	3.246910
C	-3.782269	5.236393	1.272340
H	-3.543021	7.924864	1.500560
H	-2.639839	7.423091	0.048224
H	-1.770921	8.053571	1.466952
H	-2.452033	5.045094	3.675705
H	-3.430287	6.533789	3.625534
H	-1.656267	6.635797	3.627056
H	-3.869783	5.185948	0.176228
H	-4.682001	5.743552	1.652750
H	-3.791309	4.208300	1.665914
C	2.571636	6.001536	1.439263
C	3.297726	6.369574	0.135389
C	3.414494	4.986171	2.227440
C	2.426922	7.268101	2.285555
H	4.279419	6.817117	0.358418
H	2.715015	7.099066	-0.448199
H	3.469769	5.489880	-0.502519
H	2.916630	4.706266	3.168962
H	4.396983	5.417688	2.476816
H	3.592384	4.064781	1.653024
H	1.853712	8.048909	1.762400
H	3.423187	7.681443	2.504739
H	1.934455	7.065206	3.249112
C	-2.571636	-6.001536	1.439263
C	-3.414494	-4.986171	2.227440
C	-2.426922	-7.268101	2.285555
C	-3.297726	-6.369574	0.135389
H	-4.396983	-5.417688	2.476816
H	-3.592384	-4.064781	1.653024
H	-2.916630	-4.706266	3.168962
H	-1.853712	-8.048909	1.762400
H	-3.423187	-7.681443	2.504739
H	-1.934455	-7.065206	3.249112
H	-3.469769	-5.489880	-0.502519
H	-4.279419	-6.817117	0.358418
H	-2.715015	-7.099066	-0.448199
C	2.533798	-6.004930	1.710957
C	2.511780	-6.057595	3.246910
C	3.782269	-5.236393	1.272340
C	2.622042	-7.432931	1.149042
H	3.430287	-6.533789	3.625534
H	1.656267	-6.635797	3.627056
H	2.452033	-5.045094	3.675705
H	3.869783	-5.185948	0.176228
H	4.682001	-5.743552	1.652750
H	3.791309	-4.208300	1.665914
H	1.770921	-8.053571	1.466952
H	3.543021	-7.924864	1.500560
H	2.639839	-7.423091	0.048224

- BuDABNA **(3)**: T_1 (toluene) [electronic energy (au): -1915.5590]

H	0.000000	0.000000	3.109207
C	0.028757	-1.204747	1.317601
H	0.047226	-2.145619	1.866609
C	0.036546	-1.207437	-0.096778
N	0.076650	-2.421396	-0.774515
C	0.000000	0.000000	-0.836590
B	0.000000	0.000000	-2.353006
C	-0.036546	1.207437	-0.096778
N	-0.076650	2.421396	-0.774515
C	-0.028757	1.204747	1.317601
H	-0.047226	2.145619	1.866609
C	0.000000	0.000000	2.018564
C	-1.255219	4.182645	0.449513
H	-2.185712	3.685329	0.175836
C	-1.246393	5.348048	1.216187
C	0.000000	5.924448	1.519270
H	0.014446	6.835742	2.117690
C	1.213543	5.383451	1.089202
C	1.165986	4.210032	0.320324
H	2.078532	3.737313	-0.048636
C	-0.052842	3.624264	0.010005
C	-0.308007	3.842577	-2.711616
H	-0.276326	4.713925	-2.058820
C	-0.476265	4.026578	-4.084146
H	-0.564822	5.037338	-4.488271
C	-0.545599	2.912020	-4.918519
H	-0.704189	3.037951	-5.993022
C	-0.410181	1.632868	-4.384341
H	-0.493588	0.783746	-5.061921
C	-0.196113	1.386992	-3.007854
C	-0.190952	2.550133	-2.167376
C	0.308007	-3.842577	-2.711616
H	0.276326	-4.713925	-2.058820
C	0.476265	-4.026578	-4.084146
H	0.564822	-5.037338	-4.488271
C	0.545599	-2.912020	-4.918519
H	0.704189	-3.037951	-5.993022
C	0.410181	-1.632868	-4.384341
H	0.493588	-0.783746	-5.061921
C	0.196113	-1.386992	-3.007854
C	0.190952	-2.550133	-2.167376
C	-1.165986	-4.210032	0.320324
H	-2.078532	-3.737313	-0.048636
C	-1.213543	-5.383451	1.089202
C	0.000000	-5.924448	1.519270
H	-0.014446	-6.835742	2.117690
C	1.246393	-5.348048	1.216187
C	1.255219	-4.182645	0.449513
H	2.185712	-3.685329	0.175836
C	0.052842	-3.624264	0.010005
C	-2.532437	6.006771	1.727807
C	-2.628141	7.434283	1.165949
C	-2.499222	6.060101	3.263540
C	-3.782289	5.235032	1.298856
H	-3.547581	7.924280	1.524129
H	-2.654247	7.423997	0.065271
H	-1.776122	8.057080	1.477178
H	-2.433313	5.047960	3.692191
H	-3.416285	6.533837	3.648673
H	-1.642556	6.640886	3.637076
H	-3.877886	5.183846	0.203432
H	-4.680343	5.740294	1.685702
H	-3.785907	4.207136	1.692947
C	2.570843	6.013763	1.420137
C	3.288754	6.380083	0.111279
C	3.419557	5.001076	2.205469
C	2.429400	7.281997	2.264490
H	4.270866	6.829948	0.327666
H	2.701428	7.107194	-0.470709
H	3.458983	5.499144	-0.525402
H	2.927457	4.722360	3.150321
H	4.402939	5.434366	2.448192
H	3.594990	4.078593	1.632089
H	1.851661	8.060780	1.743257
H	3.426368	7.697346	2.476568
H	1.943339	7.080413	3.231547
C	-2.570843	-6.013763	1.420137
C	-3.419557	-5.001076	2.205469
C	-2.429400	-7.281997	2.264490
C	-3.288754	-6.380083	0.111279
H	-4.402939	-5.434366	2.448192
H	-3.594990	-4.078593	1.632089
H	-2.927457	-4.722360	3.150321

H	-1.851661	-8.060780	1.743257
H	-3.426368	-7.697346	2.476568
H	-1.943339	-7.080413	3.231547
H	-3.458983	-5.499144	-0.525402
H	-4.270866	-6.829948	0.327666
H	-2.701428	-7.107194	-0.470709
C	2.532437	-6.006771	1.727807
C	2.499222	-6.060101	3.263540
C	3.782289	-5.235032	1.298856
C	2.628141	-7.434283	1.165949
H	3.416285	-6.533837	3.648673
H	1.642556	-6.640886	3.637076
H	2.433313	-5.047960	3.692191
H	3.877886	-5.183846	0.203432
H	4.680343	-5.740294	1.685702
H	3.785907	-4.207136	1.692947
H	1.776122	-8.057080	1.477178
H	3.547581	-7.924280	1.524129
H	2.654247	-7.423997	0.065271

- BuDABNA **(3)**: T_2 (toluene) [electronic energy (au): -1915.5376]

H	0.000000	0.000000	3.115946
C	0.033172	-1.215875	1.345264
H	0.059215	-2.148908	1.905793
C	0.039922	-1.217500	-0.057766
N	0.083545	-2.424163	-0.770804
C	0.000000	0.000000	-0.789202
B	0.000000	0.000000	-2.328455
C	-0.039922	1.217500	-0.057766
N	-0.083545	2.424163	-0.770804
C	-0.033172	1.215875	1.345264
H	-0.059215	2.148908	1.905793
C	0.000000	0.000000	2.023342
C	-1.258425	4.189745	0.448924
H	-2.189644	3.689803	0.182783
C	-1.247284	5.357931	1.211405
C	0.000000	5.935296	1.508950
H	0.016330	6.848615	2.104269
C	1.212031	5.392577	1.076967
C	1.161926	4.216695	0.311790
H	2.073449	3.741071	-0.055895
C	-0.057801	3.631220	0.004407
C	-0.352227	3.824982	-2.714738
H	-0.292500	4.703986	-2.074496
C	-0.559069	3.997509	-4.099518
H	-0.654690	5.007006	-4.504981
C	-0.649901	2.887933	-4.914436
H	-0.836500	2.996928	-5.985845
C	-0.498218	1.599028	-4.357175
H	-0.616602	0.738471	-5.016290
C	-0.241041	1.374464	-2.983943
C	-0.223537	2.531906	-2.153218
C	0.352227	-3.824982	-2.714738
H	0.292500	-4.703986	-2.074496
C	0.559069	-3.997509	-4.099518
H	0.654690	-5.007006	-4.504981
C	0.649901	-2.887933	-4.914436
H	0.836500	-2.996928	-5.985845
C	0.498218	-1.599028	-4.357175
H	0.616602	-0.738471	-5.016290
C	0.241041	-1.374464	-2.983943
C	0.223537	-2.531906	-2.153218
C	-1.161926	-4.216695	0.311790
H	-2.073449	-3.741071	-0.055895
C	-1.212031	-5.392577	1.076967
C	0.000000	-5.935296	1.508950
H	-0.016330	-6.848615	2.104269
C	1.247284	-5.357931	1.211405
C	1.258425	-4.189745	0.448924
H	2.189644	-3.689803	0.182783
C	0.057801	-3.631220	0.004407
C	-2.531783	6.018822	1.724216
C	-2.629145	7.444881	1.159091
C	-2.494252	6.076089	3.259754
C	-3.783016	5.246054	1.301144
H	-3.547229	7.936231	1.519000
H	-2.658967	7.431814	0.058485
H	-1.775861	8.068229	1.465753
H	-2.426502	5.065033	3.690535
H	-3.410371	6.550552	3.646357
H	-1.636595	6.658020	3.629193
H	-3.881743	5.191878	0.206100
H	-4.679929	5.752464	1.689231
H	-3.785246	4.219238	1.697930
C	2.570501	6.023490	1.401943
C	3.288349	6.380161	0.090414
C	3.418037	5.014507	2.193364
C	2.431894	7.297648	2.237815
H	4.271260	6.830035	0.303254
H	2.701829	7.104352	-0.496117
H	3.457161	5.494664	-0.540313
H	2.926191	4.743383	3.140479
H	4.402797	5.447172	2.431823
H	3.589846	4.087322	1.626573
H	1.853960	8.073453	1.712304
H	3.429670	7.713340	2.445550
H	1.947214	7.103078	3.206966
C	-2.570501	-6.023490	1.401943
C	-3.418037	-5.014507	2.193364
C	-2.431894	-7.297648	2.237815
C	-3.288349	-6.380161	0.090414
H	-4.402797	-5.447172	2.431823
H	-3.589846	-4.087322	1.626573
H	-2.926191	-4.743383	3.140479
H	-1.853960	-8.073453	1.712304
H	-3.429670	-7.713340	2.445550
H	-1.947214	-7.103078	3.206966
H	-3.457161	-5.494664	-0.540313
H	-4.271260	-6.830035	0.303254
H	-2.701829	-7.104352	-0.496117
C	2.531783	-6.018822	1.724216
C	2.494252	-6.076089	3.259754
C	3.783016	-5.246054	1.301144
C	2.629145	-7.444881	1.159091
H	3.410371	-6.550552	3.646357
H	1.636595	-6.658020	3.629193
H	2.426502	-5.065033	3.690535
H	3.881743	-5.191878	0.206100
H	4.679929	-5.752464	1.689231
H	3.785246	-4.219238	1.697930
H	1.775861	-8.068229	1.465753
H	3.547229	-7.936231	1.519000
H	2.658967	-7.431814	0.058485

- 2CB-BuDABNA **(1)**: S_0 (gas) [electronic energy (au): -2654.6802]

H	0.000000	0.000000	5.526623
C	0.179718	1.203245	3.763394
H	0.317437	2.124555	4.326373
C	0.164577	1.210079	2.359855
N	0.316906	2.410733	1.670419
C	0.000000	0.000000	1.633951
B	0.000000	0.000000	0.114475
C	-0.164577	-1.210079	2.359855
N	-0.316906	-2.410733	1.670419
C	-0.179718	-1.203245	3.763394
H	-0.317437	-2.124555	4.326373
C	0.000000	0.000000	4.433487
C	0.523271	-4.347886	2.908168
H	1.529778	-4.005143	2.668050
C	0.304721	-5.502723	3.659177
C	-1.026674	-5.870026	3.922993
H	-1.205750	-6.771740	4.509094
C	-2.123617	-5.134659	3.469580
C	-1.863606	-3.978994	2.716575
H	-2.676756	-3.359713	2.332253
C	-0.558425	-3.596908	2.441983
C	-0.225039	-3.826591	-0.280225
H	-0.429306	-4.696162	0.342770
C	0.000000	-4.009278	-1.627952
H	-0.044572	-5.020269	-2.034424
C	0.292233	-2.923770	-2.472802
C	0.283841	-1.651798	-1.906842
H	0.505893	-0.802936	-2.550241
C	0.031952	-1.406186	-0.541781
C	-0.173833	-2.537237	0.295945
C	0.225039	3.826591	-0.280225
H	0.429306	4.696162	0.342770
C	0.000000	4.009278	-1.627952
H	0.044572	5.020269	-2.034424
C	-0.292233	2.923770	-2.472802
C	-0.283841	1.651798	-1.906842
H	-0.505893	0.802936	-2.550241
C	-0.031952	1.406186	-0.541781
C	0.173833	2.537237	0.295945
C	1.863606	3.978994	2.716575
H	2.676756	3.359713	2.332253
C	2.123617	5.134659	3.469580
C	1.026674	5.870026	3.922993
H	1.205750	6.771740	4.509094
C	-0.304721	5.502723	3.659177
C	-0.523271	4.347886	2.908168
H	-1.529778	4.005143	2.668050
C	0.558425	3.596908	2.441983
C	1.451215	-6.367424	4.195152
C	1.327279	-7.787403	3.620130
C	1.369329	-6.424496	5.728898
C	2.821685	-5.808358	3.806540
H	2.143475	-8.424901	3.995770
H	1.384022	-7.774694	2.520675
H	0.376675	-8.264497	3.901646
H	1.456950	-5.418058	6.166707
H	2.185899	-7.043997	6.132804
H	0.419862	-6.859727	6.074769
H	2.953665	-5.766397	2.714432
H	3.614847	-6.456371	4.209474
H	2.983511	-4.797830	4.212191
C	-3.573821	-5.537452	3.758715
C	-4.306700	-5.774102	2.428506
C	-4.266994	-4.405126	4.533259
C	-3.664042	-6.817125	4.592836
H	-5.354234	-6.060084	2.614339
H	-3.830121	-6.582817	1.853143
H	-4.315456	-4.873438	1.796717
H	-3.761706	-4.216112	5.493082
H	-5.314247	-4.672721	4.746170
H	-4.273617	-3.462189	3.966477
H	-3.208009	-7.676915	4.078284
H	-4.720438	-7.066016	4.775634
H	-3.178001	-6.704112	5.574200
C	3.573821	5.537452	3.758715
C	4.266994	4.405126	4.533259
C	3.664042	6.817125	4.592836
C	4.306700	5.774102	2.428506
H	5.314247	4.672721	4.746170
H	4.273617	3.462189	3.966477
H	3.761706	4.216112	5.493082
H	3.208009	7.676915	4.078284
H	4.720438	7.066016	4.775634
H	3.178001	6.704112	5.574200
H	4.315456	4.873438	1.796717
H	5.354234	6.060084	2.614339
H	3.830121	6.582817	1.853143
C	-1.451215	6.367424	4.195152
C	-1.369329	6.424496	5.728898
C	-2.821685	5.808358	3.806540
C	-1.327279	7.787403	3.620130
H	-2.185899	7.043997	6.132804
H	-0.419862	6.859727	6.074769
H	-1.456950	5.418058	6.166707
H	-2.953665	5.766397	2.714432
H	-3.614847	6.456371	4.209474
H	-2.983511	4.797830	4.212191
H	-0.376675	8.264497	3.901646
H	-2.143475	8.424901	3.995770
H	-1.384022	7.774694	2.520675
B	2.316274	-4.298024	-5.768876
C	2.132836	-3.157349	-4.516790
B	-0.375356	-4.311675	-4.783222
C	0.550589	-3.159882	-3.932618
C	3.252791	-2.876723	-3.543576
B	-0.413314	-3.750687	-6.466663
H	-1.369903	-3.968833	-7.158821
B	1.311510	-4.641810	-4.359206
B	0.717965	-5.039211	-5.975084
B	1.087195	-1.866408	-4.917795
B	-0.515779	-2.580463	-5.132263
B	2.180072	-2.572921	-6.112283
B	1.256010	-3.742209	-7.076850
B	0.494365	-2.218793	-6.545757
H	1.540192	-3.950149	-8.224693
H	-1.469422	-1.963681	-4.751645
H	1.308481	-0.805073	-4.419124
H	3.144368	-1.932582	-6.423228
H	0.221787	-1.316689	-7.286837
H	-1.239942	-4.875645	-4.175454
H	1.664186	-5.366423	-3.476045
H	3.376680	-4.853085	-5.838819
H	0.601899	-6.189152	-6.299859
H	4.005029	-3.673561	-3.602402
H	2.870962	-2.831868	-2.514156
H	3.734270	-1.919015	-3.778789
B	-2.316274	4.298024	-5.768876
C	-2.132836	3.157349	-4.516790

B	0.375356	4.311675	-4.783222
C	-0.550589	3.159882	-3.932618
C	-3.252791	2.876723	-3.543576
B	0.413314	3.750687	-6.466663
H	1.369903	3.968833	-7.158821
B	-1.311510	4.641810	-4.359206
B	-0.717965	5.039211	-5.975084
B	-1.087195	1.866408	-4.917795
B	0.515779	2.580463	-5.132263
B	-2.180072	2.572921	-6.112283
B	-1.256010	3.742209	-7.076850
B	-0.494365	2.218793	-6.545757
H	-1.540192	3.950149	-8.224693
H	1.469422	1.963681	-4.751645
H	-1.308481	0.805073	-4.419124
H	-3.144368	1.932582	-6.423228
H	-0.221787	1.316689	-7.286837
H	1.239942	4.875645	-4.175454
H	-1.664186	5.366423	-3.476045
H	-3.376680	4.853085	-5.838819
H	-0.601899	6.189152	-6.299859
H	-4.005029	3.673561	-3.602402
H	-2.870962	2.831868	-2.514156
H	-3.734270	1.919015	-3.778789

- 2CB-BuDABNA **(1)**: S_1-LE (gas) [electronic energy (au): -2654.5666]

H	0.000000	0.000000	5.567933
C	0.117033	1.198505	3.787256
H	0.211764	2.134616	4.335030
C	0.105611	1.198980	2.356854
N	0.197729	2.401186	1.692702
C	0.000000	0.000000	1.632989
B	0.000000	0.000000	0.088326
C	-0.105611	-1.198980	2.356854
N	-0.197729	-2.401186	1.692702
C	-0.117033	-1.198505	3.787256
H	-0.211764	-2.134616	4.335030
C	0.000000	0.000000	4.476414
C	0.777115	-4.288195	2.908995
H	1.757320	-3.899076	2.633490
C	0.638315	-5.447501	3.672548
C	-0.664820	-5.880809	3.974926
H	-0.780926	-6.786810	4.570086
C	-1.810815	-5.207594	3.546912
C	-1.632508	-4.044525	2.781276
H	-2.486757	-3.474348	2.410471
C	-0.355755	-3.596828	2.474327
C	-0.138338	-3.839329	-0.242304
H	-0.273715	-4.702328	0.407119
C	0.000000	-4.050357	-1.614098
H	-0.047950	-5.067129	-2.002031
C	0.191900	-2.966646	-2.475632
C	0.200095	-1.675087	-1.935104
H	0.359503	-0.840683	-2.611652
C	0.032570	-1.400899	-0.553639
C	-0.101148	-2.545871	0.293218
C	0.138338	3.839329	-0.242304
H	0.273715	4.702328	0.407119
C	0.000000	4.050357	-1.614098
H	0.047950	5.067129	-2.002031
C	-0.191900	2.966646	-2.475632
C	-0.200095	1.675087	-1.935104
H	-0.359503	0.840683	-2.611652
C	-0.032570	1.400899	-0.553639
C	0.101148	2.545871	0.293218
C	1.632508	4.044525	2.781276
H	2.486757	3.474348	2.410471
C	1.810815	5.207594	3.546912
C	0.664820	5.880809	3.974926
H	0.780926	6.786810	4.570086
C	-0.638315	5.447501	3.672548
C	-0.777115	4.288195	2.908995
H	-1.757320	3.899076	2.633490
C	0.355755	3.596828	2.474327
C	1.842313	-6.249664	4.178532
C	1.775346	-7.675085	3.607138
C	1.805171	-6.308314	5.713911
C	3.170789	-5.621442	3.751717
H	2.633818	-8.268594	3.959675
H	1.800174	-7.661175	2.506618
H	0.859185	-8.200265	3.915866
H	1.853797	-5.298227	6.149592
H	2.663181	-6.884578	6.095025
H	0.889426	-6.791344	6.086254
H	3.271084	-5.575906	2.656468
H	4.006966	-6.226465	4.133732
H	3.291749	-4.602931	4.151876
C	-3.229835	-5.685817	3.872779
C	-3.977779	-5.971337	2.560549
C	-3.966157	-4.587264	4.656078
C	-3.230411	-6.962297	4.716503
H	-5.003668	-6.312407	2.771888
H	-3.470790	-6.757061	1.979554
H	-4.049625	-5.077584	1.923016
H	-3.450622	-4.363460	5.603020
H	-4.991648	-4.910690	4.895244
H	-4.039587	-3.650965	4.082876
H	-2.740513	-7.799638	4.196218
H	-4.267077	-7.267096	4.925257
H	-2.728822	-6.816157	5.685650
C	3.229835	5.685817	3.872779
C	3.966157	4.587264	4.656078
C	3.230411	6.962297	4.716503
C	3.977779	5.971337	2.560549
H	4.991648	4.910690	4.895244
H	4.039587	3.650965	4.082876
H	3.450622	4.363460	5.603020
H	2.740513	7.799638	4.196218
H	4.267077	7.267096	4.925257
H	2.728822	6.816157	5.685650
H	4.049625	5.077584	1.923016
H	5.003668	6.312407	2.771888
H	3.470790	6.757061	1.979554
C	-1.842313	6.249664	4.178532
C	-1.805171	6.308314	5.713911
C	-3.170789	5.621442	3.751717
C	-1.775346	7.675085	3.607138
H	-2.663181	6.884578	6.095025
H	-0.889426	6.791344	6.086254
H	-1.853797	5.298227	6.149592
H	-3.271084	5.575906	2.656468
H	-4.006966	6.226465	4.133732
H	-3.291749	4.602931	4.151876
H	-0.859185	8.200265	3.915866
H	-2.633818	8.268594	3.959675
H	-1.800174	7.661175	2.506618
B	1.948585	-4.326404	-5.930297
C	1.856383	-3.184774	-4.668002
B	-0.648932	-4.374127	-4.719216
C	0.331599	-3.208748	-3.952444
C	3.048441	-2.887762	-3.789774
B	-0.836299	-3.816323	-6.394188
H	-1.845486	-4.047767	-7.002851
B	1.071863	-4.681257	-4.440517
B	0.349072	-5.088473	-6.000676
B	0.766134	-1.908576	-4.982375
B	-0.839726	-2.643894	-5.056869
B	1.762653	-2.604201	-6.263228
B	0.774702	-3.787006	-7.144916
B	0.041212	-2.273168	-6.552546
H	0.963043	-3.994317	-8.312686
H	-1.765811	-2.037147	-4.599537
H	1.019285	-0.844719	-4.506935
H	2.688921	-1.952860	-6.656279
H	-0.306588	-1.376486	-7.268806
H	-1.452328	-4.948034	-4.041135
H	1.512124	-5.402348	-3.595254
H	3.006312	-4.868546	-6.089117
H	0.221815	-6.240692	-6.314061
H	3.812846	-3.663355	-3.926085
H	2.753513	-2.868024	-2.731321
H	3.482623	-1.913734	-4.048746
B	-1.948585	4.326404	-5.930297
C	-1.856383	3.184774	-4.668002
B	0.648932	4.374127	-4.719216
C	-0.331599	3.208748	-3.952444
C	-3.048441	2.887762	-3.789774
B	0.836299	3.816323	-6.394188
H	1.845486	4.047767	-7.002851
B	-1.071863	4.681257	-4.440517
B	-0.349072	5.088473	-6.000676
B	-0.766134	1.908576	-4.982375
B	0.839726	2.643894	-5.056869
B	-1.762653	2.604201	-6.263228
B	-0.774702	3.787006	-7.144916
B	-0.041212	2.273168	-6.552546
H	-0.963043	3.994317	-8.312686
H	1.765811	2.037147	-4.599537
H	-1.019285	0.844719	-4.506935
H	-2.688921	1.952860	-6.656279
H	0.306588	1.376486	-7.268806
H	1.452328	4.948034	-4.041135
H	-1.512124	5.402348	-3.595254
H	-3.006312	4.868546	-6.089117
H	-0.221815	6.240692	-6.314061
H	-3.812846	3.663355	-3.926085
H	-2.753513	2.868024	-2.731321
H	-3.482623	1.913734	-4.048746

- 2CB-BuDABNA **(1)**: S_1-CT (gas) [electronic energy (au): -2654.5750]

B	-2.206172	5.935201	2.597756
C	-2.631197	4.290086	2.596009
B	-3.076961	5.126140	-0.014135
C	-3.129957	3.812451	1.079356
C	-1.891295	3.299110	3.465474
B	-4.247728	6.308184	0.613254
H	-4.849821	7.022307	-0.140361
B	-1.823208	4.909397	1.212623
B	-2.507813	6.511689	0.942471
B	-4.297218	4.014360	2.330626
B	-4.608403	4.576132	0.681908
B	-3.741524	5.389704	3.283720
B	-3.706555	6.814015	2.228919
B	-5.010961	5.606612	2.065740
H	-3.900327	7.910327	2.676945
H	-5.388197	3.971034	0.004035
H	-4.766130	3.021015	2.805879
H	-3.885309	5.333248	4.472468
H	-6.152904	5.801525	2.380068
H	-2.810446	4.866288	-1.151168
H	-0.716079	4.521920	1.002254
H	-1.301807	6.258679	3.313286
H	-1.828034	7.367403	0.451202
H	-0.118567	4.283180	-1.801499
H	-2.063105	3.557299	4.518197
H	-0.811494	3.332490	3.272057
H	0.075349	-5.532720	-0.289153
C	-1.141677	-3.770650	-0.335986
H	-2.064901	-4.327659	-0.485866
C	-1.165398	-2.354286	-0.235051
N	-2.371685	-1.691113	-0.276438
C	0.037810	-1.628396	-0.091690
B	0.033185	-0.106629	-0.074800
C	1.251785	-2.349885	0.016452
N	2.441345	-1.667472	0.174493
C	1.261416	-3.761759	-0.033408
H	2.194734	-4.313989	0.059700
C	0.064088	-4.442688	-0.225974
C	3.985838	-2.790460	1.699581
H	3.345177	-2.459104	2.516707
C	5.140930	-3.538064	1.933527
C	5.912452	-3.915716	0.820948
H	6.817540	-4.497145	0.996649
C	5.579227	-3.577540	-0.492780
C	4.411721	-2.825149	-0.692097
H	4.099711	-2.520817	-1.693324
C	3.637918	-2.444695	0.394159
C	3.860830	0.278951	0.160527
H	4.718030	-0.342225	0.415239
C	4.046722	1.610914	-0.116367
H	5.049034	2.037479	-0.064395
C	2.978861	2.440806	-0.531152
C	1.690088	1.887520	-0.503101
H	0.857097	2.533988	-0.780745
C	1.432629	0.548565	-0.170372
C	2.566514	-0.291386	0.068451
C	-3.799918	0.215448	0.092516
H	-4.675930	-0.417543	-0.039033
C	-3.972899	1.536331	0.439099
H	-4.985963	1.916544	0.570172
C	-2.874582	2.394192	0.641812
C	-1.601633	1.875615	0.432451
H	-0.739076	2.525785	0.572303
C	-1.368405	0.526163	0.083326
C	-2.502549	-0.322949	-0.044912
C	-4.233916	-3.039915	0.560420
H	-3.835080	-2.896245	1.566985
C	-5.400006	-3.789893	0.343033
C	-5.846759	-3.920494	-0.974021
H	-6.751568	-4.497767	-1.164476
C	-5.187494	-3.340756	-2.071802
C	-4.030551	-2.602300	-1.818390
H	-3.472235	-2.123520	-2.622872

C	-3.567412	-2.460391	-0.510176
C	5.587548	-3.942500	3.342406
C	6.972202	-3.337367	3.624384
C	5.666831	-5.475006	3.428862
C	4.616365	-3.447566	4.416427
H	7.311266	-3.616672	4.634333
H	6.943555	-2.238418	3.567356
H	7.730398	-3.691430	2.910156
H	4.685992	-5.934793	3.231323
H	5.989671	-5.782939	4.435795
H	6.385911	-5.892842	2.708638
H	4.538668	-2.349522	4.427339
H	4.972165	-3.759995	5.409637
H	3.606461	-3.865629	4.283050
C	6.431585	-3.980421	-1.700656
C	6.917212	-2.709939	-2.417130
C	5.578283	-4.821368	-2.663609
C	7.654689	-4.804917	-1.293989
H	7.530729	-2.976744	-3.291836
H	7.532169	-2.088152	-1.748623
H	6.081835	-2.090503	-2.776238
H	5.215940	-5.739061	-2.174412
H	6.174818	-5.117253	-3.540732
H	4.703145	-4.266170	-3.033384
H	8.326787	-4.243575	-0.627007
H	8.233260	-5.072850	-2.190694
H	7.371976	-5.743379	-0.792296
C	-6.126726	-4.419661	1.536347
C	-5.171123	-5.382312	2.259318
C	-7.368623	-5.204171	1.107920
C	-6.564988	-3.307133	2.502379
H	-5.675332	-5.842981	3.123344
H	-4.273786	-4.868699	2.636050
H	-4.841396	-6.190795	1.588402
H	-8.110055	-4.561691	0.608734
H	-7.855186	-5.638404	1.994197
H	-7.118212	-6.034961	0.430382
H	-5.709185	-2.730800	2.884880
H	-7.087844	-3.739121	3.370152
H	-7.251839	-2.602397	2.008781
C	-5.749594	-3.530641	-3.484540
C	-5.796688	-5.031416	-3.813149
C	-4.892875	-2.830915	-4.541901
C	-7.169462	-2.944728	-3.544859
H	-6.200784	-5.186531	-4.825833
H	-6.436587	-5.588190	-3.112355
H	-4.790281	-5.476626	-3.777574
H	-4.842537	-1.743439	-4.378684
H	-5.329862	-2.992093	-5.538720
H	-3.865209	-3.225163	-4.566890
H	-7.850846	-3.439340	-2.836595
H	-7.590825	-3.071613	-4.554434
H	-7.164224	-1.868730	-3.312248
B	2.351730	6.825627	-1.282902
C	1.689918	5.480628	-1.893682
B	4.276616	4.788552	-0.161153
C	3.253370	3.818462	-0.969310
C	0.223422	5.278158	-2.129370
B	5.088969	5.829619	-1.346554
H	6.255980	6.098463	-1.212668
B	2.429223	5.190398	-0.387049
B	3.777666	6.472172	-0.299289
B	2.853116	4.399573	-2.513897
B	4.599465	4.189792	-1.812087
B	2.677643	6.218321	-2.935183
B	3.968188	7.012556	-2.004071
B	4.300221	5.510096	-2.927734
H	4.355638	8.105292	-2.331545
H	5.326717	3.278522	-2.116665
H	2.465077	3.578131	-3.309390
H	2.191835	6.605572	-3.966904
H	4.904637	5.503006	-3.969475
H	4.742324	4.364167	0.866662
H	1.703019	5.006667	0.560324
H	1.597496	7.708956	-0.966260
H	3.993773	7.189750	0.643383
H	-0.017607	5.366358	-3.199555
H	-0.370026	6.026355	-1.583491
H	-2.251497	2.275247	3.298882

- 2CB-BuDABNA **(1)**: S_0 (THF) [electronic energy (au): -2654.6936]

H	0.000000	0.000000	5.515752
C	-0.143467	-1.209722	3.753029
H	-0.252117	-2.134055	4.317702
C	-0.127882	-1.216968	2.349444
N	-0.243943	-2.420603	1.656406
C	0.000000	0.000000	1.627042
B	0.000000	0.000000	0.110491
C	0.127882	1.216968	2.349444
N	0.243943	2.420603	1.656406
C	0.143467	1.209722	3.753029
H	0.252117	2.134055	4.317702
C	0.000000	0.000000	4.422653
C	-0.634737	4.342106	2.892991
H	-1.635178	3.976726	2.660403
C	-0.437914	5.504454	3.639161
C	0.886549	5.902842	3.894754
H	1.048145	6.809970	4.477712
C	1.997858	5.191285	3.437106
C	1.760890	4.027243	2.688559
H	2.586955	3.426694	2.301776
C	0.462488	3.615807	2.423846
C	0.085415	3.826157	-0.301473
H	0.287917	4.705445	0.308444
C	-0.185408	3.993855	-1.643823
H	-0.174437	5.003778	-2.054524
C	-0.487309	2.895361	-2.469496
C	-0.412909	1.626083	-1.900738
H	-0.624436	0.763066	-2.530455
C	-0.104345	1.399350	-0.545792
C	0.078978	2.539744	0.285343
C	-0.085415	-3.826157	-0.301473
H	-0.287917	-4.705445	0.308444
C	0.185408	-3.993855	-1.643823
H	0.174437	-5.003778	-2.054524
C	0.487309	-2.895361	-2.469496
C	0.412909	-1.626083	-1.900738
H	0.624436	-0.763066	-2.530455
C	0.104345	-1.399350	-0.545792
C	-0.078978	-2.539744	0.285343
C	-1.760890	-4.027243	2.688559
H	-2.586955	-3.426694	2.301776
C	-1.997858	-5.191285	3.437106
C	-0.886549	-5.902842	3.894754
H	-1.048145	-6.809970	4.477712
C	0.437914	-5.504454	3.639161
C	0.634737	-4.342106	2.892991
H	1.635178	-3.976726	2.660403
C	-0.462488	-3.615807	2.423846
C	-1.600821	6.345477	4.177136
C	-1.513653	7.763286	3.589714
C	-1.508073	6.418296	5.709658
C	-2.960287	5.750573	3.803607
H	-2.341439	8.383755	3.968428
H	-1.580649	7.740018	2.490798
H	-0.571581	8.263162	3.860386
H	-1.568759	5.413844	6.156874
H	-2.337216	7.021370	6.112642
H	-0.567180	6.880945	6.043266
H	-3.099896	5.697398	2.712869
H	-3.764534	6.383053	4.209165
H	-3.093782	4.739321	4.217782
C	3.439741	5.628608	3.717177
C	4.159571	5.877700	2.381979
C	4.163454	4.514405	4.490285
C	3.504015	6.912488	4.547159
H	5.201006	6.187814	2.563133
H	3.661831	6.674932	1.808150
H	4.185612	4.974855	1.753545
H	3.668167	4.316843	5.453754
H	5.204815	4.808796	4.696376
H	4.189985	3.571135	3.924398
H	3.024477	7.759436	4.032693
H	4.555676	7.185431	4.722785
H	3.025442	6.790794	5.531095
C	-3.439741	-5.628608	3.717177
C	-4.163454	-4.514405	4.490285
C	-3.504015	-6.912488	4.547159
C	-4.159571	-5.877700	2.381979
H	-5.204815	-4.808796	4.696376
H	-4.189985	-3.571135	3.924398
H	-3.668167	-4.316843	5.453754
H	-3.024477	-7.759436	4.032693
H	-4.555676	-7.185431	4.722785
H	-3.025442	-6.790794	5.531095
H	-4.185612	-4.974855	1.753545
H	-5.201006	-6.187814	2.563133
H	-3.661831	-6.674932	1.808150
C	1.600821	-6.345477	4.177136
C	1.508073	-6.418296	5.709658
C	2.960287	-5.750573	3.803607
C	1.513653	-7.763286	3.589714
H	2.337216	-7.021370	6.112642
H	0.567180	-6.880945	6.043266
H	1.568759	-5.413844	6.156874
H	3.099896	-5.697398	2.712869
H	3.764534	-6.383053	4.209165
H	3.093782	-4.739321	4.217782
H	0.571581	-8.263162	3.860386
H	2.341439	-8.383755	3.968428
H	1.580649	-7.740018	2.490798
B	-2.496456	4.381412	-5.734855
C	-2.427944	3.347018	-4.386989
B	0.227987	3.942677	-4.950377
C	-0.821706	3.084162	-3.919438
C	-3.485150	3.380736	-3.309916
B	0.000000	3.233327	-6.563808
H	0.912786	3.187245	-7.344615
B	-1.318172	4.640636	-4.445832
B	-0.808931	4.761668	-6.133839
B	-1.694941	1.842993	-4.719159
B	-0.007511	2.194781	-5.123298
B	-2.729973	2.642461	-5.906035
B	-1.686776	3.507940	-7.049563
B	-1.192031	1.916745	-6.412952
H	-2.026076	3.666173	-8.191860
H	0.840408	1.432758	-4.757753
H	-2.067960	0.903097	-4.085754
H	-3.823597	2.185446	-6.083339
H	-1.165927	0.914250	-7.073138
H	1.234355	4.374561	-4.464286
H	-1.448357	5.511803	-3.640682
H	-3.426682	5.135050	-5.793478
H	-0.503745	5.830007	-6.590846
H	-3.957927	4.369952	-3.269951
H	-3.042275	3.165514	-2.327672
H	-4.255879	2.627363	-3.514957
B	2.496456	-4.381412	-5.734855
C	2.427944	-3.347018	-4.386989
B	-0.227987	-3.942677	-4.950377
C	0.821706	-3.084162	-3.919438
C	3.485150	-3.380736	-3.309916
B	0.000000	-3.233327	-6.563808
H	-0.912786	-3.187245	-7.344615
B	1.318172	-4.640636	-4.445832
B	0.808931	-4.761668	-6.133839
B	1.694941	-1.842993	-4.719159
B	0.007511	-2.194781	-5.123298
B	2.729973	-2.642461	-5.906035
B	1.686776	-3.507940	-7.049563
B	1.192031	-1.916745	-6.412952
H	2.026076	-3.666173	-8.191860
H	-0.840408	-1.432758	-4.757753
H	2.067960	-0.903097	-4.085754
H	3.823597	-2.185446	-6.083339
H	1.165927	-0.914250	-7.073138
H	-1.234355	-4.374561	-4.464286
H	1.448357	-5.511803	-3.640682
H	3.426682	-5.135050	-5.793478
H	0.503745	-5.830007	-6.590846
H	3.957927	-4.369952	-3.269951
H	3.042275	-3.165514	-2.327672
H	4.255879	-2.627363	-3.514957

- 2CB-BuDABNA **(1)**: S_1-LE (THF) [electronic energy (au): -2654.5825]

H	0.000000	0.000000	5.561079
C	-0.109372	-1.199586	3.778878
H	-0.197277	-2.134951	4.328962
C	-0.100578	-1.200304	2.353943
N	-0.191680	-2.405541	1.685465
C	0.000000	0.000000	1.625792
B	0.000000	0.000000	0.090550
C	0.100578	1.200304	2.353943
N	0.191680	2.405541	1.685465
C	0.109372	1.199586	3.778878
H	0.197277	2.134951	4.328962
C	0.000000	0.000000	4.470005
C	-0.793240	4.290348	2.897627
H	-1.772074	3.900202	2.618517
C	-0.659474	5.449325	3.662957
C	0.641558	5.883069	3.975000
H	0.753068	6.788308	4.572265
C	1.791069	5.209335	3.556267

C	1.619735	4.046412	2.788367
H	2.477815	3.475215	2.428099
C	0.344393	3.602056	2.470963
C	0.140617	3.842984	-0.246210
H	0.277110	4.706268	0.402452
C	0.000000	4.052069	-1.616593
H	0.045820	5.070553	-2.001246
C	-0.194062	2.968572	-2.478088
C	-0.200404	1.674838	-1.936561
H	-0.360846	0.839744	-2.612144
C	-0.032633	1.402167	-0.556156
C	0.100319	2.547481	0.294484
C	-0.140617	-3.842984	-0.246210
H	-0.277110	-4.706268	0.402452
C	0.000000	-4.052069	-1.616593
H	-0.045820	-5.070553	-2.001246
C	0.194062	-2.968572	-2.478088
C	0.200404	-1.674838	-1.936561
H	0.360846	-0.839744	-2.612144
C	0.032633	-1.402167	-0.556156
C	-0.100319	-2.547481	0.294484
C	-1.619735	-4.046412	2.788367
H	-2.477815	-3.475215	2.428099
C	-1.791069	-5.209335	3.556267
C	-0.641558	-5.883069	3.975000
H	-0.753068	-6.788308	4.572265
C	0.659474	-5.449325	3.662957
C	0.793240	-4.290348	2.897627
H	1.772074	-3.900202	2.618517
C	-0.344393	-3.602056	2.470963
C	-1.867373	6.248405	4.163910
C	-1.796306	7.676936	3.600734
C	-1.839618	6.299484	5.699879
C	-3.192224	5.621227	3.724797
H	-2.657289	8.267037	3.952774
H	-1.816300	7.668642	2.499836
H	-0.881818	8.199611	3.918644
H	-1.891611	5.286945	6.129651
H	-2.700937	6.873779	6.076368
H	-0.926405	6.781880	6.079305
H	-3.284177	5.580583	2.628515
H	-4.030349	6.224971	4.104513
H	-3.314636	4.600730	4.119347
C	3.207071	5.685781	3.897205
C	3.969317	5.969765	2.592837
C	3.933507	4.585455	4.687481
C	3.199521	6.962258	4.740865
H	4.993177	6.308664	2.817035
H	3.470101	6.757686	2.007674
H	4.046444	5.074751	1.957426
H	3.407019	4.362001	5.628524
H	4.955981	4.909791	4.938003
H	4.013133	3.650053	4.113444
H	2.715446	7.799790	4.215442
H	4.234748	7.264446	4.960270
H	2.686597	6.816219	5.703985
C	-3.207071	-5.685781	3.897205
C	-3.933507	-4.585455	4.687481
C	-3.199521	-6.962258	4.740865
C	-3.969317	-5.969765	2.592837
H	-4.955981	-4.909791	4.938003
H	-4.013133	-3.650053	4.113444
H	-3.407019	-4.362001	5.628524
H	-2.715446	-7.799790	4.215442
H	-4.234748	-7.264446	4.960270
H	-2.686597	-6.816219	5.703985
H	-4.046444	-5.074751	1.957426
H	-4.993177	-6.308664	2.817035
H	-3.470101	-6.757686	2.007674
C	1.867373	-6.248405	4.163910
C	1.839618	-6.299484	5.699879
C	3.192224	-5.621227	3.724797
C	1.796306	-7.676936	3.600734
H	2.700937	-6.873779	6.076368
H	0.926405	-6.781880	6.079305
H	1.891611	-5.286945	6.129651
H	3.284177	-5.580583	2.628515
H	4.030349	-6.224971	4.104513
H	3.314636	-4.600730	4.119347
H	0.881818	-8.199611	3.918644
H	2.657289	-8.267037	3.952774
H	1.816300	-7.668642	2.499836
B	-1.927530	4.374238	-5.928410
C	-1.871673	3.236995	-4.661026
B	0.678880	4.333260	-4.735095
C	-0.336157	3.209192	-3.953123
C	-3.066671	2.990594	-3.771796
B	0.833071	3.759968	-6.408122
H	1.845710	3.950631	-7.027703
B	-1.028673	4.703254	-4.445101
B	-0.301614	5.075652	-6.013339
B	-0.827918	1.921722	-4.972282
B	0.803748	2.596194	-5.064478
B	-1.807562	2.644893	-6.251741
B	-0.782689	3.785100	-7.146935
B	-0.101182	2.250285	-6.550184
H	-0.969621	3.992317	-8.316392
H	1.712233	1.957297	-4.615566
H	-1.113498	0.868435	-4.493099
H	-2.756713	2.024951	-6.640738
H	0.209772	1.338792	-7.266743
H	1.509151	4.883581	-4.070256
H	-1.434122	5.448599	-3.605011
H	-2.963278	4.956027	-6.088238
H	-0.133298	6.220483	-6.337282
H	-3.819246	3.771865	-3.936484
H	-2.770394	3.010197	-2.713928
H	-3.516008	2.013847	-3.990181
B	1.927530	-4.374238	-5.928410
C	1.871673	-3.236995	-4.661026
B	-0.678880	-4.333260	-4.735095
C	0.336157	-3.209192	-3.953123
C	3.066671	-2.990594	-3.771796
B	-0.833071	-3.759968	-6.408122
H	-1.845710	-3.950631	-7.027703
B	1.028673	-4.703254	-4.445101
B	0.301614	-5.075652	-6.013339
B	0.827918	-1.921722	-4.972282
B	-0.803748	-2.596194	-5.064478
B	1.807562	-2.644893	-6.251741
B	0.782689	-3.785100	-7.146935
B	0.101182	-2.250285	-6.550184
H	0.969621	-3.992317	-8.316392
H	-1.712233	-1.957297	-4.615566
H	1.113498	-0.868435	-4.493099
H	2.756713	-2.024951	-6.640738
H	-0.209772	-1.338792	-7.266743
H	-1.509151	-4.883581	-4.070256
H	1.434122	-5.448599	-3.605011
H	2.963278	-4.956027	-6.088238
H	0.133298	-6.220483	-6.337282
H	3.819246	-3.771865	-3.936484
H	2.770394	-3.010197	-2.713928
H	3.516008	-2.013847	-3.990181

- 2CB-BuDABNA (**1**): S_1-CT (THF) [electronic energy (au): -2654.5928]

B	2.237943	5.912538	-2.630368
C	2.689713	4.274949	-2.604731
B	3.015120	5.132324	0.018861
C	3.134775	3.812427	-1.060483
C	2.005996	3.262593	-3.494724
B	4.188244	6.331440	-0.565571
H	4.745186	7.063401	0.207720
B	1.818659	4.885581	-1.257854
B	2.461535	6.501531	-0.967525
B	4.346949	4.029117	-2.265930
B	4.582137	4.604468	-0.608456
B	3.808380	5.391256	-3.249388
B	3.705350	6.819443	-2.204470
B	5.021348	5.636032	-1.980472
H	3.898297	7.919106	-2.648840
H	5.344237	4.018113	0.103918
H	4.857259	3.047204	-2.717440
H	4.005185	5.338245	-4.430113
H	6.171722	5.853994	-2.248192
H	2.708732	4.879640	1.147403
H	0.708730	4.487121	-1.088516
H	1.358358	6.223174	-3.382057
H	1.747020	7.349874	-0.508349
H	0.106879	4.249997	1.776935
H	2.216707	3.512662	-4.542126
H	0.919112	3.273947	-3.346003
H	-0.084566	-5.521538	0.284542
C	1.133594	-3.760615	0.337207
H	2.055034	-4.321320	0.482816
C	1.159051	-2.344815	0.241881
N	2.367016	-1.684281	0.285392
C	-0.043997	-1.616930	0.100196
B	-0.035423	-0.099407	0.076336
C	-1.258153	-2.337726	-0.007286
N	-2.450624	-1.654599	-0.163366
C	-1.269319	-3.747773	0.038350
H	-2.201469	-4.301379	-0.055946
C	-0.072269	-4.431738	0.226483
C	-3.986675	-2.769537	-1.701486
H	-3.343012	-2.431470	-2.513523
C	-5.137714	-3.521092	-1.943416
C	-5.910929	-3.911128	-0.835931
H	-6.812103	-4.496531	-1.019011
C	-5.581623	-3.583237	0.481600
C	-4.418354	-2.826652	0.691440
H	-4.106197	-2.534729	1.696373
C	-3.646251	-2.433814	-0.391535
C	-3.869603	0.287118	-0.140411
H	-4.728144	-0.334260	-0.389647
C	-4.055465	1.618637	0.136996
H	-5.062185	2.036587	0.090934
C	-2.981815	2.448941	0.535335
C	-1.693089	1.897229	0.500250
H	-0.854423	2.541646	0.765715
C	-1.437585	0.557581	0.171051
C	-2.573566	-0.284664	-0.056585
C	3.800696	0.220102	-0.057881
H	4.675172	-0.412254	0.084985
C	3.978821	1.539977	-0.402172
H	4.995641	1.915403	-0.518161
C	2.880467	2.395299	-0.623038
C	1.604575	1.877235	-0.430051
H	0.743132	2.523625	-0.590277
C	1.366643	0.530918	-0.077402
C	2.500415	-0.320499	0.061832
C	4.227694	-3.025525	-0.565745
H	3.832809	-2.865583	-1.571380
C	5.388742	-3.785239	-0.353936
C	5.828431	-3.940497	0.963103
H	6.728554	-4.527003	1.148026
C	5.165853	-3.376416	2.067385
C	4.014333	-2.626616	1.821664
H	3.452391	-2.160989	2.631268
C	3.561538	-2.461382	0.512794
C	-5.575802	-3.921175	-3.356006
C	-6.965276	-3.327642	-3.638809
C	-5.641184	-5.454029	-3.450340
C	-4.605272	-3.411451	-4.423534
H	-7.296878	-3.606163	-4.651489
H	-6.945362	-2.228502	-3.577161
H	-7.721651	-3.693032	-2.928252
H	-4.655988	-5.904817	-3.253328
H	-5.958788	-5.757932	-4.460226
H	-6.358656	-5.881142	-2.733888
H	-4.535928	-2.312749	-4.425601
H	-4.956873	-3.720986	-5.419246
H	-3.592693	-3.822159	-4.288557
C	-6.431998	-4.006118	1.683961
C	-6.924430	-2.747247	2.416020
C	-5.573310	-4.852940	2.636982
C	-7.650193	-4.832525	1.266774
H	-7.535044	-3.029931	3.287950
H	-7.544352	-2.122631	1.754337
H	-6.090223	-2.129208	2.780601
H	-5.205871	-5.762042	2.135776
H	-6.169561	-5.162845	3.509586
H	-4.701769	-4.295990	3.012568
H	-8.324125	-4.267671	0.604586
H	-8.226545	-5.113729	2.160976
H	-7.360683	-5.762790	0.754140
C	6.115824	-4.402161	-1.553437
C	5.155303	-5.346452	-2.294155
C	7.350226	-5.201822	-1.131535
C	6.565192	-3.278159	-2.500997
H	5.660915	-5.796837	-3.162830
H	4.263330	-4.819803	-2.665652
H	4.818164	-6.162497	-1.636104
H	8.093485	-4.572087	-0.618936
H	7.836012	-5.625574	-2.023404
H	7.090232	-6.040187	-0.467136
H	5.714028	-2.690727	-2.876994
H	7.087415	-3.702759	-3.372862
H	7.256285	-2.587084	-1.993780
C	5.716859	-3.597314	3.479891
C	5.754567	-5.104745	3.778177
C	4.855663	-2.914916	4.544845
C	7.139403	-3.020603	3.561918
H	6.150314	-5.280538	4.790895
H	6.397704	-5.649158	3.070663
H	4.745754	-5.543117	3.726288
H	4.811817	-1.824218	4.402069

H	5.285722	-3.099853	5.540687
H	3.825894	-3.304331	4.552612
H	7.821789	-3.504757	2.847437
H	7.551945	-3.172148	4.571869
H	7.140157	-1.939598	3.352644
B	-2.326255	6.830704	1.275903
C	-1.683405	5.471289	1.880234
B	-4.265805	4.815207	0.164109
C	-3.252496	3.832946	0.969598
C	-0.218253	5.249100	2.106458
B	-5.068588	5.865156	1.348004
H	-6.235315	6.147016	1.218019
B	-2.427536	5.205401	0.370490
B	-3.758385	6.498151	0.297160
B	-2.857313	4.417378	2.522923
B	-4.590091	4.222954	1.815747
B	-2.653130	6.233395	2.925572
B	-3.934935	7.042141	2.000486
B	-4.282627	5.547185	2.928489
H	-4.317070	8.140418	2.324931
H	-5.331666	3.324943	2.121898
H	-2.480721	3.600245	3.327422
H	-2.154360	6.628679	3.948938
H	-4.892763	5.556610	3.969545
H	-4.745639	4.394833	-0.858123
H	-1.706649	5.023658	-0.579251
H	-1.560865	7.708625	0.967684
H	-3.978890	7.223035	-0.641893
H	0.033435	5.335721	3.174165
H	0.381370	5.985793	1.552480
H	2.380375	2.248649	-3.302301

- 2CB-BuDABNA **(1)**: S_1-CT (toluene) [electronic energy (au): -2654.5853]

B	2.234086	5.919843	-2.620493
C	2.672425	4.278276	-2.602562
B	3.047061	5.129458	0.015172
C	3.138040	3.810618	-1.068979
C	1.964209	3.274801	-3.483766
B	4.221834	6.318999	-0.587427
H	4.798174	7.043374	0.177631
B	1.827206	4.895733	-1.241725
B	2.489736	6.505424	-0.960816
B	4.333194	4.018031	-2.292719
B	4.599448	4.589209	-0.638964
B	3.790071	5.385180	-3.265602
B	3.716674	6.813272	-2.217951
B	5.026252	5.618018	-2.016880
H	3.911975	7.910592	-2.664981
H	5.367336	3.994481	0.060596
H	4.825162	3.029817	-2.752810
H	3.965754	5.328922	-4.449773
H	6.173963	5.824116	-2.303392
H	2.756085	4.875151	1.147495
H	0.717527	4.503372	-1.056030
H	1.345189	6.236021	-3.358476
H	1.790538	7.358572	-0.489942
H	0.116615	4.269508	1.767060
H	2.153535	3.531143	-4.533823
H	0.880590	3.292179	-3.312249
H	-0.084513	-5.525428	0.283671
C	1.134318	-3.764780	0.334971
H	2.056190	-4.324334	0.482932
C	1.160060	-2.348510	0.237573
N	2.367611	-1.688017	0.281685
C	-0.042415	-1.620635	0.093828
B	-0.034410	-0.101364	0.072555
C	-1.257007	-2.340905	-0.014621
N	-2.447507	-1.657710	-0.172257
C	-1.268403	-3.751951	0.033053
H	-2.201548	-4.304062	-0.061206
C	-0.071852	-4.435528	0.223671
C	-3.986642	-2.777882	-1.704255
H	-3.343420	-2.444919	-2.518793
C	-5.139807	-3.527335	-1.942376
C	-5.913660	-3.909067	-0.832626
H	-6.817052	-4.491982	-1.012347
C	-5.584070	-3.574066	0.482952
C	-4.418465	-2.819926	0.687994
H	-4.107790	-2.520474	1.691213
C	-3.644208	-2.435527	-0.396506
C	-3.865538	0.286847	-0.152499
H	-4.723857	-0.333959	-0.404117
C	-4.050688	1.618417	0.126231
H	-5.055591	2.040095	0.078865
C	-2.979053	2.447854	0.531339
C	-1.690674	1.895311	0.497576
H	-0.853850	2.539884	0.768337
C	-1.435043	0.555723	0.166485
C	-2.570549	-0.284810	-0.065754
C	3.800985	0.216322	-0.069290
H	4.675275	-0.416936	0.071415
C	3.978487	1.536404	-0.414609
H	4.994298	1.913191	-0.534469
C	2.881200	2.393505	-0.630694
C	1.605798	1.876308	-0.432954
H	0.744683	2.525074	-0.586383
C	1.368167	0.528861	-0.081706
C	2.501230	-0.322391	0.055143
C	4.230656	-3.031373	-0.561354
H	3.837246	-2.875623	-1.568284
C	5.392752	-3.788273	-0.345701
C	5.831169	-3.937003	0.972400
H	6.732241	-4.520813	1.160848
C	5.167100	-3.368936	2.073598
C	4.014808	-2.621981	1.823097
H	3.452710	-2.152399	2.630363
C	3.561819	-2.462983	0.513459
C	-5.581055	-3.931145	-3.352958
C	-6.967071	-3.330241	-3.637245
C	-5.655372	-5.463887	-3.441292
C	-4.608386	-3.431493	-4.423329
H	-7.301587	-3.609725	-4.648629
H	-6.941314	-2.231166	-3.579013
H	-7.725473	-3.688340	-2.925192
H	-4.672984	-5.920259	-3.243202
H	-5.975632	-5.770585	-4.449438
H	-6.374560	-5.884542	-2.722787
H	-4.533715	-2.333196	-4.431304
H	-4.961329	-3.743494	-5.417723
H	-3.597803	-3.846921	-4.287271
C	-6.438268	-3.982919	1.687408
C	-6.932405	-2.715020	2.402574
C	-5.583642	-4.819242	2.653197
C	-7.655401	-4.813719	1.275918
H	-7.546187	-2.985878	3.275909
H	-7.549533	-2.097519	1.731890
H	-6.099806	-2.092244	2.762573
H	-5.214815	-5.734610	2.164496
H	-6.182672	-5.118662	3.527444
H	-4.713317	-4.258782	3.026357
H	-8.328604	-4.255565	0.607390
H	-8.234177	-5.086145	2.171167
H	-7.365000	-5.749364	0.773529
C	6.122736	-4.408109	-1.542026
C	5.165452	-5.357525	-2.280304
C	7.358438	-5.203395	-1.115661
C	6.571104	-3.286807	-2.493214
H	5.672411	-5.810698	-3.146663
H	4.272819	-4.834528	-2.655461
H	4.828860	-6.171754	-1.619758
H	8.099928	-4.569975	-0.605111
H	7.846935	-5.629794	-2.004711
H	7.099865	-6.040021	-0.448515
H	5.719604	-2.702363	-2.873063
H	7.095733	-3.712651	-3.362942
H	7.259655	-2.591898	-1.987982
C	5.717981	-3.581311	3.487463
C	5.760361	-5.087084	3.793140
C	4.853999	-2.896567	4.548636
C	7.138509	-2.999399	3.567446
H	6.156002	-5.257228	4.806772
H	6.405614	-5.633359	3.089003
H	4.753321	-5.529568	3.742852
H	4.807408	-1.806574	4.401769
H	5.283038	-3.075399	5.545917
H	3.825313	-3.288856	4.557908
H	7.823512	-3.485191	2.856597
H	7.551145	-3.143392	4.578378
H	7.136544	-1.919762	3.351828
B	-2.339353	6.831606	1.280358
C	-1.682060	5.478125	1.879979
B	-4.275228	4.805539	0.172482
C	-3.250872	3.828678	0.970673
C	-0.214348	5.266314	2.099013
B	-5.077062	5.847479	1.364372
H	-6.245318	6.121666	1.240867
B	-2.433374	5.203807	0.375650
B	-3.774510	6.489537	0.307980
B	-2.845540	4.409281	2.518951
B	-4.587522	4.206211	1.824415
B	-2.654048	6.225673	2.931820
B	-3.947100	7.027894	2.014301
B	-4.279153	5.526948	2.939496
H	-4.331572	8.122240	2.344867
H	-5.319312	3.300353	2.132300
H	-2.457414	3.589345	3.315603
H	-2.154988	6.616507	3.956418
H	-4.879940	5.526114	3.984873
H	-4.754853	4.384616	-0.849904
H	-1.715670	5.025283	-0.577984
H	-1.582330	7.713896	0.965872
H	-4.001194	7.213272	-0.629123
H	0.041055	5.353675	3.165851
H	0.377518	6.008659	1.543985
H	2.336228	2.257183	-3.306056a
